# Composites of covalently linked lignin and cellulose from one feedstock

**DOI:** 10.1038/s41467-026-77206-8

**Published:** 2026-08-27

**Authors:** Shida Zuo, Lars William Schick, Suthawan Muangmeesri, Saranya Chitsomkhuan, Lenny Haddad, Joseph S. M. Samec

**Affiliations:** 1 Department of Chemistry, Stockholm University, Stockholm, Sweden; 2 Wallenberg Initiative Materials Science for Sustainability, Department of Chemistry, Stockholm University, Stockholm, Sweden

**Keywords:** Polymers, Polymer synthesis, Composites

## Abstract

Future biorefineries must upgrade all constituents of lignocellulosic feedstocks to high-value products. To curb land-use pressure, technologies that upgrade existing side-streams from forestry and agriculture into substitutes for high-impact, fossil-derived materials are urgently needed. Prevailing biomass valorization typically prioritizes either cellulose pulp or lignin; true co-valorization remains uncommon. Here we report a metal-free, ambient-pressure reactive fractionation that concurrently yields high-quality cellulose and a functionalized lignin. The isolated lignin is etherified and subsequently covalently coupled to cellulose via epoxide ring-opening, producing a composite. The materials display mechanical performance equivalent to common packaging materials, with retention under wet conditions, overcoming the intrinsic limitations of hydrogen-bonded cellulose networks. By integrating mild fractionation with chemical upgrading, this strategy simplifies processing, broadens the product slate accessible from residual biomass side streams and advances the substitution of problematic packaging materials. These findings establish a scalable route to whole-biomass co-valorization and wet-tolerant bio-based packaging from residual streams.

## Abstract (web summary)

Most lignocellulose derived materials utilise either lignin or cellulose, separating them and using them independently. Here, the authors produce a high strength composite material from lignocellulosic biomass which combines both lignin and cellulose, so neither fraction is wasted.

## Introduction

Global growth in population and increasing living standards continue to drive demand for materials; notably, the packaging market is expected to reach 1.33 trillion dollars by 2028^1^. These materials are sourced from both forestry and fossil feedstocks^1,2^. While projections indicate that conventional crude oil extraction will become economically constrained within the next 40 years due to exhaustion of resources, concerns are simultaneously rising over deforestation and the conversion of natural forests to plantations with low biodiversity^3^. Current forestry value chains remain inefficient: typically, less than half of harvested biomass is upgraded into high-value products, with the remainder left on site or diverted to low-value energy^4–6^. Although a portion of residual streams should be retained in forests to support soil re-carbonization and re-mineralization, a substantial fraction can be removed sustainably^7^. However, such streams are frequently contaminated during handling and exhibit elevated ash contents^8,9^. Similar challenges are seen in agricultural residues, which can contain up to 10 wt% ash^10–12^. High ash loads—rich in alkali and alkaline-earth metals—complicate thermochemical processing and deactivate or foul metal catalysts, limiting the applicability of many established and emerging upgrading routes^13–16^. Addressing ash-related contamination is therefore pivotal for converting forestry and agricultural side streams into higher-value materials, including next-generation packaging^17^.

Conventional pulping efficiently generates cellulose-rich pulps, while the remaining biomass is typically used to regenerate process chemicals and provide heat and power to the pulp mill^18,19^. Lignin can be precipitated from pulping liquors to afford technical lignins, but their native architecture is severely disrupted: condensation and oxidation deplete ether linkages and increase the fraction of *sp*²-hybridized aromatic C–C linkages at the expense of *sp*³-hybridized aliphatic bonds, yielding darker, less reactive and more rigid materials^20–22^. Consequently, materials produced from technical lignins often exhibit limited mechanical performance^23,24^. Over the past decade, alternative fractionation methodologies that focus on preserving lignin structure—collectively termed “lignin-first” and defined as “active stabilization approaches that solubilize lignin from native lignocellulosic biomass while avoiding condensation reactions that lead to more recalcitrant lignin polymers”—have emerged^25–28^. Despite being developed to raise the value of the entire feedstock, only a handful of studies holistically deliver high-value products from both lignin and cellulose^29,30^. Among lignin-first strategies, reductive catalytic fractionation (RCF) is the most extensively investigated: a transition-metal catalyst reduces reactive intermediates generated during lignin depolymerization to give high yields of monophenolic compounds^18,26,27,31–35^. A major challenge with the implementation of RCF is the ash content of biomass, especially residual streams, that would poison the transition metal catalyst^36^. In addition, identifying scalable applications for RCF monomers remains challenging; with few exceptions^37^, they are dimerized to furnish non-toxic bisphenols for materials applications^38–40^.

Barta and co-workers recently reported a lignocellulose-derived thermoset in which fractions from both lignin and cellulose were funneled into complementary building blocks and crosslinked with a bio-based epoxy^41^. In this scheme, vanillin was converted to 4,4′-methylenebis(cyclohexylamine) (MBCA), while the cellulose stream was transformed to dimethyl 2,5-furandicarboxylate (DMFD); curing the DMFD-based epoxide with MBCA yielded a high-performance network. The study demonstrated that the two principal biomass fractions can be integrated within a single materials platform and that the constituent building blocks are recoverable, enabling chemical recyclability.

**Fig. 1:**
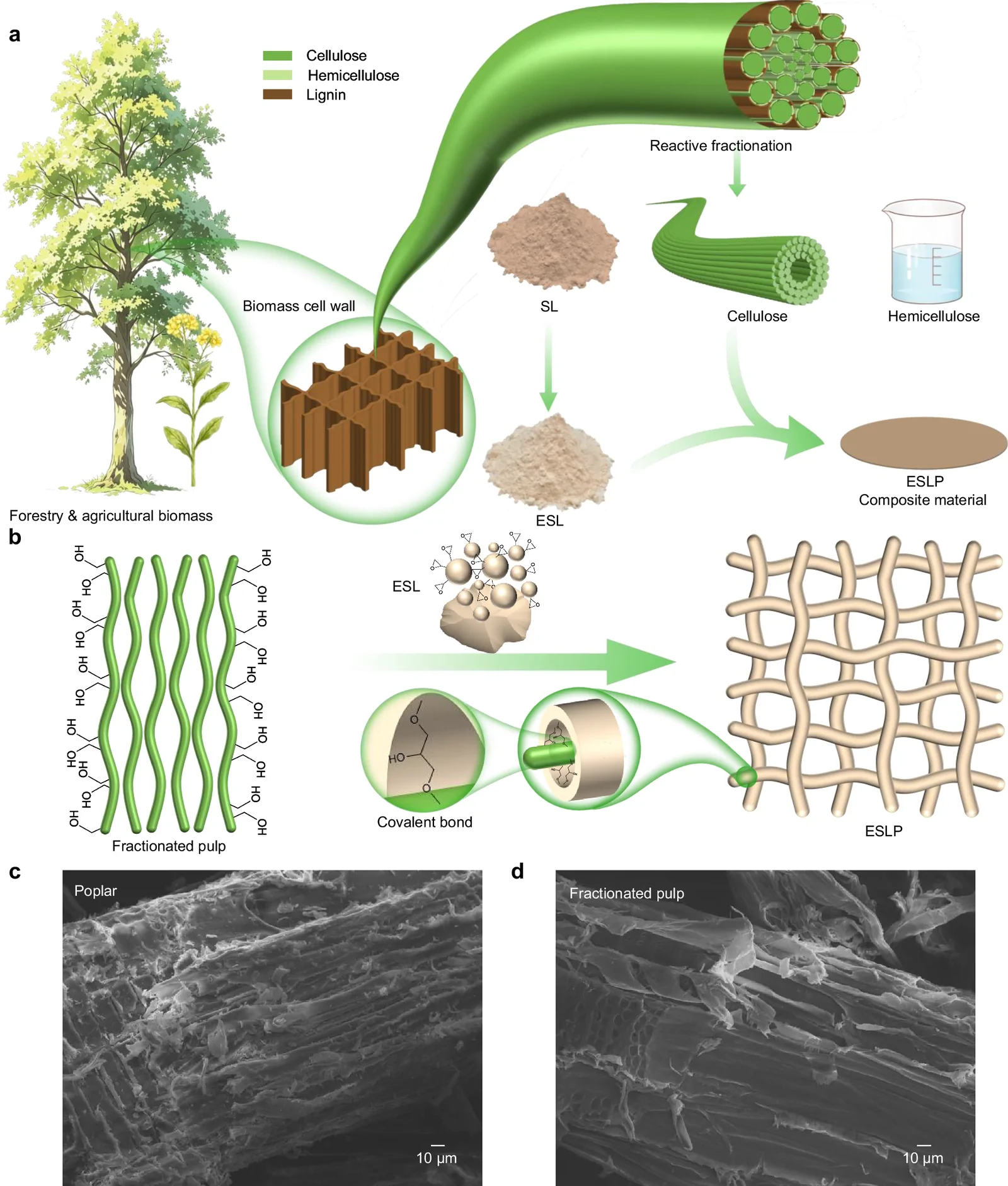
Reactive fractionation process and fabrication of composites. **a** Schematic illustration of reactive fractionation and **b** covalent bond between cellulose and ESL. SEM images of **c** natural poplar and **d** pulp obtained from reactive fractionation. SL Syringolated lignin, ESL epoxy-functionalized syringolated lignin, ESLP ESL-pulp composite.

Inspired by this approach, it was hypothesized—and subsequently demonstrated—that cellulose and lignin can be used without prior depolymerization to produce a thermoset material. The concept relies on a metal-free reactive fractionation performed at ambient pressure, during which lignin is converted in situ into a white, non-condensed, lipophilic syringolated lignin (SL) (Fig. 1a). The lipophilic SL is readily separated from the hydrophilic hemicellulose fraction and the solid pulp, then further functionalized by etherification to afford epoxy-functionalized syringolated lignin (ESL). Recombining ESL with cellulose yields a thermoset exhibiting exceptional wet-strength properties attributable to covalent bonding (Fig. 1b). All reactions proceed at ambient pressure and without metal catalysts, which makes the methodology amenable to scale-up. Moreover, the reaction conditions are insensitive to ash content, enabling the use of residual streams such as forestry tops and branches and agricultural stalks. Consequently, this methodology does not compete with current biomass valorization routes and delivers a material capable of substituting common packaging materials such as kraft liner from virgin forestry or polyethylene terephthalate (PET) from the petroleum industry.

## Results

### Reactive fractionation of biomass to obtain lignin with a chemical handle and cellulose with an accessible surface area

Reactive fractionation was performed on poplar wood (*Populus trichocarpa*) using syringol as the nucleophile and formic acid as the solvent at reflux (Fig. 1a)^42^. The process furnished two isolable streams: a light-colored soluble fraction, *vide infra*, and a cellulose-rich pulp readily separated by filtration that accounted for 46.9% of the initial biomass. The pulp contained 94.2% cellulose and 1.9% hemicellulose, with 3.3% lignin. By comparison, native poplar comprised 47.5% cellulose, 17.1% hemicellulose and 28.1% lignin (Table S1). The low residual hemicellulose content in the pulp indicates that hemicellulose was largely hydrolyzed into xylose and xylose-derived soluble species and transferred to the hydrophilic filtrate during the formic-acid-based fractionation^42^. In addition, xylose has been demonstrated as a suitable feedstock for formic acid production, providing a potential route for future process integration and solvent regeneration^43^. To evaluate the influence of syringol loading, biomass/syringol mass ratios of 1:0.2, 1:0.4, and 1:0.6 were further compared under otherwise identical conditions (Table S3). Increasing the syringol loading from 1:0.2 to 1:0.4 increased the SL yield from 34.0% to 35.8%, while maintaining a high pulp purity of 94.2%. A further increase to 1:0.6 led to a slightly higher SL yield of 37.7% and a pulp purity of 96.5%; however, this modest improvement required 50% more syringol. Therefore, the biomass/syringol ratio of 1:0.4 was selected as a balanced condition considering SL yield, pulp quality, reagent economy and downstream purification practicality. Together, these results indicate that the reactive fractionation achieves efficient delignification while delivering a functionalized lignin stream alongside exceptionally pure cellulose.

To elucidate how reactive fractionation alters the microstructure of native biomass, a scanning electron microscope (SEM) was used to compare wood fibers before and after treatment (Fig. 1c, d). Native fibers exhibited an intact hierarchical architecture^44^. In contrast, fibers in the fractionated pulp showed pronounced fibrillation, surface roughening and partial splitting along the fiber axis, indicative of weakened inter-fiber adhesion. These morphological changes are consistent with disruption of the lignin–carbohydrate complex (LCC) linkages, yielding smaller bundles and exposing microfibrils^45^. The resulting increase in accessible surface area is expected to enhance subsequent chemical functionalization and interfacial bonding with a resin in composite fabrication.

### Functionalizing the lignin fraction

**Fig. 2:**
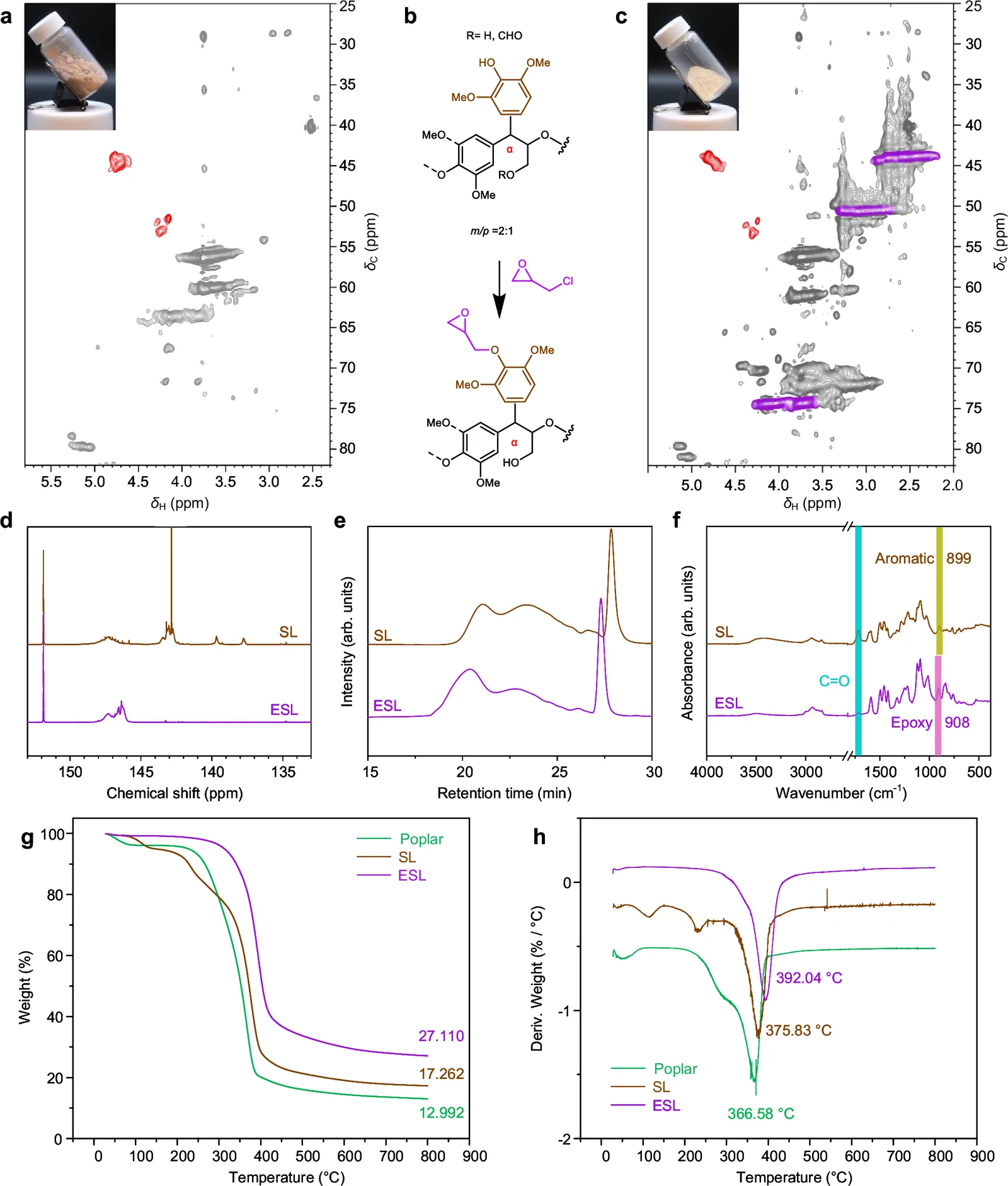
Characterizations of SL and ESL. HSQC NMR spectra of **a** SL and **c** ESL in DMSO-d_6_, showing syringyl aromatic correlations (red) and new cross-peaks (purple) assigned to epoxy methylene/methine groups. **b** Selective epoxy-functionalization of SL on phenolic hydroxyls. **d** ^31^P NMR spectra of SL and ESL in CDCl_3_ after phosphitylation, highlighting the disappearance of phenolic OH. **e** GPC curves of SL and ESL in tetrahydrofuran, indicating a shift toward higher molecular weight after epoxy-functionalization. **f** FTIR spectra of SL and ESL, showing the reduction of O–H stretching and the appearance of an epoxy ring absorption at 908 cm⁻¹. **g** TG and **h** DTG curves of poplar, SL, and ﻿ESL﻿ represent the thermal stability of samples. SL Syringolated lignin, ESL epoxy-functionalized syringolated lignin.

The SL was isolated by precipitation in 35.8% yield based on the initial poplar (theoretical maximum 38%). Running the reactive fractionation at reflux afforded a slightly brownish off-white solid (Fig. 2a), whereas lowering the temperature to 80 °C produced a whiter lignin but led to incomplete syringolation and thereby a lignin with reduced reactivity. The comparison between native poplar lignin and SL by heteronuclear single-quantum coherence (HSQC) NMR indicated successful arylation. For a clearer structural comparison, the HSQC features of native poplar lignin were first examined (Fig. S1). The native poplar lignin spectrum showed typical hardwood lignin correlations. In particular, the native β–O–4 Cα–Hα correlation at *δ*_C_/*δ*_H_ = 72.6/4.8 ppm, corresponding to the benzylic Cα–OH structure of native lignin, was clearly observed. After syringol-assisted reactive fractionation, this native Cα–OH correlation disappeared, while new correlations at *δ*_C_/*δ*_H_ = 44.8/4.7 and 53.9/4.2 ppm appeared, consistent with substituted Cα sites formed upon syringolation (Fig. 2a)^42^. This comparison confirms that syringol effectively trapped the benzylic intermediate during fractionation and converted native lignin into syringolated lignin. Quantitative ^31^P NMR further corroborated syringolation, showing a distinct and sharp resonance at *δ*_P_ = 142.85 ppm within the C–5–substituted phenolic region, which is consistent with syringolation at the Cα position (Fig. 2d)^46^. The *meta/para* (*m/p*) ratio of SL was estimated from the HSQC spectrum by integrating the diagnostic Cα–Hα correlations corresponding to *meta*- and *para*-syringolated β–O–4 structures. The calculated *m/p* ratio was approximately 2:1, indicating that syringol addition occurred at both regioisomeric positions, with the *meta*-substituted structure being more abundant under the present reactive fractionation conditions. This regioisomeric distribution further supports the nucleophilic trapping of the benzylic Cα intermediate by syringol during fractionation. SL displayed a high abundance of phenolic OH groups (3.22 mmol g⁻¹), underscoring its reactive potential following syringolated fractionation. In addition, the HSQC spectra of SL obtained using biomass/syringol mass ratios of 1:0.2, 1:0.4, and 1:0.6 showed comparable diagnostic Cα–Hα correlations assigned to the syringolated structures, indicating no obvious difference in the apparent extent of arylation within the investigated loading range (Figs. S2–4).

Mechanistically, formic-acid-mediated cleavage of β–O–4 linkages generates reactive benzylic Cα intermediates that can undergo acid-induced hydrolysis and undesirable lignin condensation in the absence of an external trapping reagent^42^. The FA-only fractionated lignin retained negligible β-ether units because of acidic hydrolysis and non-selective condensation, whereas syringol directs the reaction toward selective α-arylation (Fig. S5). In contrast, SL displayed clear substituted Cα correlations together with the disappearance of the native Cα–OH signal, confirming that syringol selectively trapped the reactive Cα intermediate and suppressed uncontrolled lignin self-condensation (Fig. 2a).

The reactive lignin was then functionalized by reaction with epichlorohydrin, which can be sourced from biomass^47^. Selective etherification of SL at its phenolic hydroxyl groups afforded the corresponding ESL (Fig. 2b). Notably, precipitation of the ESL yielded an off-white material in a quantitative yield starting from the brownish lignin (Fig. 2c). The HSQC spectrum of ESL displayed the additional cross-peaks in the aliphatic region, assignable to epoxy methylene and epoxy methine groups^48^ (Fig. 2c). The coexistence of introduced syringyl correlations and these new epoxy signals verifies the introduction of epoxide functionality while retaining the syringolated backbone. In line with this assignment using ^31^P NMR, phenolic OH decreased from 3.22 to 0.01 mmol g⁻¹ after epoxy-functionalization, whereas aliphatic OH increased from 1.70 to 2.41 mmol g⁻¹, consistent with cleavage of γ–formyl groups introduced during reactive fractionation and regeneration of aliphatic hydroxyls (Fig. 2d)^49^. Gel permeation chromatography (GPC) analysis revealed a shift of both apparent molecular-weight fractions toward higher molecular weights after epoxy functionalization (Fig. 2e and Table S4). For the higher-molecular-weight fraction, *M*ₙ and *M*_w_ increased from 1431 and 1521 Da for SL-1 to 1967 and 2234 Da for ESL-1, respectively. For the lower-molecular-weight fraction, *M*ₙ and *M*_w_ increased from 476 and 572 Da for SL-2 to 678 and 725 Da for ESL-2, respectively. The dispersity remained relatively narrow for all fractions (Đ = 1.06–1.20), indicating that epoxy functionalization increased the apparent molecular size without causing pronounced broadening of the molecular-weight distribution. Fourier transform infrared (FTIR) spectroscopy spectra further corroborated these assignments (Fig. 2f). Relative to SL, ESL shows attenuation of the broad O–H stretch near 3400 cm⁻¹ and emergence of a characteristic epoxide band at 908 cm⁻¹, consistent with epoxide formation and with the concomitant decrease in phenolic hydroxyl content observed by ^31^P NMR^50,51^. The tiny shoulder around 3056 cm⁻¹ represents the C–H vibration of the epoxide ring, which supports the success of epoxy-functionalization as well^52,53^. A marked reduction in intensity near 1720 cm⁻¹ additionally supports cleavage of γ–formyl groups during epoxy-functionalization^54^. Taken together, the HSQC, ^31^P NMR, GPC and FTIR results indicate that SL retains reactive phenolic functionality after reactive fractionation and that epoxy-functionalization efficiently converts these sites to epoxide groups while only modestly shifting the molecular-weight envelope, yielding a tunable lignin precursor suitable for subsequent composite fabrication. Chemical modification of lignin shifted the principal pyrolytic event to a higher temperature. Thermogravimetric analysis (TGA/DTG) provided complementary evidence on the thermal property (Fig. 2g–h). The DTG maximum shifted from 366.58 °C for native poplar to 375.83 °C for SL and further to 392.04 °C for ESL, establishing ESL as the most thermally stable among the series. Reactive fractionation leads to a seemingly more thermally stable lignin, possibly by preventing degradation at the Cα position, thereby suppressing the phenolic β–O–4 quinone-methide pathway, which requires Cα–OH^55^. Epoxy-functionalization further blocks almost all phenolic sites, which impede radical initiations known for lignin degradation pathways^55,56^.

### Formation of a covalent bond between ESL and cellulose to yield a high-strength composite

**Fig. 3:**
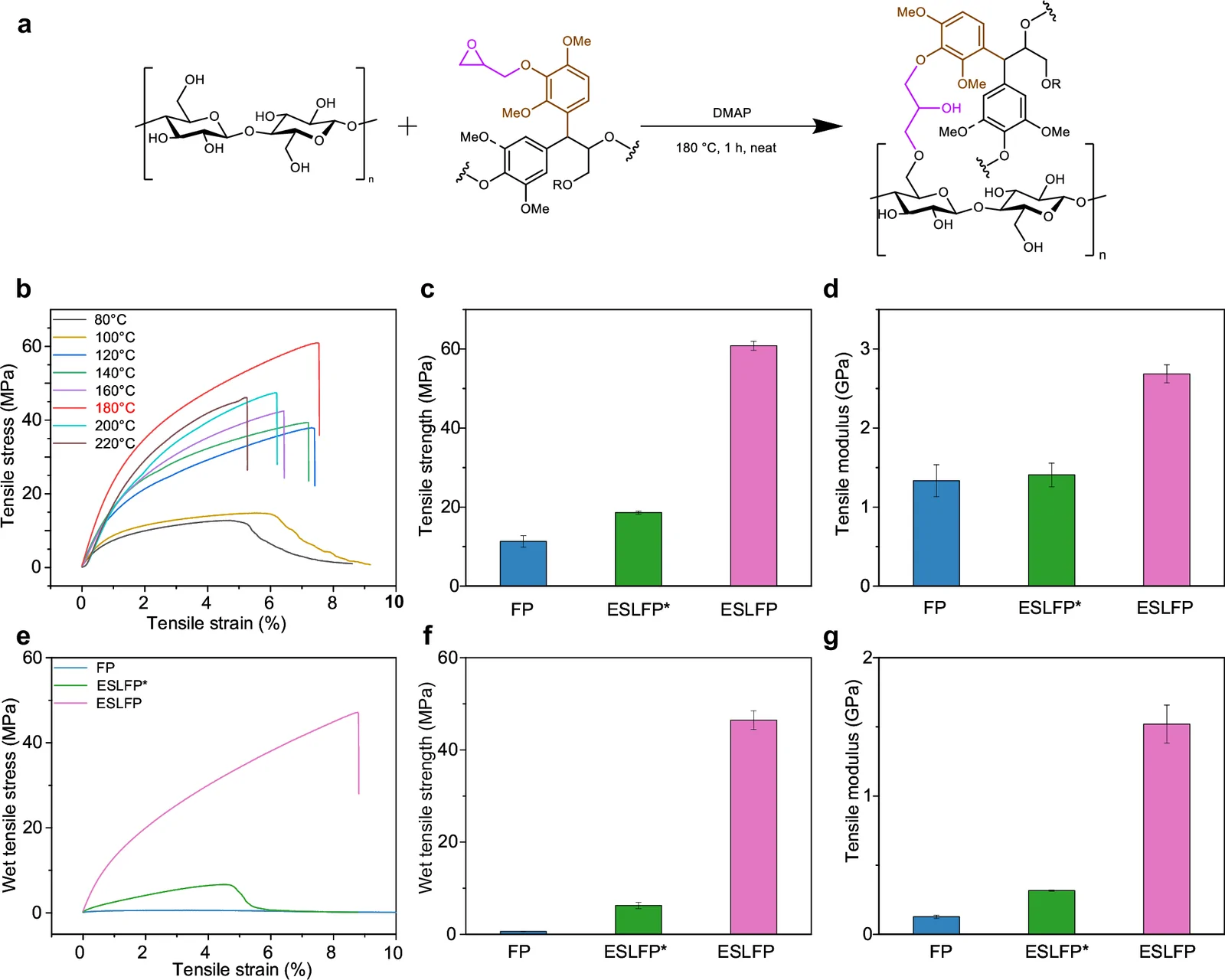
Mechanical properties of ESLFP. **a** Proposed reaction scheme between cellulose and ESL. **b** Tensile stress-strain curves of ESLFP prepared under different temperatures. **c** Tensile strength and **d** modulus of FP, ESLFP*, ﻿and ESLFP. **e** Wet tensile stress-strain curves of FP, ESLFP*, and ESLFP. **f** Wet tensile strength and **g** modulus of FP, ESLFP*, ﻿and ESLFP. The error bars represent the standard deviation of data from three replicate measurements. ESL Epoxy-functionalized syringolated lignin, FP filter paper, ESLFP ESL-filter paper composite, ESLFP* ESL-filter paper composite without catalyst.

We hypothesized that the C–OH of cellulose reacts with the epoxide of ESL to form new ether linkages (Fig. 3a), to give rise to inter-fiber crosslinking. Filter paper (FP) was used as a cellulose model while screening curing temperatures (80 °C–220 °C) in the presence of 4-dimethylaminopyridine (DMAP). The tensile strength of ESL–filter paper sample (ESLFP) increased with curing temperature, reaching 60.8 MPa at 180 °C and then declining at higher temperatures, consistent with the onset of cellulose thermal degradation (Fig. 3b). At this optimum, the composite was sixfold stronger than FP (11.3 MPa), evidencing robust ESL–cellulose interactions (Fig. 3c). The role of DMAP was demonstrated using a no-catalyst control (ESLFP*). Without DMAP, the ESLFP* sample exhibited much lower strength (18.6 MPa), because unreacted ESL was removed during purification, indicating that physical adsorption alone is insufficient to provide load-bearing interfaces. Stiffness followed the same trend: the tensile modulus rose from 1.3 GPa (FP) to 1.4 GPa (ESLFP*) and to 2.7 GPa (ESLFP), consistent with covalent bonding limiting inter-fiber slippage under load (Fig. 3d). Water resistance further differentiates covalently bonded material from the ESLFP* with other weaker physical interactions. ESLFP retained 46.5 MPa wet tensile strength (76.5%), whereas ESLFP* exhibited negligible wet strength of 6.3 MPa (33.9% retention) (Fig. 3f). The wet tensile modulus similarly increased from 0.1 GPa (FP) to 0.3 GPa (ESLFP*) and 1.5 GPa (ESLFP) (Fig. 3g). These improvements are attributed to DMAP-catalyzed epoxide ring-opening at cellulose C–OH, which (i) creates covalent ESL–cellulose bridges that persist in water, (ii) reduces swelling-induced plasticization by anchoring the interphase and (iii) enhances stress transfer through a stiffer, chemically bonded network. Collectively, the data identify DMAP-catalyzed covalent bond formation at 180 °C as the governing factor underpinning both the sixfold increase in dry strength and the high wet-strength and modulus retention of ESLFP.

**Fig. 4:**
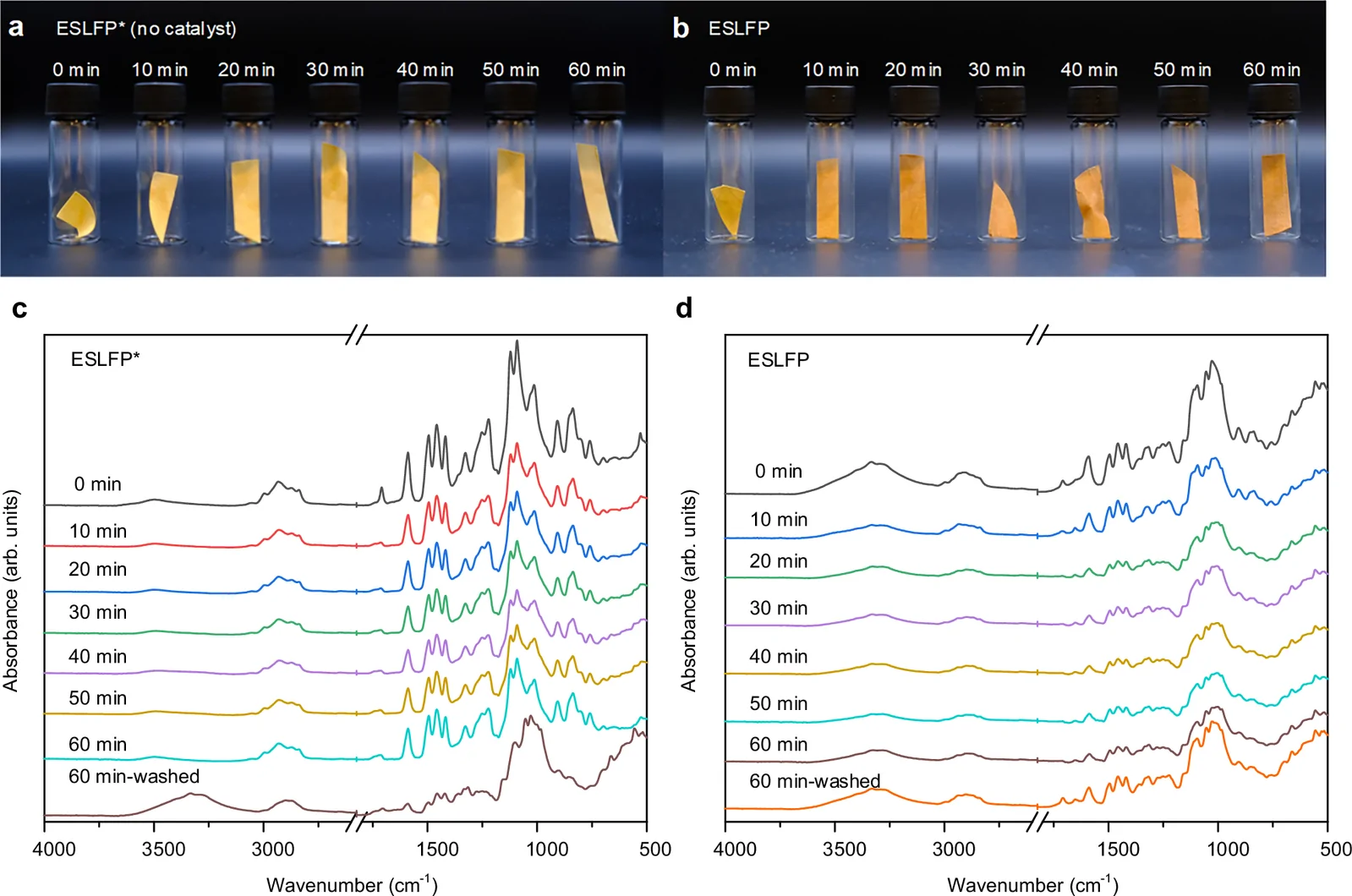
Kinetic experiments of ESLFP* and ESLFP. Photographs of **a** ESLFP* and **b** ESLFP heated for different durations. Kinetic FTIR spectra of **c** ESLFP* and **d** ESLFP heated for different durations. ESL Epoxy-functionalized syringolated lignin, ESLFP ESL-filter paper composite, ESLFP* ESL-filter paper composite without catalyst.

Macroscopic observations (Fig. 4a, b) corroborate the chemistry: in the presence of DMAP, ESLFP darkened relative to both ESLFP* and the non-heated sample. Additional acetone washing after heating removed excess, unreacted ESL, rendering ESLFP* lighter in color, whereas the appearance of ESLFP was unaffected (Fig. S6a, b). Kinetic FTIR analysis was employed to analyze the reaction process between cellulose and ESL, and the spectra reveal time-dependent changes in the chemical composition of ESLFP and ESLFP* heated for different durations (Fig. 4c, d). As heating proceeded, the epoxy band of ESLFP at 908 cm^−1^ in the ESLFP sample gradually decreased in intensity and red-shifted towards 898 cm^−1^, indicating consumption of epoxy groups and the re-emergence of the β–1,4–glycosidic bond vibration of cellulose^50,51,57^. In contrast, the epoxy band in ESLFP* remained essentially unchanged, consistent with the design of a no reaction system in the absence of the catalyst.

Quantitative analysis of the integrated epoxy band using an apparent pseudo-first-order model gave apparent rate constants of 0.00755, 0.00865, and 0.00927 min⁻¹ at 140 °C, 160 °C, and 180 °C, respectively (Table S5). Fitting the FTIR-derived apparent rate constants according to an Arrhenius-type relationship yielded an apparent activation energy, *E*ₐ_,app_, of 8.03 kJ mol⁻¹, with an *R*² of 0.975. The relatively low *E*ₐ_,app_ primarily reflects the modest temperature dependence of the averaged FTIR-derived apparent rate constants over the investigated temperature range. This value should be interpreted in the context of the heterogeneous ESL–cellulose interfacial reaction rather than as the intrinsic activation barrier of an elementary epoxide ring-opening reaction. In this solid or softened-solid/solid system, epoxide groups on ESL and hydroxyl groups on cellulose are confined to different phases. Bond formation, therefore, requires ESL softening and segmental mobility, interfacial wetting, local contact between reactive groups, as well as accessibility of cellulose hydroxyls. Solid-state reactions are often affected by precursor architecture, interfacial contact and transport within solid phases, while cellulose possesses a rigid, hydrogen-bonded microfibrillar structure in which hydroxyl groups are not equally mobile or accessible^58–60^. Increasing temperature can therefore promote early-stage epoxide conversion, ESL mobility, wetting and fiber–fiber interfacial contact, facilitating the formation of a more continuous load-bearing network during the fixed curing period. However, once the readily accessible interfacial epoxy groups have reacted, further conversion may become increasingly limited by contact, accessibility and restricted molecular mobility rather than solely by the chemical ring-opening barrier. Previous studies of solid-state reactions, cellulose structure and accessibility, cellulose etherification and complex epoxy curing systems have similarly emphasized the roles of interfacial contact, restricted mobility and hydroxyl accessibility^61–64^. Therefore, *E*ₐ_,app_ is regarded as a semi-quantitative apparent descriptor of the overall ESL–cellulose interfacial curing process, rather than a true activation energy for a single elementary reaction.

**Fig. 5:**
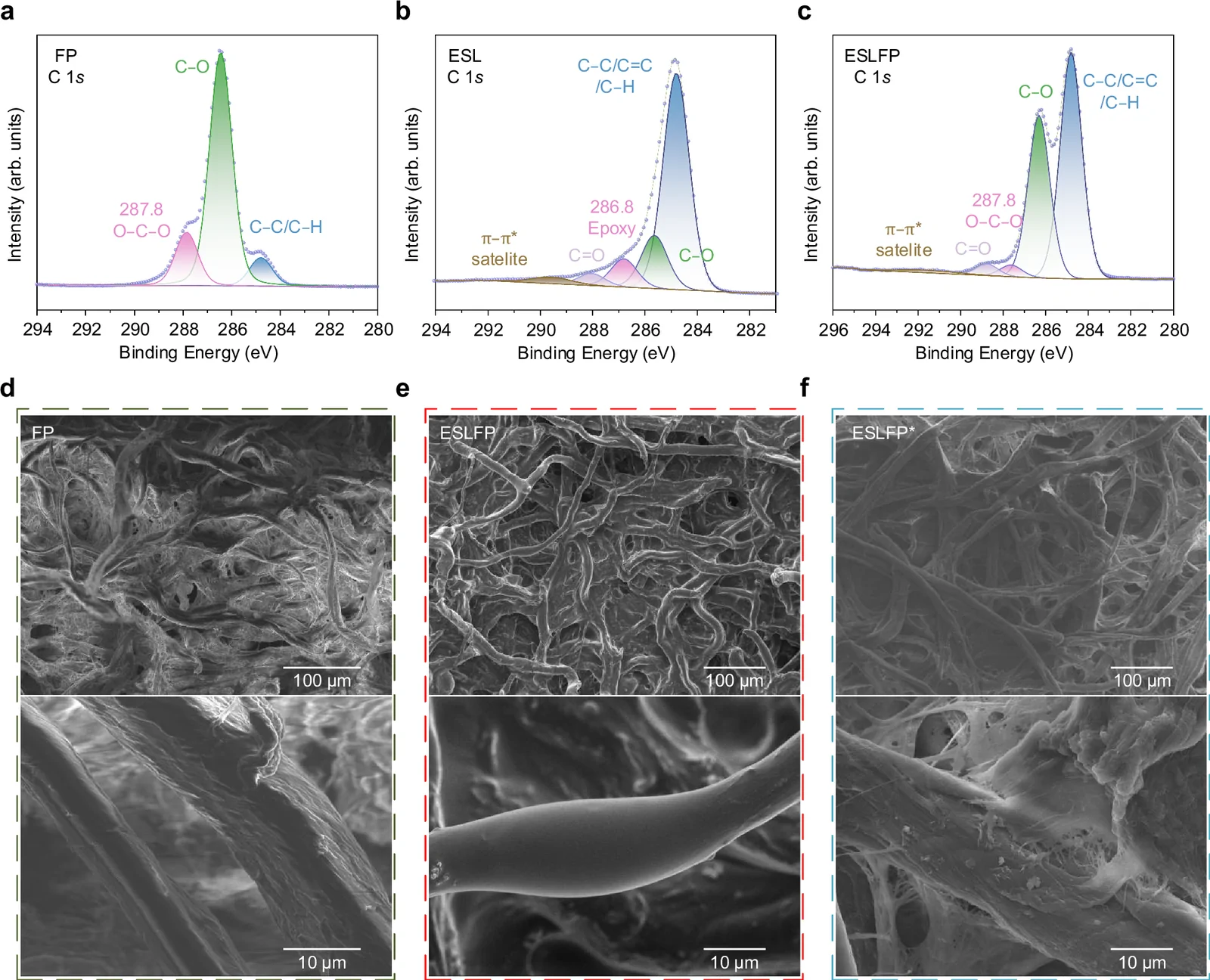
Surface observations of FP, ESLFP*, and ESLFP. XPS spectra of **a** FP, **b** ESL, and **c** ESLFP. SEM images of **d** FP, **e** ESLFP, and **f** ESLFP*. ESL Epoxy-functionalized syringolated lignin, FP filter paper, ESLFP ESL-filter paper composite, ESLFP* ESL-filter paper composite without catalyst.

To probe the interactions underpinning the mechanical enhancement, X-ray photoelectron spectroscopy (XPS) was used to monitor surface functional groups. The high-resolution C 1*s* spectrum of FP shows the expected dominance of the C2 component (C–O, 286.5 eV), reflecting the abundance of alcohol and ether carbons in the polysaccharide backbone (Fig. 5a). By contrast, ESL exhibits a spectrum dominated by the C1 component (C–C/C–H/C=C, 284.8 eV), consistent with aromatic and aliphatic carbons of lignin (Fig. 5b). A distinct contribution near 286.8 eV is observed for ESL, consistent with the presence of the epoxide functionality and aligning with the HSQC evidence for epoxide formation (Fig. 2c). Upon forming the ESLFP composite, the C1 fraction increases relative to cellulose, while the C2 fraction increases relative to ESL, indicating coexistence and intermixing of ESL and cellulose at the surface level (Fig. 5c). Notably, the epoxide-associated contribution near 286.8 eV diminishes after curing, consistent with ring-opening of the epoxide with cellulose C–OH groups rather than mere physical mixing. Together, the C 1*s* evolutions support chemical coupling at the interface, corroborating the mechanical gains observed for ESLFP.

FTIR was used to assess bulk chemical changes since XPS probes only the near-surface chemical compositions. Neat ESL displays the epoxide ring vibration at 908 cm⁻¹, whereas FP, ESLFP* and ESLFP show a band at 896 cm⁻¹ assigned to the cellulose β–1,4–glycosidic linkages^50,51,57^. In the DMAP-cured composite (ESLFP), the disappearance of the 908 cm⁻¹ epoxide band—together with the persistent 896 cm⁻¹ cellulose band—indicates epoxide ring-opening and formation of new ether linkages between ESL and cellulose (Fig. S7)^50,51,57^.

SEM further supported the ESL-cellulose ether bond. FP displays fibers with the characteristic microscale roughness of native cellulose (Fig. 5d). After incorporation of ESL, ESLFP shows fibers with a continuous, conformal coating and smoother inter-fiber interfaces, indicative of an interphase that bridges neighboring fibers (Fig. 5e). In contrast, ESLFP* resembles the native fiber morphology after acetone purification, consistent with removal of unreacted ESL and limited interfacial bonding (Fig. 5f). The morphological evidence—continuous coating in ESLFP versus fiber-like surfaces in ESLFP*—is consistent with the covalent coupling inferred from XPS and FTIR and explains the observed enhancements in mechanical strength, modulus and wet-strength retention.

### Fabrication and characterization of pulp–ESL composites

RCF is highly effective for producing lignin-derived aromatic monomers or oligomers, but typically relies on transition-metal catalysts, reductive conditions, catalyst recovery and downstream product separation or upgrading. These requirements can be challenging for ash-rich residual feedstocks because alkali and alkaline-earth metal species may poison or foul metal catalysts. In contrast, the present metal-free process does not rely on surface metal catalytic sites and directly yields a reactive lignin stream and cellulose pulp that can be recombined into a covalently bonded composite. To demonstrate that reactive fractionation is feasible for ash-enriched feedstocks, we applied the methodology to rapeseed straw, which contains 2% of ashes. The process afforded SL in a yield of 24.8% based on dry biomass and a paler off-white solid ESL (Figs. S8–10). The resulting composite reached tensile strengths of 40.0 MPa in the dry state and 21.8 MPa under wet conditions, compared with 11.9 and 1.5 MPa, respectively, for the corresponding neat pulp (Fig. S11, ST. 1). XRD analysis of the rapeseed-straw ash indicated representative K- and Ca- containing inorganic phases (Fig. S12a, ST. 2). Based on that, an ash-mimic experiment also gave an SL yield of 35.5% and pulp purity of 96.7%, comparable to the salt-free condition (ST. 3).

The successful use of rapeseed straw also highlights a key distinction between the present strategy and conventional lignin-first approaches such as RCF (Table S6). Thus, this strategy is complementary to RCF: rather than targeting aromatic monomer production, it provides a material-oriented route for direct cellulose–lignin co-valorization from residual biomass streams.

**Fig. 6:**
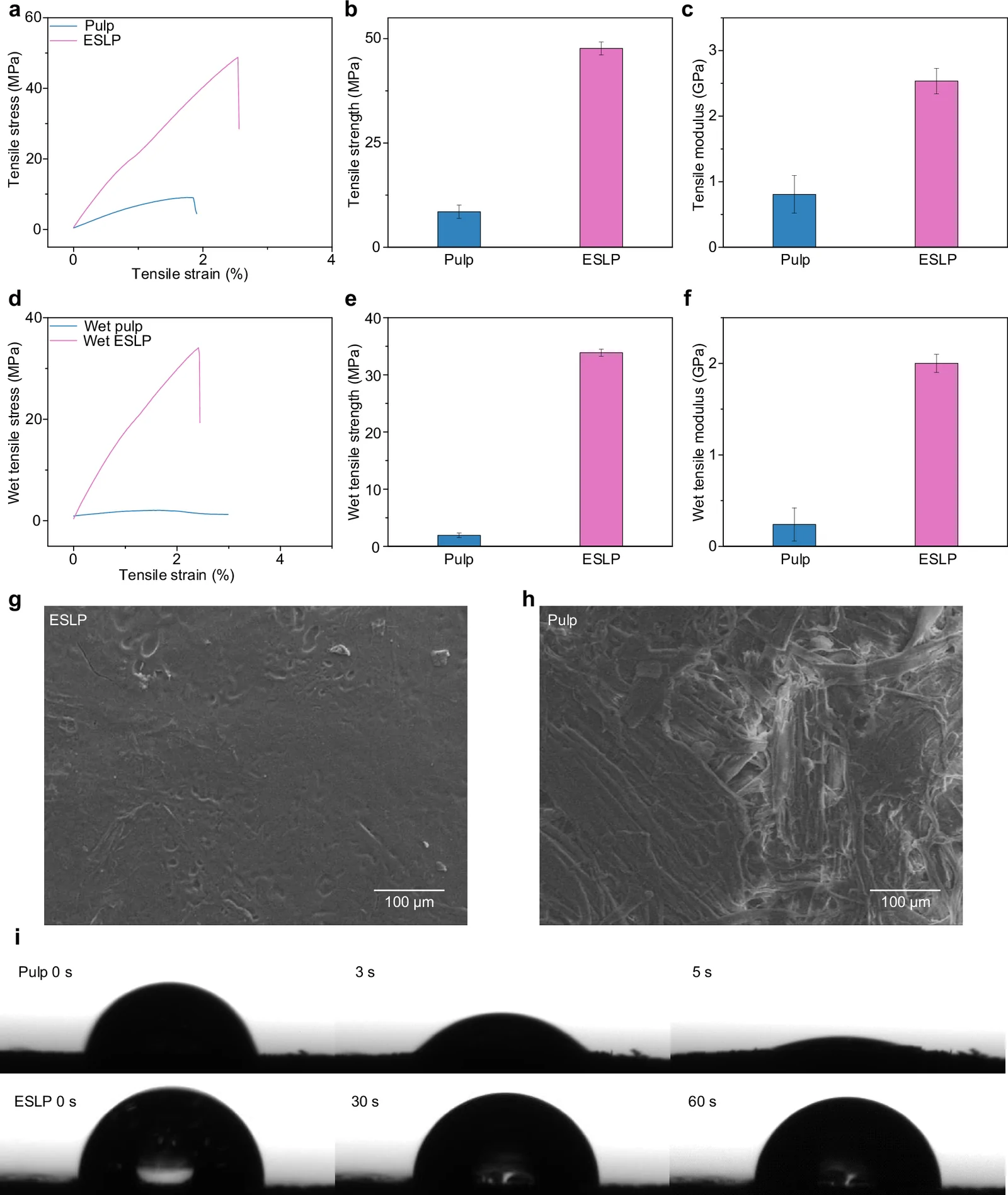
Characterizations of ESLP. **a** Tensile stress-strain curves of ESLP and pulp. **b** Tensile strength and **c** modulus of ESLP and pulp. **d** Wet tensile stress-strain curves of ESLP and pulp. **e** Wet tensile strength and **f** modulus of ESLP and pulp. SEM images of **g** ESLP and **h** pulp. **i** Contact angle images of ESLP and pulp after different times. The error bars represent the standard deviation of data from three replicate measurements. ESL Epoxy-functionalized syringolated lignin, ESLP ESL-pulp composite.

The same covalent chemistry was translated to pulp obtained from reactive fractionation of poplar: without additional chemical treatment or bleaching, cellulose pulp, ESL and DMAP were dispersed in DI water, homogenized and hot-pressed to drive epoxide ring-opening at cellulose C–OH, forming the ether linkages. The resulting ESLP exhibited a tensile strength of 46.7 MPa and modulus of 2.5 GPa, markedly higher than the neat pulp sheet prepared under the same hot-pressing conditions (8.5 MPa and 0.8 GPa, respectively) (Fig. 6a–c). These gains confirm that the covalent-bonding strategy operative in the model system also functions with fractionated pulp, demonstrating concurrent valorization of lignin and cellulose into one material. ESLP maintained 33.9 MPa wet tensile strength and a wet modulus of 2.0 GPa, whereas pure pulp retained only 1.9 MPa and 0.2 GPa (Fig. 6d–f). Because the neat pulp control was subjected to the same hot-pressing process, this comparison indicates that densification alone cannot account for the large improvement in mechanical performance, especially under wet conditions. The high wet-strength retention of ESLP therefore supports that covalent ESL–cellulose coupling, rather than hydrogen bonding alone, governs the durable inter-fiber adhesion in moist environments.

XRD analysis was further performed to examine whether the cellulose crystalline structure changed after fractionation and ESLP formation (Fig. S12b). The relative crystallinity index increased from 43.3% for native poplar to 57.7% for the fractionated pulp, consistent with the removal of amorphous lignin and hemicellulose during reactive fractionation. After ESL incorporation and hot pressing, ESLP showed a relative crystallinity index of 53.4%, slightly lower than that of pulp but still higher than that of native poplar. This slight decrease is attributed to the amorphous ESL phase contributing to the composite background rather than the destruction of the cellulose crystalline domains. These results indicate that the cellulose crystalline framework was retained in ESLP, while the improved mechanical and wet mechanical performance mainly arises from covalent ESL–cellulose coupling and enhanced inter-fiber adhesion rather than increased cellulose crystallinity.

Consistent with ESL–cellulose coupling, SEM images reveal a conformal, micro-crosslinked interphase in ESLP analogous to that observed in ESLFP (Fig. 6g). Relative to filter paper, the fractionated pulp offers greater accessible surface area and porosity; ESL therefore penetrates more deeply into the fiber network prior to curing, yielding continuous coatings and bridging at fiber-fiber contacts. The observed coated fibers and bridged inter-fiber interfaces in ESLP align with the enhanced dry and wet mechanical properties. By contrast, the hot-pressed pulp retained a more exposed fiber morphology, consistent with its lower dry and wet mechanical properties ﻿(Fig. 6h).

Hot-pressing produced a smoother, denser surface, and ESL incorporation reduced surface wettability. Static water contact angles started from 75.8° at 0 s (pure pulp) and rapidly decayed to 17.9° at 5 s, whereas ESLP started at 88.4° (0 s) and remained 85.0° at 60 s (Fig. 6i). The higher and time-stable contact angle indicates suppressed wetting, attributable to the covalently bonded ESL interphase together with surface densification—trends consistent with the improved strength, stiffness and wet-strength retention.

### Performance, durability, and application potential of ESLP

**Fig. 7:**
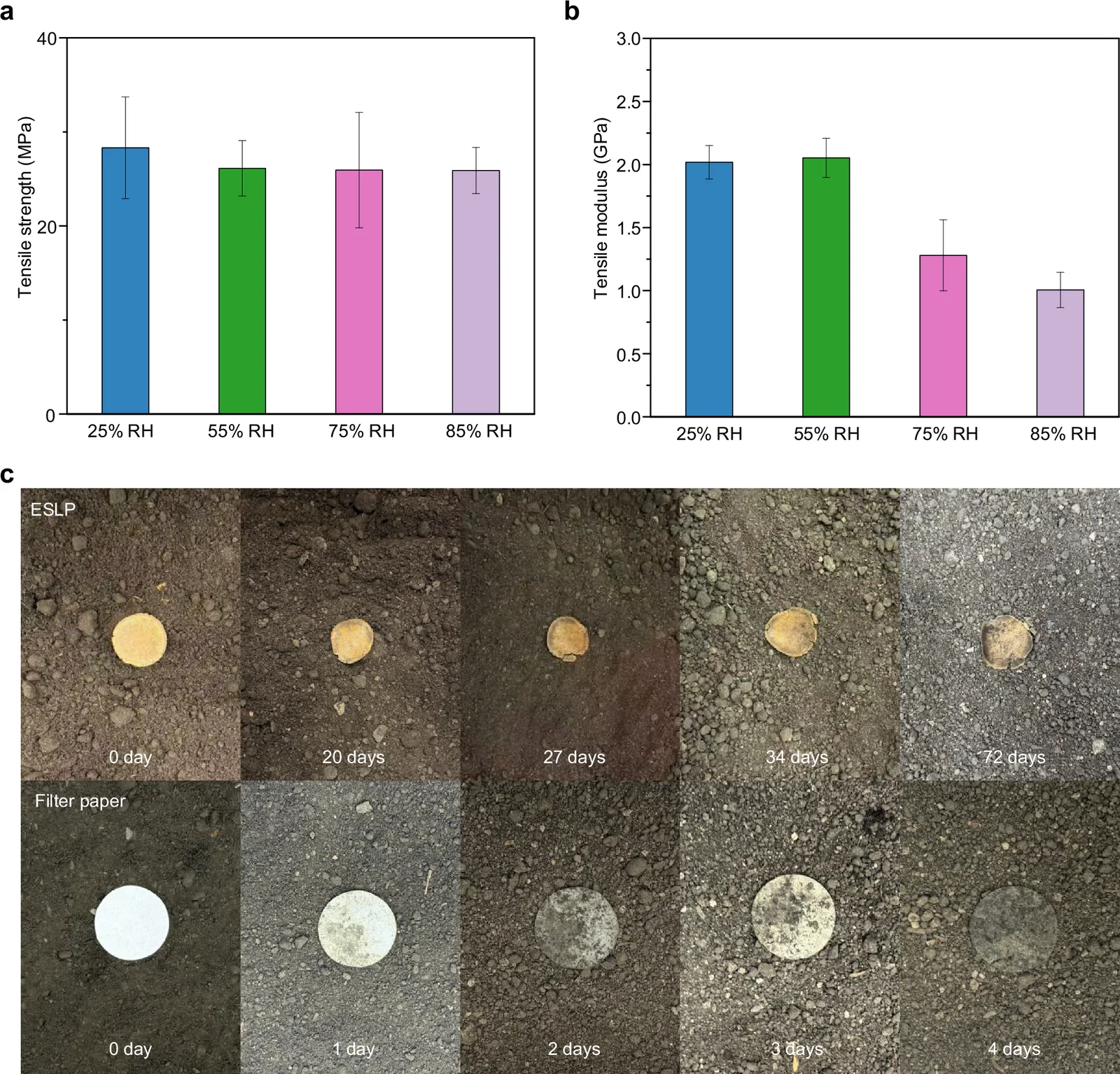
Long-term humidity stability and composting degradation behavior of ESLP. **a** Tensile strength and **b** tensile modulus of ESLP after aging for 97 days at different relative humidities at room temperature. **c** Representative photographs showing the macroscopic disintegration of ESLP and filter paper during composting. The error bars represent the standard deviation of data from three replicate measurements. ESL Epoxy-functionalized syringolated lignin, ESLP ESL-pulp composite.

While the contact-angle and wet-mechanical measurements demonstrate the immediate resistance of ESLP to water exposure, its practical use also requires retention of mechanical integrity during prolonged exposure to humid environments. The long-term humidity stability of ESLP was further evaluated after aging for 97 days at 25%–85% RH (Fig. 7a, b). The aged samples retained tensile strengths of 25.9–28.3 MPa, corresponding to approximately 55–61% of the original value, with no clear humidity-dependent decrease across the investigated range. In contrast, the tensile modulus remained approximately 2.0 GPa after aging at 25% and 55% RH but decreased to 1.28 and 1.01 GPa at 75% and 85% RH, respectively. The lower modulus observed after aging at 75% and 85% RH suggests that prolonged high-humidity exposure induced residual or only partially reversible changes in the cellulose-rich network. Nevertheless, the substantial tensile strength retained after 97 days indicates that the covalently coupled ESL–cellulose network maintains long-term load-bearing integrity under prolonged humidity exposure.

The moisture-sorption behavior of ESLP during long-term humidity exposure was also monitored (Fig. S13). Water uptake increased rapidly during the initial stage and subsequently approached a relatively stable level within the first few days. As expected, the water uptake increased with relative humidity, reaching approximately 1.5%–2.0%, 4.1%–4.5%, 5.0%–5.5%, and 6.8%–7.3% under nominal RH conditions of 25%, 55%, 75%, and 85%, respectively. After the initial increase, no pronounced continuous mass gain was observed during the remaining exposure period, indicating that the specimens reached a relatively stable moisture-sorption state. The humidity-dependent water uptake confirms that the cellulose-rich composite remains slightly hygroscopic, particularly at elevated RH, where absorbed moisture can promote plasticization and increase molecular and inter-fiber mobility. Nevertheless, the limited increase in water uptake over prolonged exposure suggests that the covalently coupled ESL–cellulose structure restricted progressive moisture-induced disruption of the material.

Having established that ESLP reached a stable moisture-sorption state while retaining substantial mechanical integrity during prolonged humidity exposure, we next examined whether this service-stage durability could coexist with biological degradation under an appropriate end-of-life environment. In the compost-burial experiment, the filter-paper control underwent rapid macroscopic deterioration within four days and was no longer visually detectable after 5 days, whereas ESLP showed gradual surface discoloration, deformation, erosion and partial fragmentation over 72 days (Fig. 7c). The slower disintegration of ESLP is consistent with its lignin-containing and covalently coupled network. In a separate compost-respiration experiment, the blank-corrected cumulative CO₂ evolution increased progressively and reached 920.7 mg after 65 days (Fig. S14), indicating enhanced microbial respiration associated with ESLP addition. Because the measurement was based on extrapolation of a 2 h respiration rate to a long-term equivalent, and the ESLP addition may stimulate the decomposition of native compost organic matter, the cumulative value is not interpreted as an absolute value derived exclusively from ESLP carbon^65–67^. Thus, the burial and respiration experiments provide complementary evidence of macroscopic disintegration and biological degradation, respectively. These results indicate that ESLP can maintain functional integrity under service-relevant humidity conditions while undergoing progressive degradation under an appropriate composting environment.

Taken together, these results demonstrate that ESLP combines service-stage mechanical and moisture stability with end-of-life degradability under composting conditions, providing a useful performance basis for applications beyond conventional packaging. Beyond packaging, the combination of mechanical strength, wet-strength retention, long-term humidity stability and composting degradability suggests that ESLP may serve as a broader wet-stable bio-based materials platform. For example, the retained load-bearing capacity under humid conditions could be useful for molded fiber products, wet-resistant disposable articles and agricultural materials that require temporary mechanical integrity during use but eventual degradation after disposal. The covalently bonded lignin–cellulose network may also provide a basis for future filtration supports or water-treatment membrane substrates where dimensional stability in wet environments is required. However, such applications would require additional control over porosity, permeability, separation performance, and, for biomedical-related uses, cytocompatibility and safety. These directions are therefore regarded as future opportunities rather than demonstrated applications in the present study.

Several practical challenges remain before industrial implementation. Feedstock variability may require screening, drying, size control and compositional monitoring. Efficient recovery of epichlorohydrin, together with improved filtration and washing, will be important for reducing reagent consumption and separation costs. In addition, the current hot-pressing condition should be optimized by shortening the curing time, tuning catalyst loading and improving heat transfer. Thus, although the metal-free, ambient-pressure process provides a promising basis for scale-up, further process optimization and techno-economic assessment are required.

To contextualize the packaging potential of ESLP, its performance was compared with representative PLA, lignin-containing cellulose nanopaper, kraft paper and PET film (Table S7). ESLP exhibited a tensile strength and modulus of 46.7 MPa and 2.5 GPa, respectively, which are within the representative ranges of widely used packaging materials. Its distinguishing feature is its moisture-resistant mechanical performance: ESLP retained 33.9 MPa wet tensile strength and a wet modulus of 2.0 GPa, corresponding to 72.6% and 80.0% retention, respectively. These strength-retention values are higher than those reported for the representative lignin-containing cellulose nanopaper and kraft paper included in the comparison. ESLP also showed a DTG maximum at 384.8 °C (Fig. S15) and retained tensile strengths of 25.9–28.3 MPa after 97 days of humidity aging. Together with its progressive macroscopic deterioration and blank-corrected CO₂ evolution under composting conditions, these results demonstrate a balance between service-stage moisture stability and end-of-life biological degradability.

## Discussion

In conclusion, this work presents a fully bio-based composite material that achieves high mechanical strength and remarkable wet stability enabled through covalent bond formation between lignin and cellulose without prior depolymerization. The key lies in utilizing both major biomass fractions—lignin and cellulose, without extensive downstream processing—to form a composite material. The stabilized epoxy-functionalized syringolated lignin acts as a reactive bridge, forming durable ether linkages with cellulose and transforming hydrogen-bonded fiber networks into chemically crosslinked architectures. This material design was enabled by a mild, metal-free reactive fractionation that yields functional lignin and pure cellulose directly from raw biomass, including ash-rich side streams. The resulting composites demonstrate that biomass-derived polymers can rival fossil-based packaging and kraft liner produced from virgin forestry in strength, durability and moisture resistance, highlighting a scalable path toward circular, wet-tolerant bio-based materials. This study makes progress in the research field by demonstrating a holistic valorization of both lignin and cellulose streams to produce a composite with good mechanical properties without severe processing. Given the simplicity of the process and the wide tolerance to feedstocks, this study will be of interest to both the packaging industries and stakeholders that generate residual biomass streams.

## Methods

### Chemicals and materials

Poplar (*Populus trichocarpa* clone 23.4) was originally provided by the Swedish University of Agricultural Sciences, Uppsala, Sweden. Rapeseed straw was provided by Albin Gunnarson’s Fornåsa gård, south of Sweden. 2,6-Dimethoxyphenol (syringol, 99%), formic acid (≥88%), epichlorohydrin (ECH, ≥99%), sulfuric acid (95%), acetone (99.5%), ethanol (99.9%), tetrabutylammonium bromide (TBAB, 99%), sodium hydroxide (NaOH, AR), sodium chloride (NaCl), sodium sulfate (Na_2_SO_4_), barium carbonate (BaCO_3_), ethanethiol (EtSH, 97%), boron trifluoride-diethyl etherate (BF3·Et2O, 1.0 M in tetrahydrofuran), tetracosane (99.0%), bis(trimethylsilyl)trifluoroacetamide (BSTFA, 99.0%), dichloromethane (DCM, 99.4%), dimethyl sulfoxide-d_6_ (DMSO-d_6_, 99.8%), 4-dimethylaminopyridine (DMAP, ≥99%), chloroform-d (99.8%), anhydrous pyridine, Chromium (III) acetylacetonate (Cr(acac)_3_, 99.99%), N-Hydroxy-5-norbornene-2,3-dicarboxylic acid imide (NHND, 97%), 2-Chloro-4,4,5,5-tetramethyl-1,3,2-dioxaphospholane (TMDP), potassium acetate (CH₃COOK), magnesium nitrate (Mg(NO₃)₂), calcium chloride (CaCl_2_) and potassium chloride (KCl)were purchased from either VWR International or Merck. All chemicals were used as received.

### Reactive fractionation of biomass

Dry poplar biomass (5.00 g, particle size of 0.5 mm) and syringol (2.00 g, corresponding to a biomass/syringol mass ratio of 1:0.4) ﻿were treated with 88 wt% formic acid (50 mL) in a closed reactor at 120 °C for 30 min. After completion, the reaction mixture was filtered to obtain a cellulose-enriched pulp, which was sequentially washed with formic acid, ethanol and deionized water. The filtrate was concentrated by rotary evaporation and precipitated into deionized water to obtain SL. The SL was collected by centrifugation and then freeze-dried to yield a light-colored powder. The reactive fractionation procedure is readily scalable; a 30 g batch of biomass was successfully processed under identical conditions. To evaluate the influence of syringol loading, additional fractionation experiments were performed under otherwise identical conditions using biomass/syringol mass ratios of 1:0.2 and 1:0.6. For the FA-only control experiment, poplar biomass was treated under the same formic-acid fractionation conditions but without adding syringol. For the ash-mimic fractionation experiment, dry poplar biomass (2.00 g) was mixed with KCl (36 mg) and CaCl₂ (24 mg), corresponding to a total added inorganic salt loading of 3.0 wt% based on dry biomass. The biomass/syringol mass ratio was kept at 1:0.4, and all other fractionation and isolation conditions were identical to those used for the optimized poplar fractionation. The added KCl and CaCl₂ were used to mimic representative alkali and alkaline-earth metal salts present in ash-rich lignocellulosic feedstocks.

### Etherification of SL to yield an epoxy

SL (3.00 g, 9.7 mmol, 1.00 equiv., based on phenolic OH content) and tetrabutylammonium bromide (2.06 g, 6.4 mmol, 0.66 equiv.) were dissolved in 15–20 equiv. of epichlorohydrin (the exact amount adjusted according to the solubility of SL) in a 100 mL round-bottom flask equipped with a magnetic stir bar. After purging with N₂ for 5 min, the reaction mixture was stirred at 60 °C for 2 h, then immediately cooled in an ice bath. Subsequently, 4.00 equiv. of 50 wt% NaOH solution was added dropwise, and the mixture was stirred at 60 °C for another 2 h. Dichloromethane was added, and the resulting solution was washed with deionized water until neutral, followed by brine. The resulting organic phase was dried over anhydrous Na_2_SO₄, and the residual epichlorohydrin and solvent were removed via rotary evaporation. The crude product was redissolved in a small amount of acetone and precipitated in water. The resulting precipitate was collected by centrifugation and then freeze-dried to yield an off-white ESL powder. The epoxy-functionalization of SL from rapeseed straw was applied using the same method.

### Fabrication of ESL-cellulose composite

The curing process was first optimized using filter paper as a cellulose model. ESL (0.20 g) and DMAP (2 wt% of ESL) were dissolved in acetone (5 mL), in which 0.20 g of filter paper was immersed. After air drying, the impregnated filter paper was heated at 80 °C–220 °C for 1 h. The resulting ESLFP was washed with acetone under sonication for 5 min to remove non-covalently bound ESL. A control group was prepared without DMAP, named as ESLFP*. The ESL/pulp mass ratio of 1:1 was selected based on preliminary ESL–filter paper loading experiments, in which increasing the initial ESL/cellulose ratio from 1:1 to 1.5:1 or 2:1 did not appreciably increase the retained ESL content after acetone washing.

For composite fabrication, the pulp obtained from reactive fractionation and ESL (1:1) was dispersed in 100 mL of deionized water together with DMAP (2 wt%) to form a suspension. The mixture was homogenized at 10,000 rpm for 1 h and then subjected to vacuum filtration to yield a wet ESLP cake. The cake was hot-pressed at 180 °C under 3.5 MPa for 1 h to obtain the ESLP composite. For comparison, a neat pulp sheet was prepared using the same filtration and hot-pressing procedure but without adding ESL and DMAP.

### NMR analysis

^31^P NMR spectra were recorded on a Bruker AVANCE 500 spectrometer equipped with a 5-mm broadband observe (BBO) probe. The experiments were run according to a standard method reported in Nature Protocols^46^. The lignin samples were dried under vacuum at approximately 45 °C to constant weight and subsequently cooled to room temperature in a desiccator. A solvent mixture of CDCl_3_ and anhydrous pyridine (1:1.6, v/v) was used for sample preparation. The internal-standard solution contained Cr(acac)_3_ at approximately 5.0 mg mL^−1^ as a relaxation reagent and NHND at approximately 18.0 mg mL^−1^ as an internal standard.

Approximately 30 mg of dried lignin was accurately weighed and mixed with 0.10 mL of the internal-standard solution and 0.50 mL of the CDCl_3_/pyridine solvent mixture. The mixture was stirred until a homogeneous solution was obtained. Subsequently, 0.10 mL of TMDP was added, and the mixture was shaken until homogeneous. The phosphitylated solution was transferred to a 5-mm NMR tube and analyzed immediately.

Two-dimensional HSQC NMR spectra were recorded on a Bruker AVANCE 500 spectrometer equipped with a 5-mm BBO probe. Samples were dissolved in DMSO-d_6_ at a concentration of 50 mg in 0.5 mL. HSQC spectra were recorded at 298 K using Bruker’s standard hsqcedetgpsisp2.3 pulse program (acquisition times 64 ms and 6 ms in ^1^H and ^13^C dimensions, respectively; inter-scan relaxation delay 1 s). Data processing was performed using Bruker’s Topspin 4.5.0 software.

### Compositional analysis of lignin and biomass

Lignin and sugar content in biomass and pulp from reactive fractionation were quantified using a two-stage sulfuric acid hydrolysis following the National Renewable Energy Laboratory standard protocol^68^. Sugars were quantified using High Performance Liquid Chromatography on an Agilent 1200 system equipped with a refractive index detector and a 300 × 7.8 mm (L × ID) Aminex HPX–87P column operated at 85 °C using water as the eluent.

Thioacidolysis was used to determine β–O–4 content of native lignin. The reagent for thioacidolysis was prepared by mixing EtSH, BF₃·Et_2_O and dioxane in a 2.5 mL/0.7 mL/20 mL ratio and then diluting with dioxane to a 25 mL final volume. Biomass (40 mg, powder) was mixed with 4 mL of the reagent in a sealed tube. The mixture was heated at 100 °C for 4 h, then cooled in an ice bath. The reaction was quenched with saturated NaHCO_3_, and the pH was adjusted to 1–3 with 1 M HCl. The mixture was extracted sequentially with DCM, water and brine, dried over anhydrous Na_2_SO_4_, filtered and concentrated under reduced pressure. The residue was dissolved in ethyl acetate (EtOAc, 5 mL). An aliquot (0.5 mL) was combined with 0.1 mL of a tetracosane-in-EtOAc solution (10 g L^−1^, internal standard) in a 5 mL vial, and the solvent was evaporated. The dry residue was silylated with BSTFA (0.1 mL) and pyridine (0.1 mL) at 60 °C for 30 min. The derivatized sample was analyzed by GC–MS/FID.

### Isolation of ball-milled poplar lignin

Ball-milled poplar lignin was isolated from native poplar wood for HSQC NMR analysis. Poplar wood powder was first ball-milled for 24 h to disrupt the lignocellulosic structure and improve lignin extractability. The ball-milled material was then extracted with 96% aqueous dioxane for 72 h under continuous stirring. After extraction, the soluble fraction was separated from the remaining solid residue, and the solvent was removed under reduced pressure. The resulting lignin fraction was further dried to constant weight and designated as ball-milled poplar lignin.

### Mechanical characterization

Tensile properties were measured on an Instron 5960 testing system with a 1 kN load cell in accordance with ISO527-3 with slightly modification. The test was done at an operating speed of 5 mm min^−1^. The wet strength was tested by immersing samples in water for 15 min and then going for the test. The results are based on three replicates.

### Scanning electron microscope (SEM) analysis

The morphology of native biomass, fractions obtained from reactive fractionation and the corresponding composites was observed using scanning electron microscopy (SEM, JEOL JSM 7000F). Samples were sputter-coated with gold, and imaging was performed under high vacuum at an accelerating voltage of 5–20 kV.

### FTIR analysis

FTIR spectra of lignin fractions, cellulose pulp and composites were recorded using a Varian 670 spectrometer equipped with an ATR (attenuated total reflectance) accessory. Samples were analyzed in solid form without further preparation. Spectra were collected in the range of 4000–400 cm⁻¹ with a resolution of 4 cm⁻¹ and averaged over 32 scans for each measurement. The characteristic absorption bands were assigned based on literature values to identify functional groups associated with syringolation, epoxidation and covalent bonding between ESL and cellulose. In addition, a kinetic experiment was applied to ESLFP* and ESLFP. Samples reacted for 0, 10, 20, 30, 40, 50, and 60 min were analyzed using FTIR to monitor the chemical group change during the reaction.

### X-ray photoelectron spectroscopy (XPS) analysis

XPS measurements were performed using a Kratos AXIS Supra+ X-ray Photoelectron spectrometer. Samples were mounted on conductive carbon tape and analyzed under ultra-high vacuum. High-resolution spectra were recorded for the C 1*s*. All spectra were charge-corrected using the C–C/C–H component at 284.8 eV as reference. Peak fitting and deconvolution were performed using CasaXPS software. The evolution of carbon components (C1: C–C/C–H; C2: C–O; C3: C=O; C4: O–C=O) was used to evaluate chemical interactions between ESL and cellulose and to confirm epoxide ring-opening during composite formation.

### Surface wettability analysis

The surface wettability (contact angle) of ESLP and hot-pressed pulp was visualized using the KRÜSS drop shape analyzer DSA25. Water was dropped onto the surface of the samples, and the contact angle after different times was measured.

### Gel permeation chromatography (GPC) analysis

GPC analysis was performed using a Shimadzu Prominence-i LC-2030C system equipped with a UV detector operated at 280 nm. Tetrahydrofuran (THF) was used as the mobile phase at a flow rate of 0.35 mL min^−1^. Separation was performed at 35 °C using three Phenogel columns connected in series, each measuring 300 × 4.6 mm: one 5 μm, 500 Å column followed by two 5 μm, 50 Å columns. The system was calibrated using polystyrene standards, and the reported molecular weights are apparent molecular weights relative to the polystyrene calibration.

### Thermogravimetric analysis (TGA)

TGA was conducted on a Discovery TGA system (TA) to evaluate the thermal stability of native poplar, SL, ESL and ESLP. Approximately 10 mg of each dried sample was placed in a platinum pan and heated from room temperature to 800 °C at a rate of 10 °C min⁻¹ under a constant nitrogen flow to prevent oxidative degradation. Derivative thermogravimetric (DTG) curves were used to determine the temperature corresponding to the maximum rate of weight loss (*T*_max_). The progressive shift in *T*_max_ was analyzed to assess the effect of syringolation and epoxidation on the thermal stability of lignin and its derivatives.

### Long-term humidity aging and water-uptake measurement

Long-term humidity aging of ESLP was conducted in sealed desiccators containing saturated aqueous salt solutions. Saturated CH₃COOK, Mg(NO₃)₂, NaCl and KCl solutions were used to establish nominal relative humidities of approximately 25%, 55%, 75%, and 85%, respectively, at room temperature.

The specimens were then aged in the corresponding humidity chambers at room temperature for 97 days.

At predetermined intervals, the specimens were removed briefly from the chambers, weighed using an analytical balance, and immediately returned to the corresponding desiccators. At least 5 independently prepared specimens were measured under each humidity condition.

After 97 days of humidity aging, the specimens were removed from the chambers, balanced at room temperature and 50% RH for 24 h, and then subjected to tensile testing without redrying.

### Compost-burial and macroscopic disintegration test

The macroscopic disintegration behavior of ESLP under composting conditions was evaluated using a compost-burial test. The moisture content of the compost was adjusted to approximately 60% of its water-holding capacity using deionized water.

Dried ESLP specimens with an initial mass of 0.4 g were used. Filter-paper specimens of comparable dimensions were included as a cellulose-based positive control. The specimens were buried at a depth of approximately 5 cm in ventilated containers containing compost. The containers were incubated at 58 °C for 72 days under aerobic conditions.

This experiment was used to evaluate visible changes in specimen color, shape, surface integrity, erosion and fragmentation. Because residual compost could not be completely removed without damaging the specimens, the burial test was treated as a qualitative macroscopic-disintegration assessment, and no quantitative mass-loss or biodegradation percentage was calculated from this experiment.

### Compost-respiration and CO₂-evolution test

The biological degradation of ESLP under composting conditions was evaluated by monitoring CO₂ evolution. Mature compost (120 g) was placed in a modified 1000 mL glass flask equipped for headspace gas sampling. The moisture content of the compost was adjusted to 60% of its field water-holding capacity using distilled water. Dried ESLP samples (0.30 g) were cut into small pieces and mixed thoroughly with the compost, corresponding to a sample loading of 0.25 wt% relative to the compost. Compost-only flasks prepared under otherwise identical conditions were used as blanks.

All flasks were incubated at 60 °C. At each predetermined measurement interval, the flasks were sealed and statically incubated for 2 h. After incubation, a 30 mL headspace gas sample was withdrawn using a gas-tight syringe, and the CO₂ concentration was measured using an Agilent 7890 gas chromatograph. The amount of CO₂ evolved during the 2 h incubation period was calculated from the change in headspace CO₂ concentration, the available headspace volume, and the ideal gas equation using the reported method with slight modification^69^. The measured 2 h CO₂ evolution was subsequently extrapolated to a long-term equivalent value.

The net CO₂ evolution associated with ESLP addition was calculated by subtracting the CO₂ evolution of the compost-only blank from that of the compost containing ESLP:

$${{{\rm{Net}}}}\,{{{\rm{C}}}}{{{{\rm{O}}}}}_{2}{{{\rm{evolution}}}}={{{\rm{C}}}}{{{{\rm{O}}}}}_{2}{{{\rm{from}}}}\,{{{\rm{compost}}}}\,{{{\rm{with}}}}\,{{{\rm{ESLP}}}}-{{{\rm{C}}}}{{{{\rm{O}}}}}_{2}{{{\rm{from}}}}\,{{{\rm{compost}}}}\,{{{\rm{blank}}}}$$

Detailed calculations and fitting procedures are provided in the Supplementary Information.

## Supplementary information


Supplementary Information
Transparent Peer Review File


## Source data


Source Data


## Data Availability

The data generated in this study are provided within the Article, Supplementary Information and Source Data file. The source data underlying the graphs and charts are provided in the Source Data file. No custom code was used in this study. All data are available from the corresponding author upon request. Source data are provided with this paper.
