# Supplementary material for: Composites of covalently linked lignin and cellulose from one feedstock
Source: Nat Commun. 2026 Aug 27;17:9145. doi: 10.1038/s41467-026-77206-8 (PMC13522584; doi:10.1038/s41467-026-77206-8)
Supplement: Supplementary file 1 [file 41467_2026_77206_MOESM1_ESM.pdf]

Composites of covalently linked lignin and cellulose from one feedstock

Shida Zuo[1,2], Lars William Schick[1], Suthawan Muangmeesri[1], Saranya Chitsomkhuan[1], Lenny Haddad[1], Joseph S. M. Samec[1,2,*].

[1] Department of Chemistry, Stockholm University, Stockholm 114 18, Sweden

[2] Wallenberg Initiative Materials Science for Sustainability, Department of Chemistry, Stockholm University, Stockholm 114 18, Sweden

[*] Corresponding author email: joseph.samec@su.se

Supplementary Information

Contents

Composites of covalently linked lignin and cellulose from one feedstock 1

Supplementary characterizations 5

Calculation of SL yield 6

Supplementary texts 7

ST. 1: Mechanical properties of ESLP prepared using rapeseed straw 7

ST. 2: XRD analysis of ash from rapeseed straw 7

ST. 3: Ash mimic fractionation experiment 7

Supplementary figures 8

Fig. S1: ^1H–^{13}C HSQC NMR spectrum of native ball milled lignin of poplar in DMSO-d_6. The spectrum shows typical hardwood lignin correlations, including syringyl and guaiacyl aromatic signals and β–O–4 interunit-linkage signals. The native β–O–4 Cα–Hα correlation provides a structural reference for comparison with syringolated lignin. 8

Fig. S2: ^1H–^{13}C HSQC NMR spectrum of SL in DMSO-d_6 (biomass/syringol ratio = 1:0.2). Syringolated lignin (SL). 9

Fig. S3: ^1H–^{13}C HSQC NMR spectrum of SL in DMSO-d_6 (biomass/syringol ratio = 1:0.4). Syringolated lignin (SL). 10

Fig. S4: ^1H–^{13}C HSQC NMR spectrum of SL in DMSO-d_6 (biomass/syringol ratio = 1:0.6). Syringolated lignin (SL). 11

Fig. S5: ^1H–^{13}C HSQC NMR comparison of FA-only fractionated lignin in DMSO-d_6. FA-only lignin was obtained from formic-acid fractionation in the absence of syringol. Compared with SL, FA-only lignin did not show the diagnostic Cα-syringolated correlations and exhibited negligible β–O–4 correlations, indicating that syringol is required to selectively trap the reactive Cα intermediate and suppress non-selective acid-induced lignin condensation. 12

Fig. S6: Photographs of ESLFP and ESLFP*. a before and **b** after acetone washing. Epoxy-functionalized syringolated lignin (ESL), ESL-filter paper composite (ESLFP), ESL-filter paper composite without catalyst (ESLFP*). 12

Fig. S7: FTIR analysis of ESL, FP, ESLFP* and ESLFP. Epoxy-functionalized syringolated lignin (ESL), filter paper (FP), ESL-filter paper composite (ESLFP), ESL-filter paper composite without catalyst (ESLFP*). 13

Fig. S8: Photographs of SL and ESL produced from rapeseed straw. Syringolated lignin (SL), epoxy-functionalized syringolated lignin (ESL). 13

Fig. S9: ^1H–^{13}C HSQC NMR spectrum of SL in DMSO-d_6 (rapeseed straw). Syringolated lignin (SL). 14

Fig. S10: ^1H–^{13}C HSQC NMR spectrum of ESL in DMSO-d_6 (rapeseed straw). Epoxy-functionalized syringolated lignin (ESL). 15

Fig. S11: Mechanical properties of ESLP from rapeseed straw. a Tensile stress-strain curves of ESLP and pulp of rapeseed straw. b Tensile strength and c modulus of ESLP and pulp of rapeseed straw. d Wet tensile stress-strain curves of ESLP and pulp of rapeseed straw. e Wet tensile strength and f modulus of ESLP and pulp of rapeseed straw. The error bars represent the standard deviation of data from three replicate measurements. Epoxy-functionalized syringolated lignin (ESL), ESL-pulp composite (ESLP). 16

Fig. S12: XRD analysis of cellulose crystallinity and rapeseed straw ash. a XRD pattern of rapeseed straw ash. The major diffraction features are tentatively assigned to SiO_2-related phases, K-related salts, Ca-related phases and overlapping K/Ca-containing inorganic phases. **b** XRD patterns of native poplar, fractionated pulp and ESLP. Epoxy-functionalized syringolated lignin (ESL), ESL-pulp composite (ESLP). 16

Fig. S13: Water uptake of ESLP during long-term humidity aging. Water uptake of ESLP exposed to saturated potassium acetate, magnesium nitrate, sodium chloride and potassium chloride solutions, corresponding to nominal relative humidities of approximately 25%, 55%, 75% and 85%, respectively, at room temperature. The error bars represent the standard deviation of data from five replicate measurements. Epoxy-functionalized syringolated lignin (ESL), ESL-pulp composite (ESLP). 17

Fig. S14: Cumulative CO_2 evolution from ESLP during compost incubation. Cumulative net CO_2 evolution during 65 days of composting after subtraction of the compost blank. The symbols represent the experimental data, and the line represents an empirical Boltzmann sigmoidal function fit. Epoxy-functionalized syringolated lignin (ESL), ESL-pulp composite (ESLP). 18

Fig. S15: a TG and b DTG curves of ESLP, represent the thermal stability of sample. The main DTG maximum occurs at 384.8 °C. Epoxy-functionalized syringolated lignin (ESL), ESL-pulp composite (ESLP). 18

Supplementary tables 19

Table S1. Compositional analysis of poplar and rapeseed straw. 19

Table S2. Determination of β–O–4 content and S/G ratio in lignins of poplar and rapeseed straw. 19

Table S3. Optimization of syringol assisted reactive fractionations. 19

Table S4. GPC-derived molecular-weight characteristics of the sharp high-molecular-weight fraction and broad lower-molecular-weight fraction. 19

Table S5. Apparent kinetic parameters for epoxy consumption during the ESL–cellulose reaction. 20

Table S6. Comparison between the present reactive fractionation strategy and representative lignin-first/RCF strategies[7–12]. 20

Table S7. Comparison of ESLP with representative bio-based and conventional packaging materials. 21

Appendixes 23

^1H–^{13}C HSQC NMR spectrum of ESL (poplar). HSQC NMR spectrum of ESL in DMSO-d_6, showing syringyl aromatic correlations and aliphatic correlations assigned to epoxy methylene/methine groups. Epoxy-functionalized syringolated lignin (ESL). 23

^{1}H–^{13}C HSQC NMR spectrum of SL from ash mimic experiment. HSQC NMR spectrum of SL in DMSO-d_6, showing syringyl aromatic correlations. Syringolated lignin (SL). 24

Kinetic experiments of ESLFP prepared under a 140 °C and b 160 °C. Epoxy-functionalized syringolated lignin (ESL), ESL-filter paper composite (ESLFP). 24

Supplementary References 25

Supplementary characterizations

Kinetic analysis of the ESL–cellulose reaction

The consumption of epoxy groups during the reaction between ESL and cellulose was monitored by time-resolved FTIR spectroscopy. ESLFP samples were cured at 140, 160 and 180 °C for 0, 10, 20, 30, 40, 50 and 60 min.

To minimize differences arising from sample thickness and contact with the ATR crystal, each FTIR spectrum was first normalized using the band at approximately 1495 cm^{-1} as an internal reference. This reference band remained essentially unchanged during curing. After normalization, a local baseline was applied to the epoxy-band region. Identical normalization, baseline-correction and integration parameters were applied to all spectra.

The epoxy-related band centered at approximately 908 cm^{-1} was integrated using identical baseline and integration parameters for all spectra. The integrated epoxy-band area at each reaction time, A_t, was normalized against the initial area at 0 min, A_0. The apparent rate constants were determined using an apparent pseudo-first-order model:

$$\ln\left(\frac{A_0}{A_t}\right) = k_{\mathrm{app}}t \quad (1)$$

where k_{app} is the apparent rate constant and t is the reaction time. Linear fitting of $\ln(A_0/A_t)$ against reaction time was performed with the intercept constrained to zero. The apparent activation energy, $E_{\mathrm{a,app}}$, was calculated from the Arrhenius relationship by fitting $\ln(k_{\mathrm{app}})$ against $1/T$. This FTIR-based kinetic treatment followed established approaches for monitoring epoxy-group consumption during curing[1].

Because the reaction occurs at a heterogeneous ESL–cellulose interface and the FTIR peak area provides an indirect measure of the remaining epoxy functionality, the calculated rate constants and activation energy are reported as apparent kinetic parameters. The resulting kinetic parameters are summarized in Table S5.

The data processing was using Spectragryph - optical spectroscopy software, version 1.2.16.1, 2022, from F. Menges.

X-ray diffraction analysis

X-ray diffraction (XRD) analysis was performed using a PANalytical X’Pert PRO diffractometer equipped with a Cu anode operated at 45 kV and 40 mA. Measurements were conducted in reflection mode using Cu Kα radiation (Kα1 = 1.54060 Å, Kα2 = 1.54443 Å) at room temperature. Samples were measured using a reflection–transmission spinner stage to improve sample averaging.

For native poplar, fractionated pulp and ESLP, XRD patterns were collected over a 2θ range of 10–50°. The relative crystallinity index (CrI) was calculated using the Segal method:

$$\mathrm{CrI} = \frac{I_{002} - I_{\mathrm{am}}}{I_{002}} \times 100\% \quad (2)$$

where I_{002} is the intensity of the cellulose I crystalline peak at approximately 22.5° and Iam is the intensity of the amorphous contribution at approximately 18.0°.

For ash analysis, the rapeseed straw was ashed in a muffle furnace at 575 °C. The ash residue was analyzed directly as a powder using the same diffractometer setup. The ash sample was scanned over a 2θ range of 10–80°.

Compost-respiration and CO_2-evolution test

The cumulative net CO_2-evolution data were fitted by nonlinear least-squares regression using the following empirical Boltzmann sigmoidal function:

$$y = A_2 + \frac{A_1 - A_2}{1 + \exp\left(\frac{x - x_0}{\Delta x}\right)} \tag{3}$$

where y is the cumulative net CO_2 evolution (mg), x is the composting time (days), A_1 and A_2 are the lower and upper asymptotic values, respectively, x_0 is the midpoint of the sigmoidal transition, and Δx is the transition-width parameter. The fitted parameters were A_1 = −53.61 ± 57.54 mg, A_2 = 996.96 ± 58.95 mg, x_0 = 30.64 ± 1.65 days and Δx = 13.52 ± 2.32 days. Parameter uncertainties represent the standard errors obtained from the nonlinear fit. The fit gave R^2 = 0.99331, an adjusted R^2 = 0.99177, and a reduced chi-square value of 836.55. The fitted function was used only to describe the overall CO_2-evolution profile, and the fitted parameters were not interpreted as intrinsic biodegradation kinetic constants.

Calculation of SL yield

The theoretical maximum yield of SL was estimated as follows:

The β-O-4 content of native poplar lignin is 47.1% from thioacidolysis and the S/G ratio was 1.90. The Klason lignin content of poplar was 28.1%. Therefore, the average molecular weight (MW) of native lignin unit is:

$$\mathrm{MW_L} = \frac{226 \times 1.9 + 196.0 \times 1.0}{2.9} = 215.7\ \mathrm{g\ mol^{-1}} \tag{4}$$

After syringolation, the average MW is:

$$\mathrm{MW_{SL'}} = \mathrm{MW_L} + 136.0 = 351.7\ \mathrm{g\ mol^{-1}} \tag{5}$$

The formylation of SL was reported as 90%, therefore, the average MW of SL is:

$$\mathrm{MW_{SL}} = (MW_{SL'} + 28.0) \times 0.9 + MW_{SL'} = 376.9\ \mathrm{g\ mol^{-1}} \tag{6}$$

The theoretical maximum yield is:

$$Y_{SL-max} = \frac{Lignin\ content \times 0.471 \times MW_{SL}}{MW_L} + Lignin\ content \times (1 - 0.471) = 38.0\% \tag{7}$$

The $Y_{SL\text{-}max}$ of rapeseed straw is calculated to 26.3% according to the same process above.

Supplementary texts

ST. 1: Mechanical properties of ESLP prepared using rapeseed straw

Guided by the filter-paper model, the same fractionation and epoxy-functionalization sequence translated to rapeseed straw, an herbaceous residue typically richer in ash, affording a brownish SL and a paler off-white solid ESL after precipitation (Fig. S8). HSQC spectra showed the expected Cα–arylation correlations for SL and the additional aliphatic cross-peaks attributable to the epoxide motif for ESL, mirroring the poplar case (Fig. S9–10). Despite this compositional challenge, the DMAP-assisted epoxide–cellulose coupling remained effective, yielding substantial improvements in both dry and wet properties (Fig. S11): ESLP reached 40.0 MPa tensile strength and 3.0 GPa modulus versus 11.9 MPa and 1.1 GPa for neat straw pulp; under wet conditions, ESLP retained 21.8 MPa tensile strength and 1.8 GPa modulus compared to 1.5 MPa and 0.1 GPa for neat pulp. These results demonstrate that the chemistry is robust to feedstock variability and tolerant to elevated ash content, supporting co-valorization of lignin and cellulose from different biomass streams.

ST. 2: XRD analysis of ash from rapeseed straw

To better understand the inorganic components responsible for the ash content, XRD analysis was performed on the ash residue from rapeseed straw (Fig. S12a). The pattern showed crystalline diffraction features that were tentatively assigned to SiO_2-related phases, K-containing salts, Ca-containing mineral phases and overlapping K/Ca-related inorganic phases, consistent with reported mineral phases in herbaceous biomass ashes[3–6]. Because biomass ash is a complex multiphase residue, these assignments are used to identify representative inorganic components rather than definitive single crystalline phases for every peak.

ST. 3: Ash mimic fractionation experiment

An ash-mimic fractionation experiment was performed by adding representative K and Ca salts (3 wt% of biomass) to poplar. The ash-mimic system gave a comparable SL yield of 35.5% and pulp purity of 96.7% (Table S3), close to the optimized salt-free condition, indicating that these inorganic salts did not substantially inhibit syringol-assisted fractionation under the tested conditions.

Supplementary figures

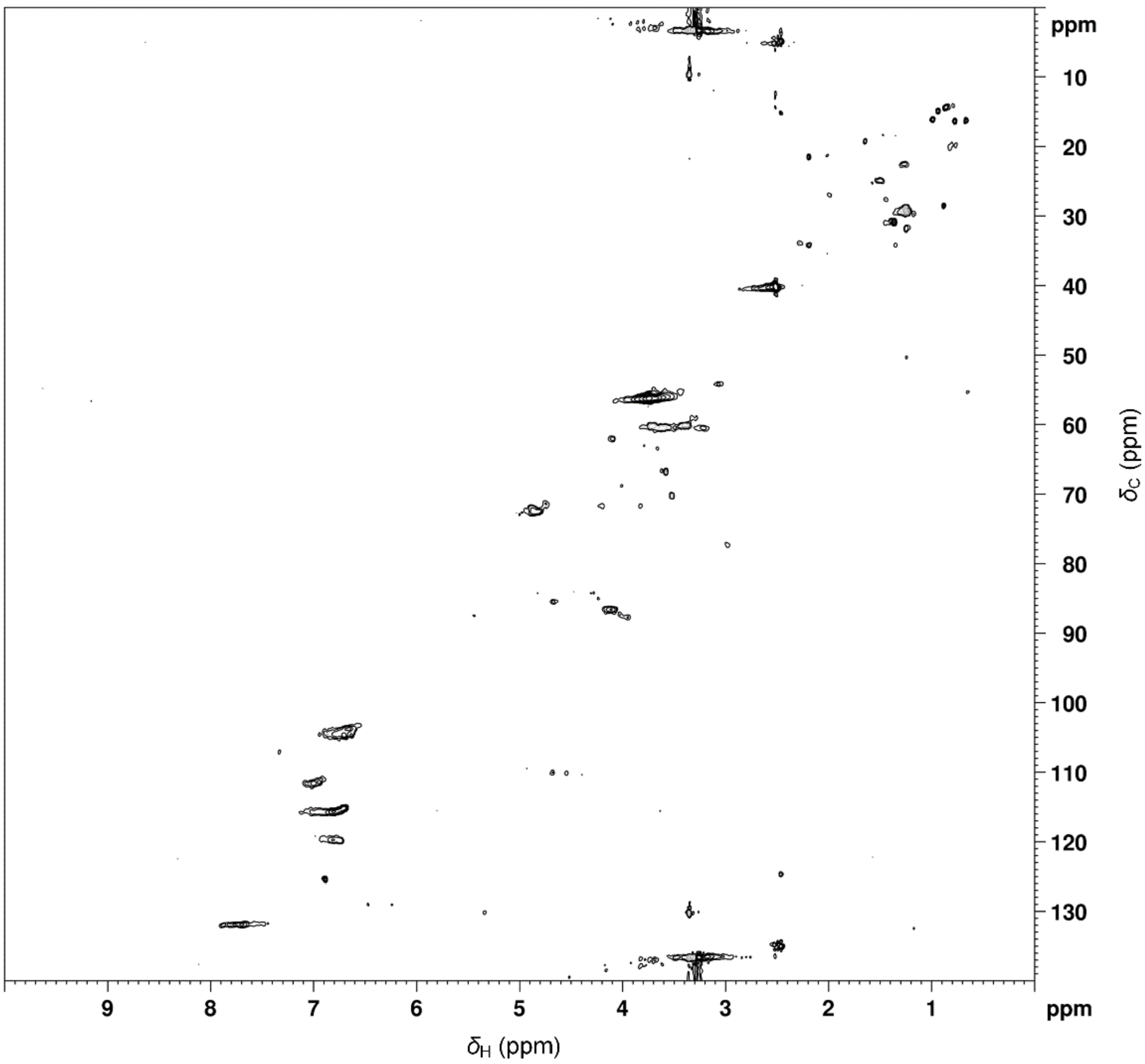

Fig. S1: ^{1}H–^{13}C HSQC NMR spectrum of native ball milled lignin of poplar in DMSO-d$_6$. The spectrum shows typical hardwood lignin correlations, including syringyl and guaiacyl aromatic signals and β–O–4 interunit-linkage signals. The native β–O–4 Cα–Hα correlation provides a structural reference for comparison with syringolated lignin.

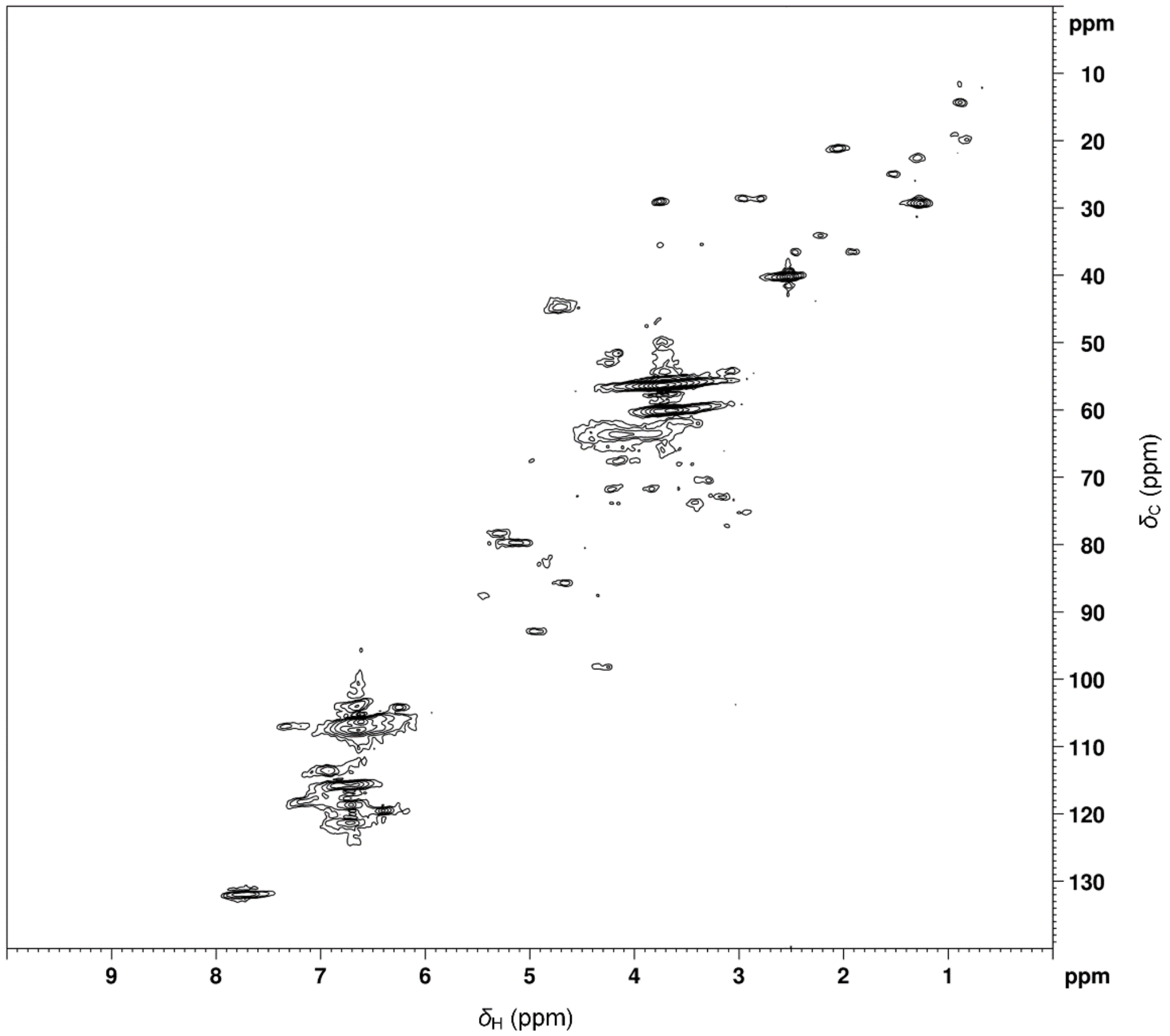

Fig. S2: ^{1}H–^{13}C HSQC NMR spectrum of SL in DMSO-d_6 (biomass/syringol ratio = 1:0.2). Syringolated lignin (SL).

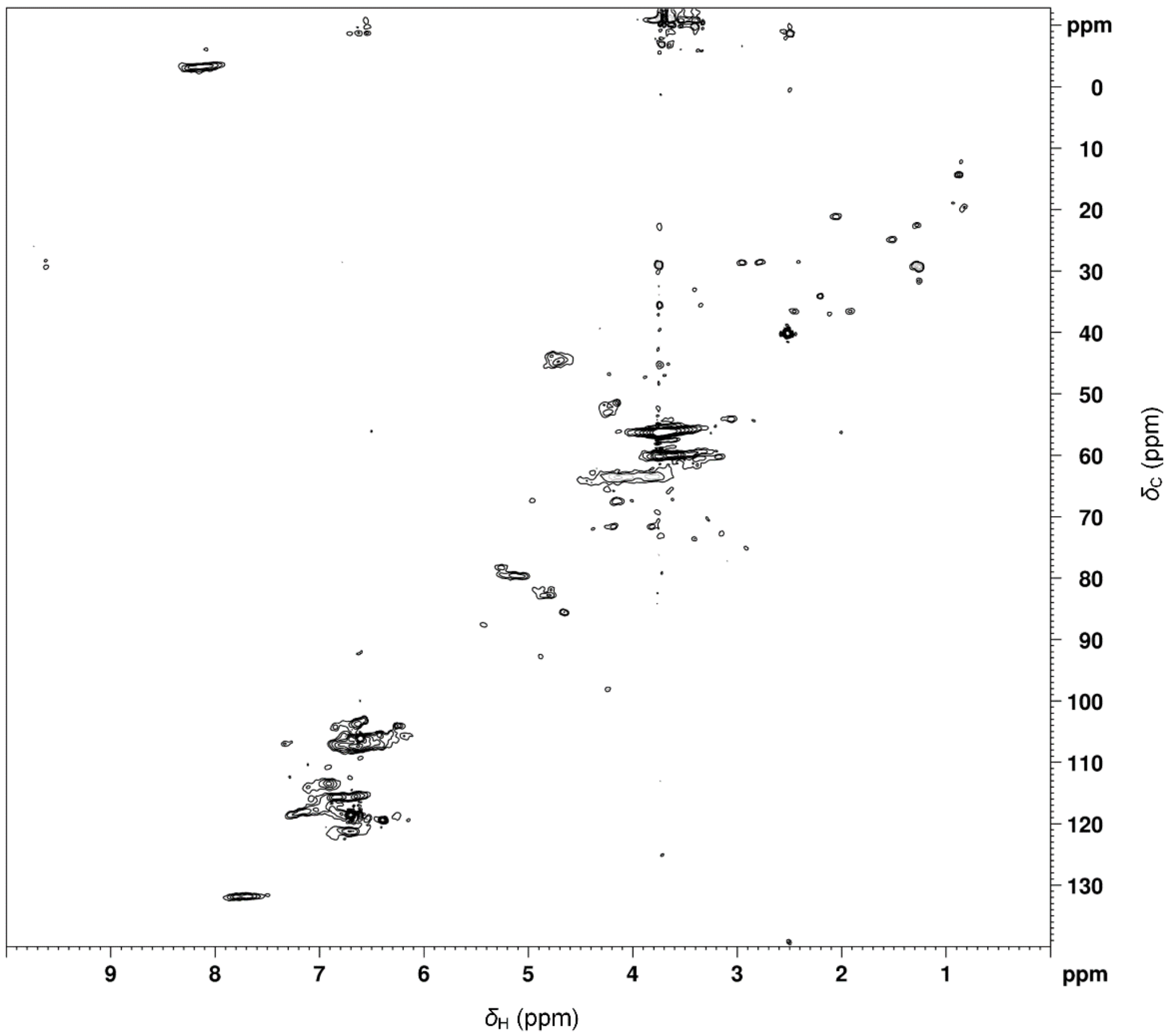

Fig. S3: ^{1}H–^{13}C HSQC NMR spectrum of SL in DMSO-d$_6$ (biomass/syringol ratio = 1:0.4). Syringolated lignin (SL).

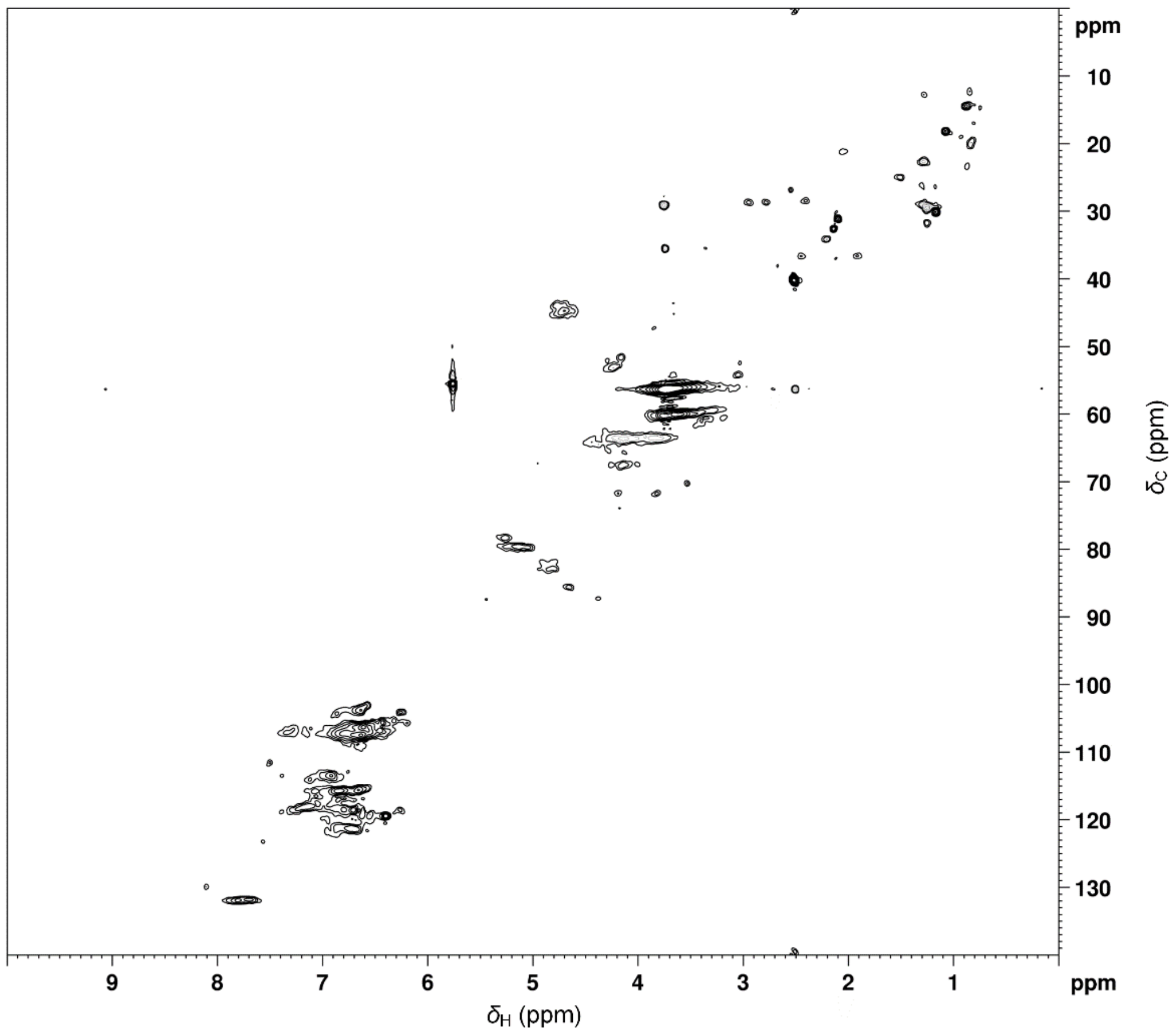

Fig. S4: ^{1}H–^{13}C HSQC NMR spectrum of SL in DMSO-d_6 (biomass/syringol ratio = 1:0.6). Syringolated lignin (SL).

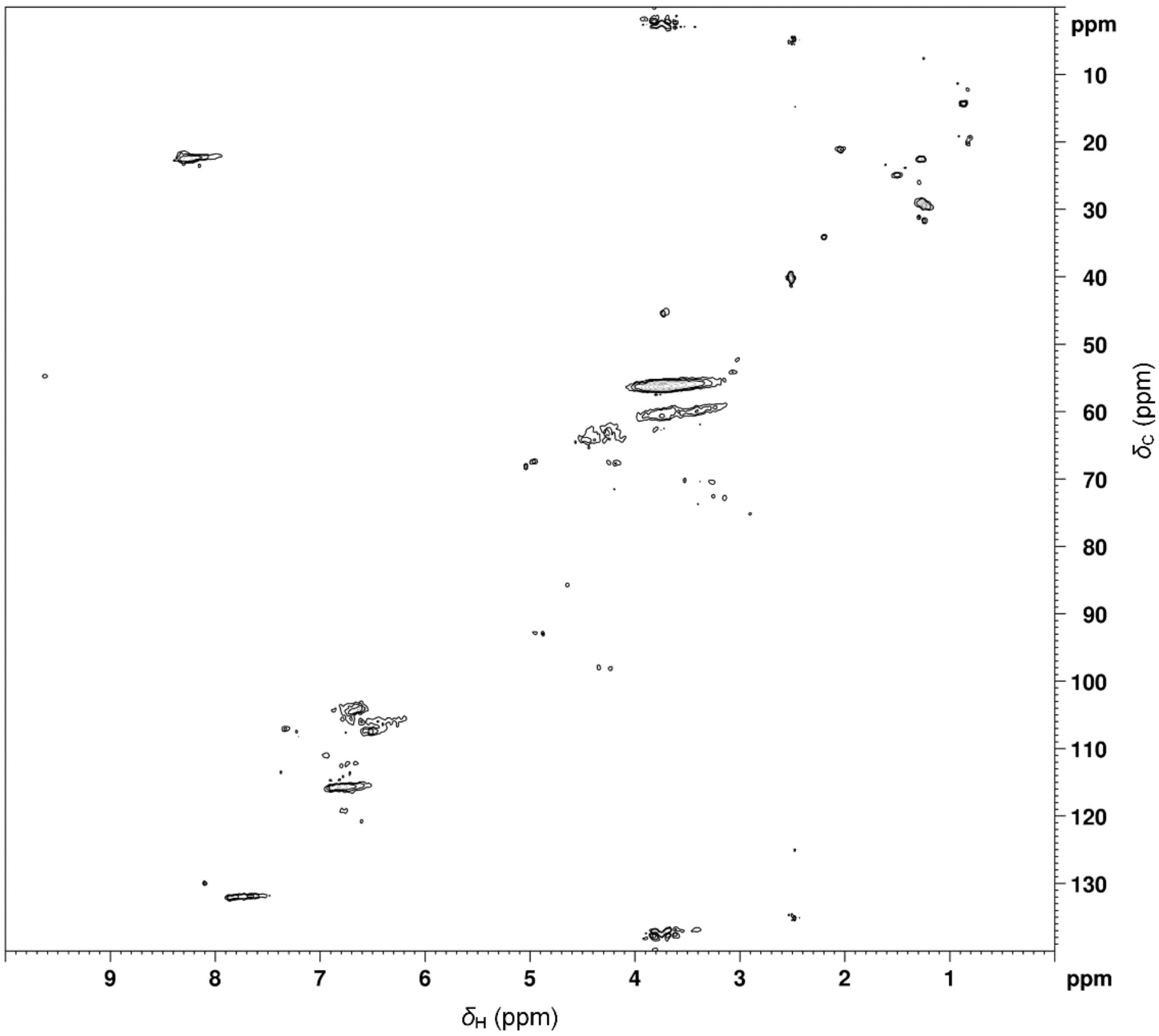

Fig. S5: ^{1}H–^{13}C HSQC NMR comparison of FA-only fractionated lignin in DMSO-d$_6$. FA-only lignin was obtained from formic-acid fractionation in the absence of syringol. Compared with SL, FA-only lignin did not show the diagnostic Cα-syringolated correlations and exhibited negligible β–O–4 correlations, indicating that syringol is required to selectively trap the reactive Cα intermediate and suppress non-selective acid-induced lignin condensation.

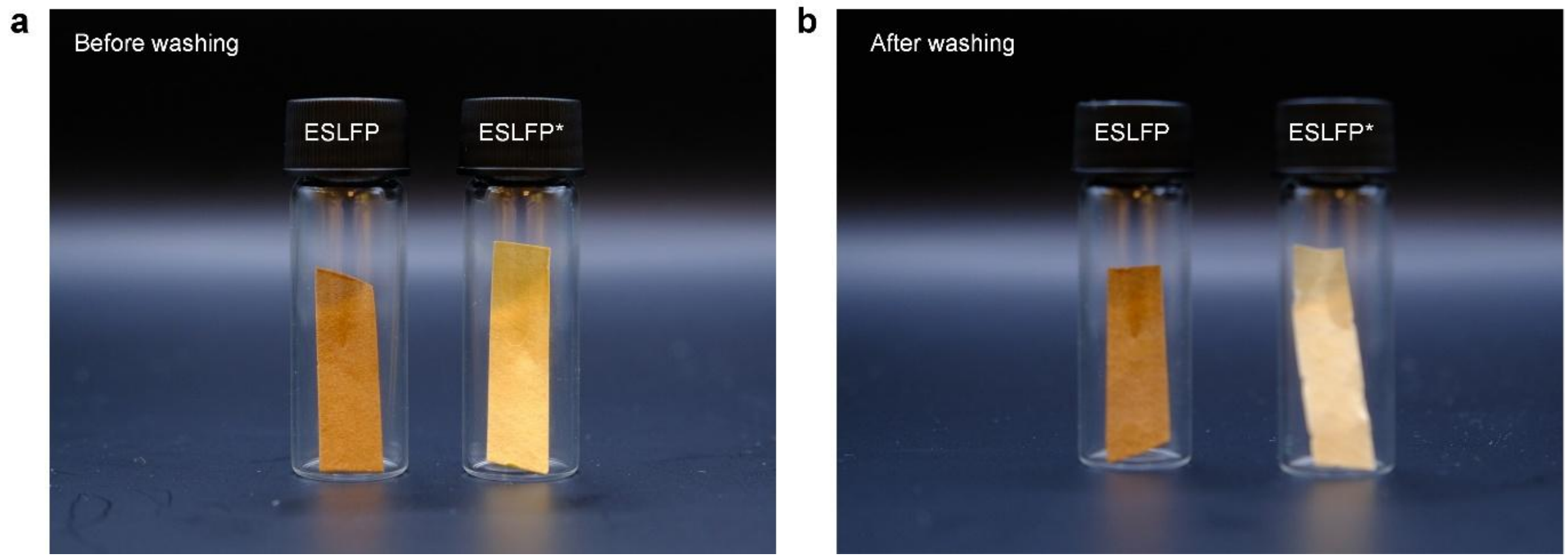

Fig. S6: Photographs of ESLFP and ESLFP*. a before and **b** after acetone washing. Epoxy-functionalized syringolated lignin (ESL), ESL-filter paper composite (ESLFP), ESL-filter paper composite without catalyst (ESLFP*).

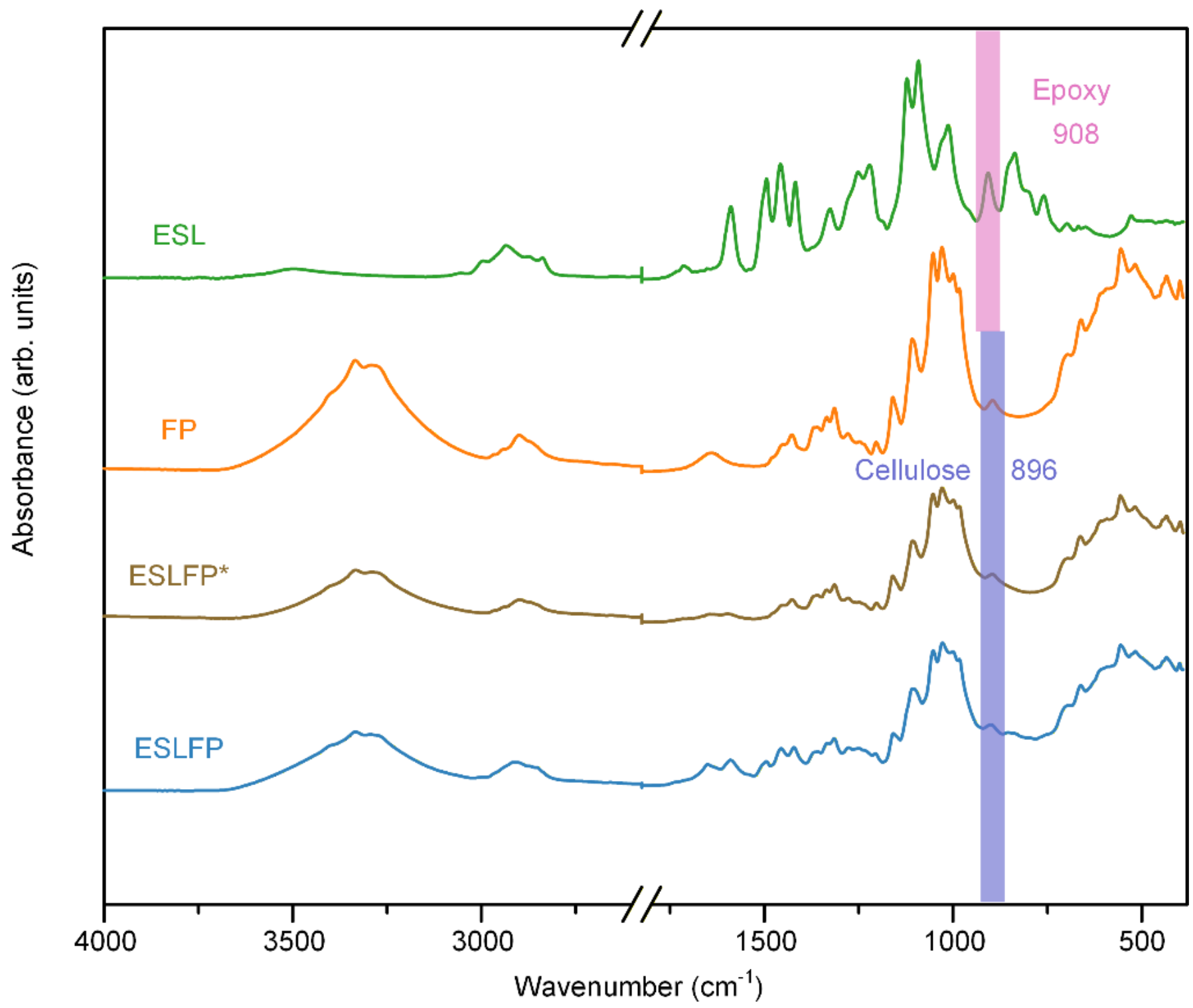

Fig. S7: FTIR analysis of ESL, FP, ESLFP* and ESLFP. Epoxy-functionalized syringolated lignin (ESL), filter paper (FP), ESL-filter paper composite (ESLFP), ESL-filter paper composite without catalyst (ESLFP*).

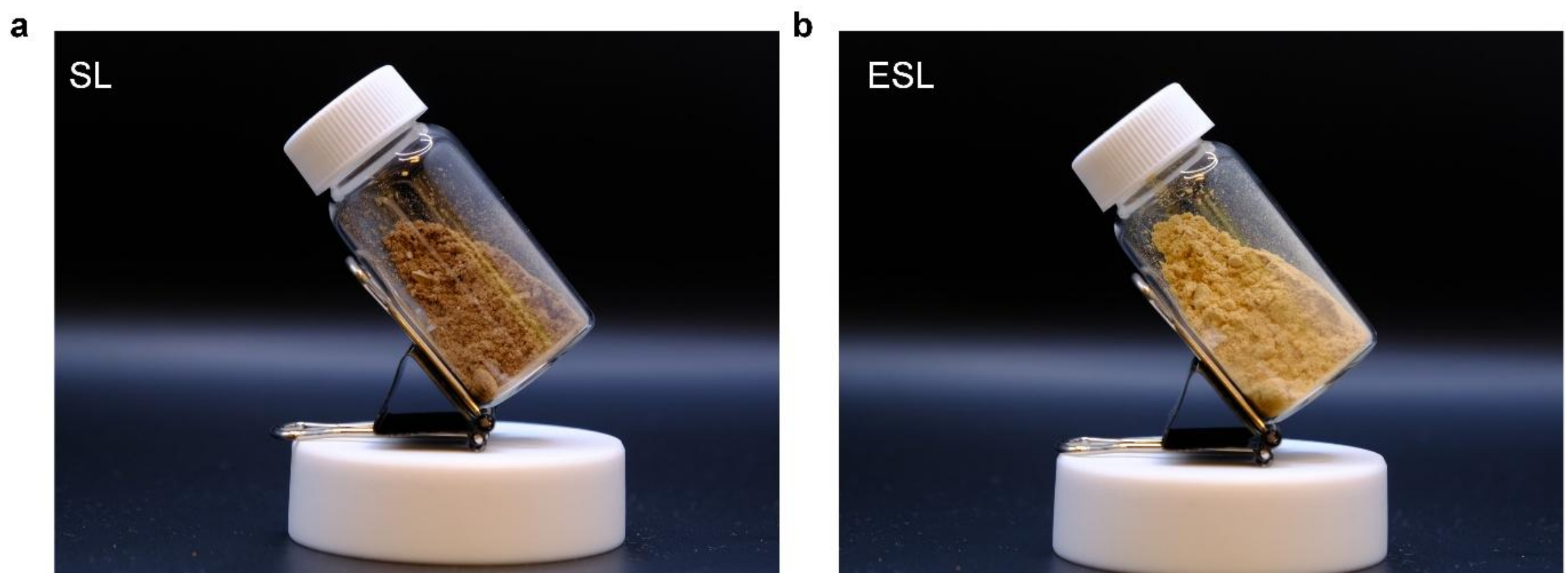

Fig. S8: Photographs of SL and ESL produced from rapeseed straw. Syringolated lignin (SL), epoxy-functionalized syringolated lignin (ESL).

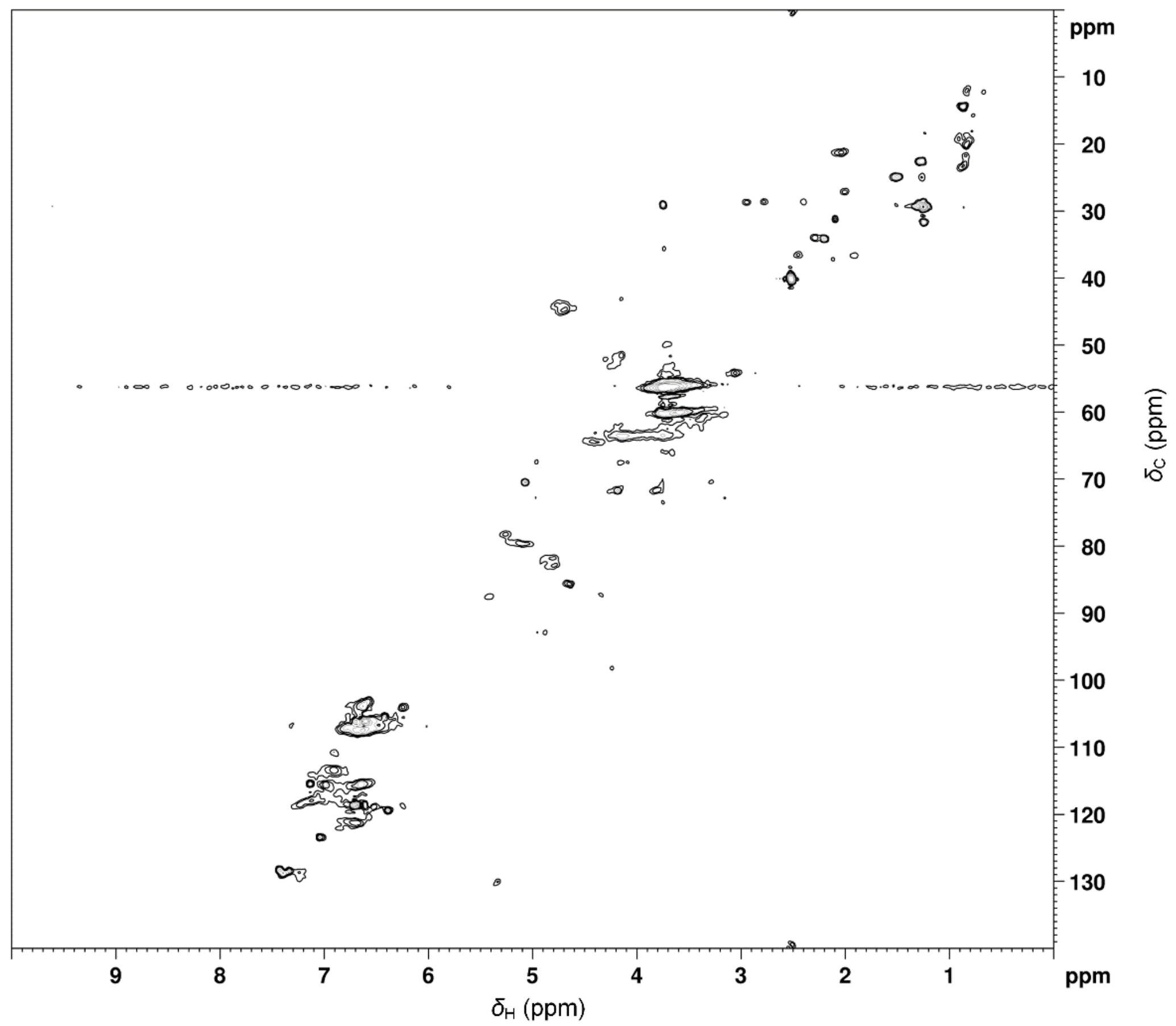

Fig. S9: ^{1}H–^{13}C HSQC NMR spectrum of SL in DMSO-d_6 (rapeseed straw). Syringolated lignin (SL).

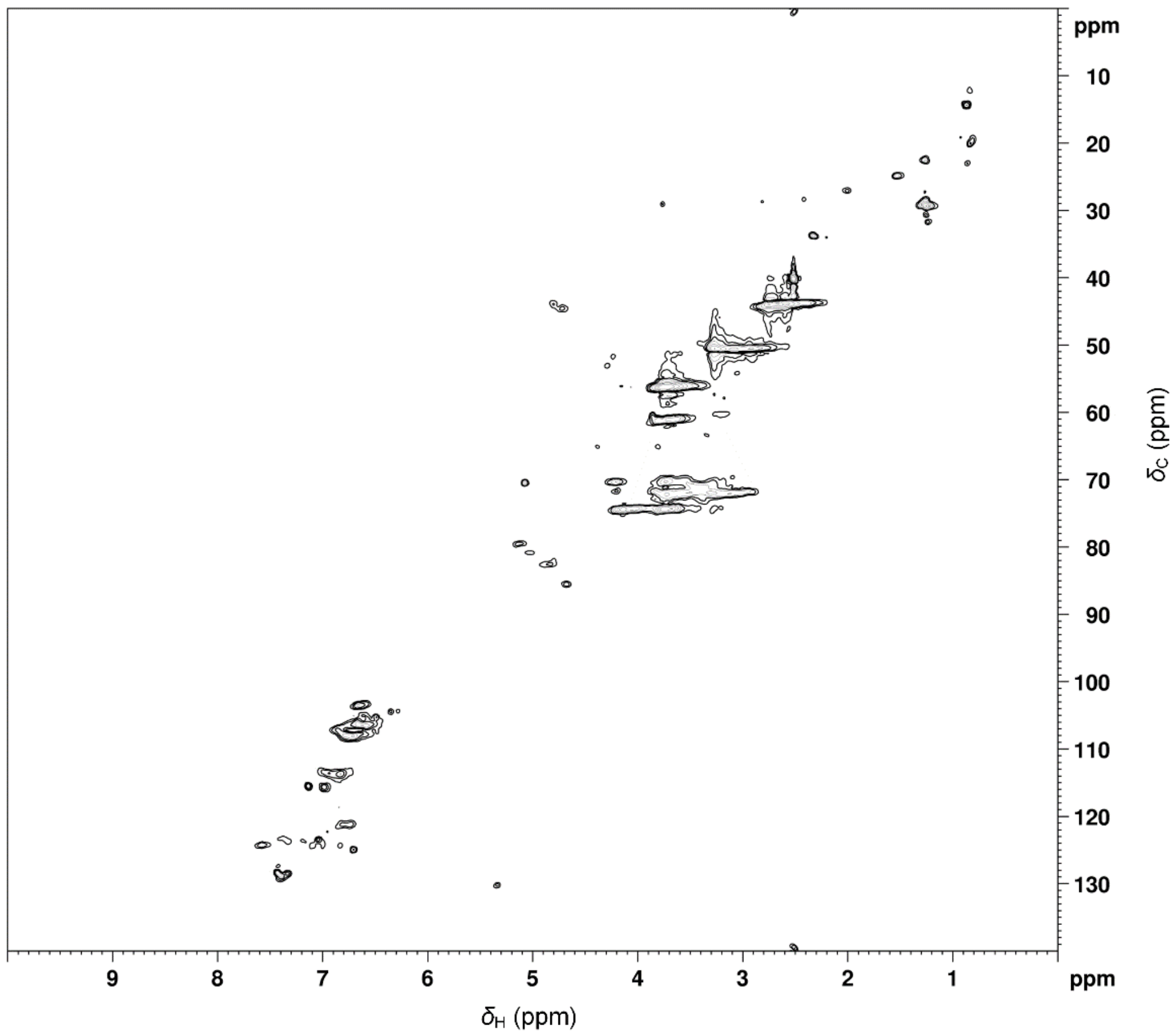

Fig. S10: ^{1}H–^{13}C HSQC NMR spectrum of ESL in DMSO-d$_6$ (rapeseed straw). Epoxy-functionalized syringolated lignin (ESL).

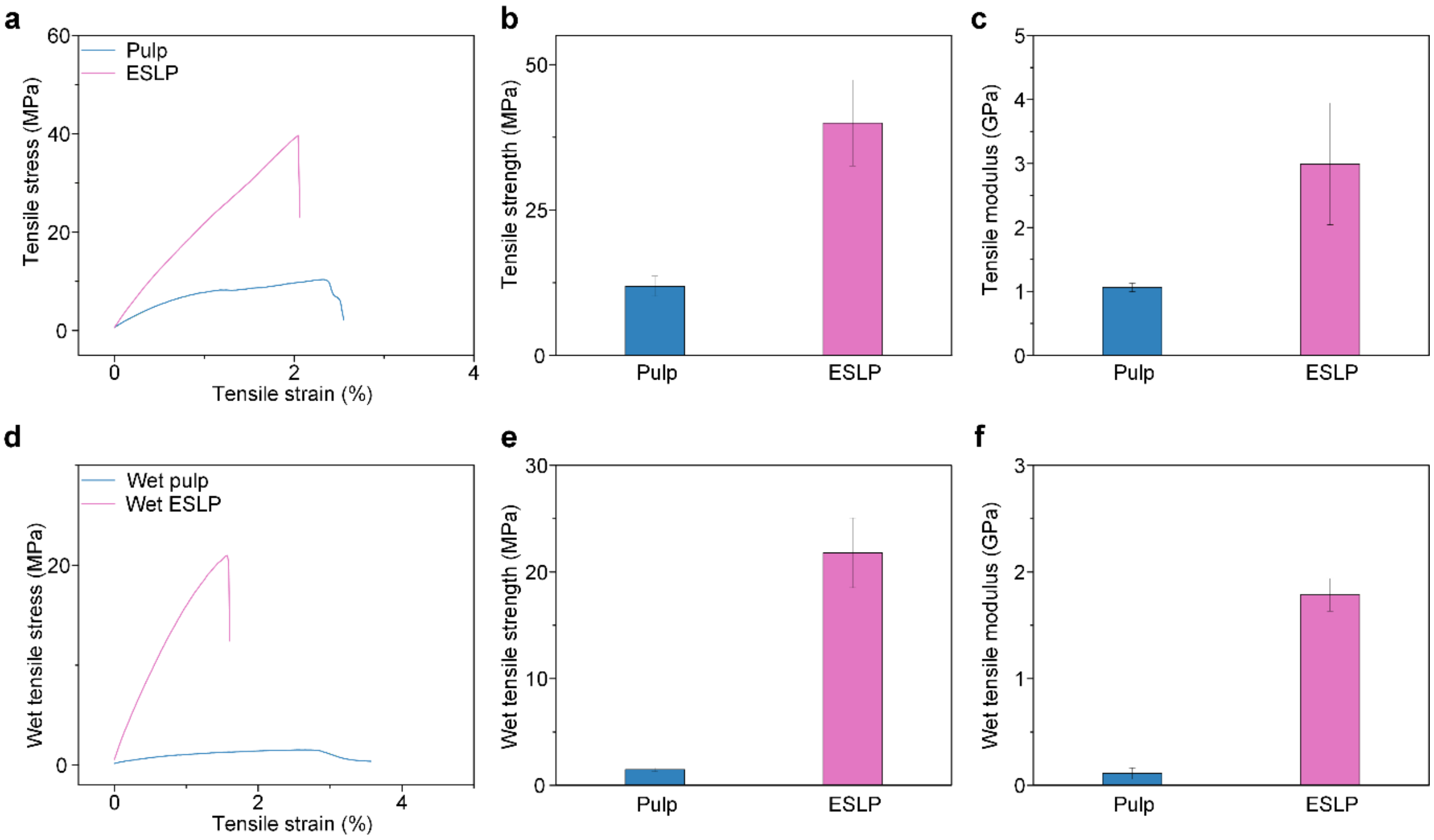

Fig. S11: Mechanical properties of ESLP from rapeseed straw. a Tensile stress-strain curves of ESLP and pulp of rapeseed straw. b Tensile strength and c modulus of ESLP and pulp of rapeseed straw. d Wet tensile stress-strain curves of ESLP and pulp of rapeseed straw. e Wet tensile strength and f modulus of ESLP and pulp of rapeseed straw. The error bars represent the standard deviation of data from three replicate measurements. Epoxy-functionalized syringolated lignin (ESL), ESL-pulp composite (ESLP).

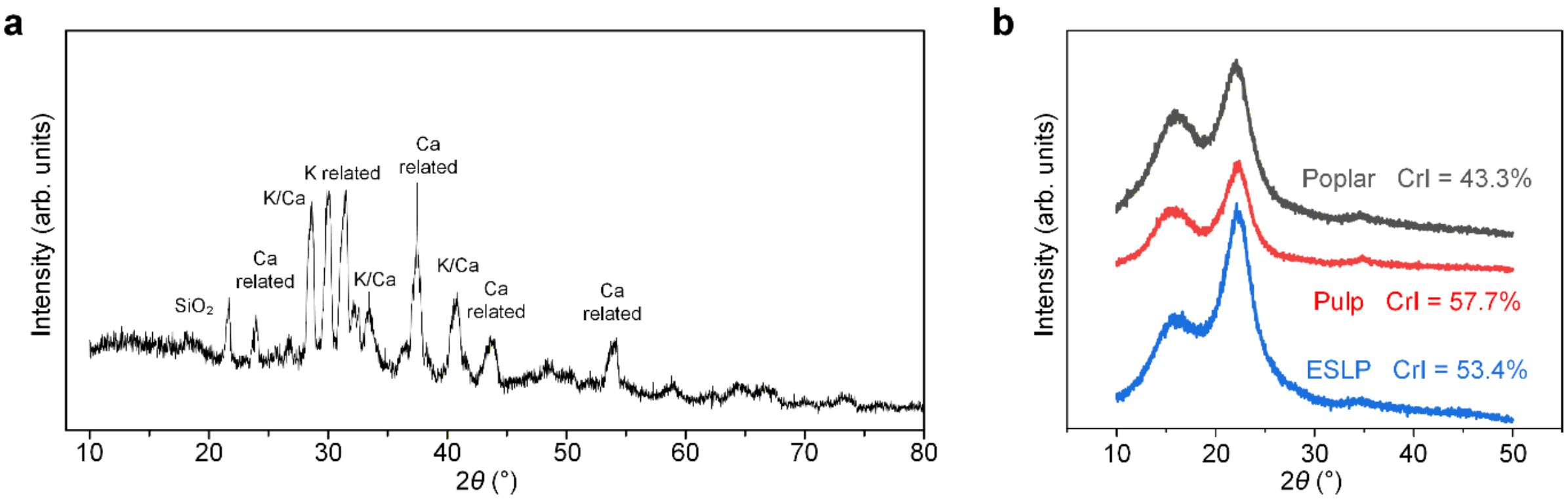

Fig. S12: XRD analysis of cellulose crystallinity and rapeseed straw ash. a XRD pattern of rapeseed straw ash. The major diffraction features are tentatively assigned to SiO_2-related phases, K-related salts, Ca-related phases and overlapping K/Ca-containing inorganic phases. **b** XRD patterns of native poplar, fractionated pulp and ESLP. Epoxy-functionalized syringolated lignin (ESL), ESL-pulp composite (ESLP).

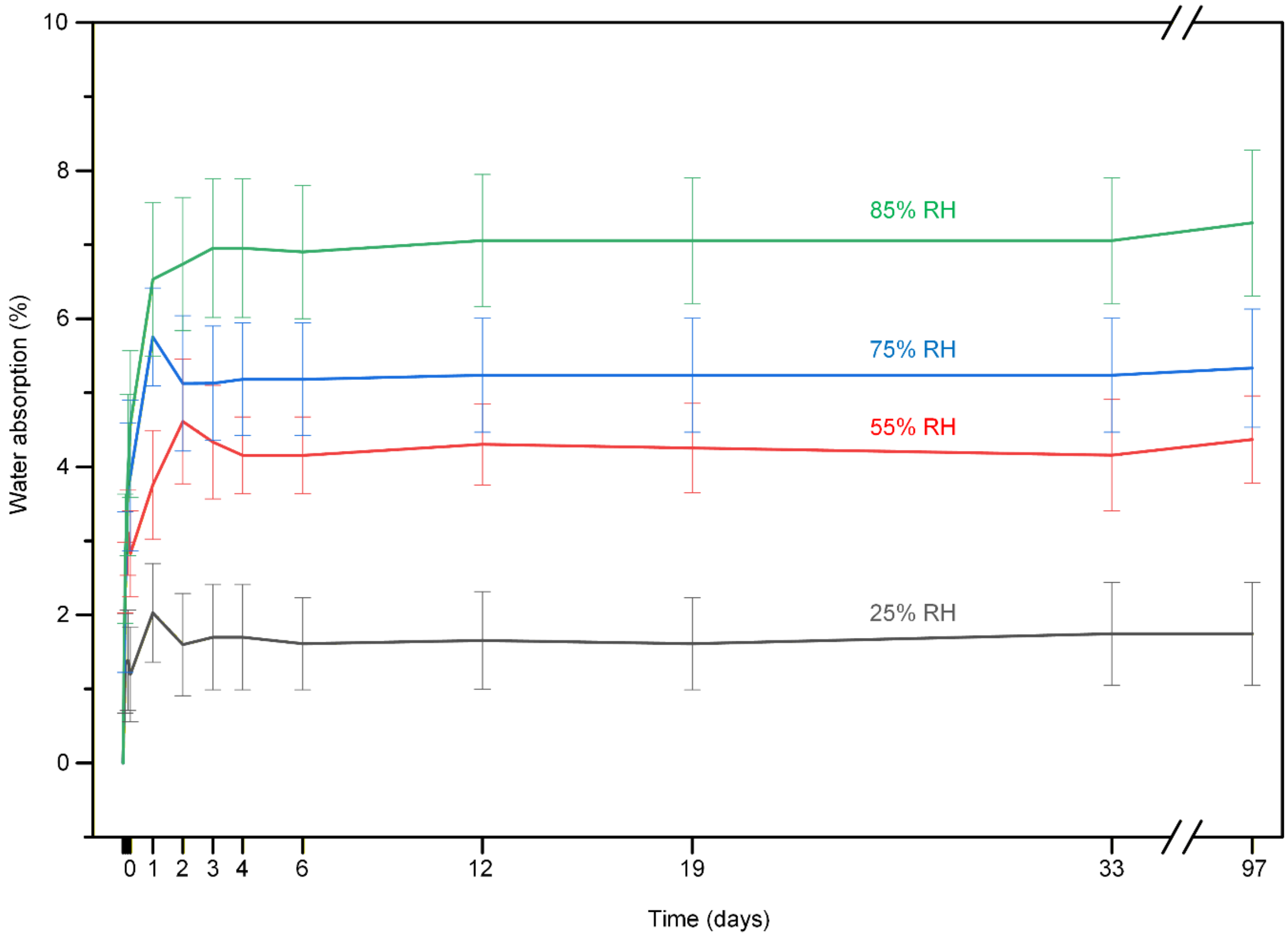

Fig. S13: Water uptake of ESLP during long-term humidity aging. Water uptake of ESLP exposed to saturated potassium acetate, magnesium nitrate, sodium chloride and potassium chloride solutions, corresponding to nominal relative humidities of approximately 25%, 55%, 75% and 85%, respectively, at room temperature. The error bars represent the standard deviation of data from five replicate measurements. Epoxy-functionalized syringolated lignin (ESL), ESL-pulp composite (ESLP).

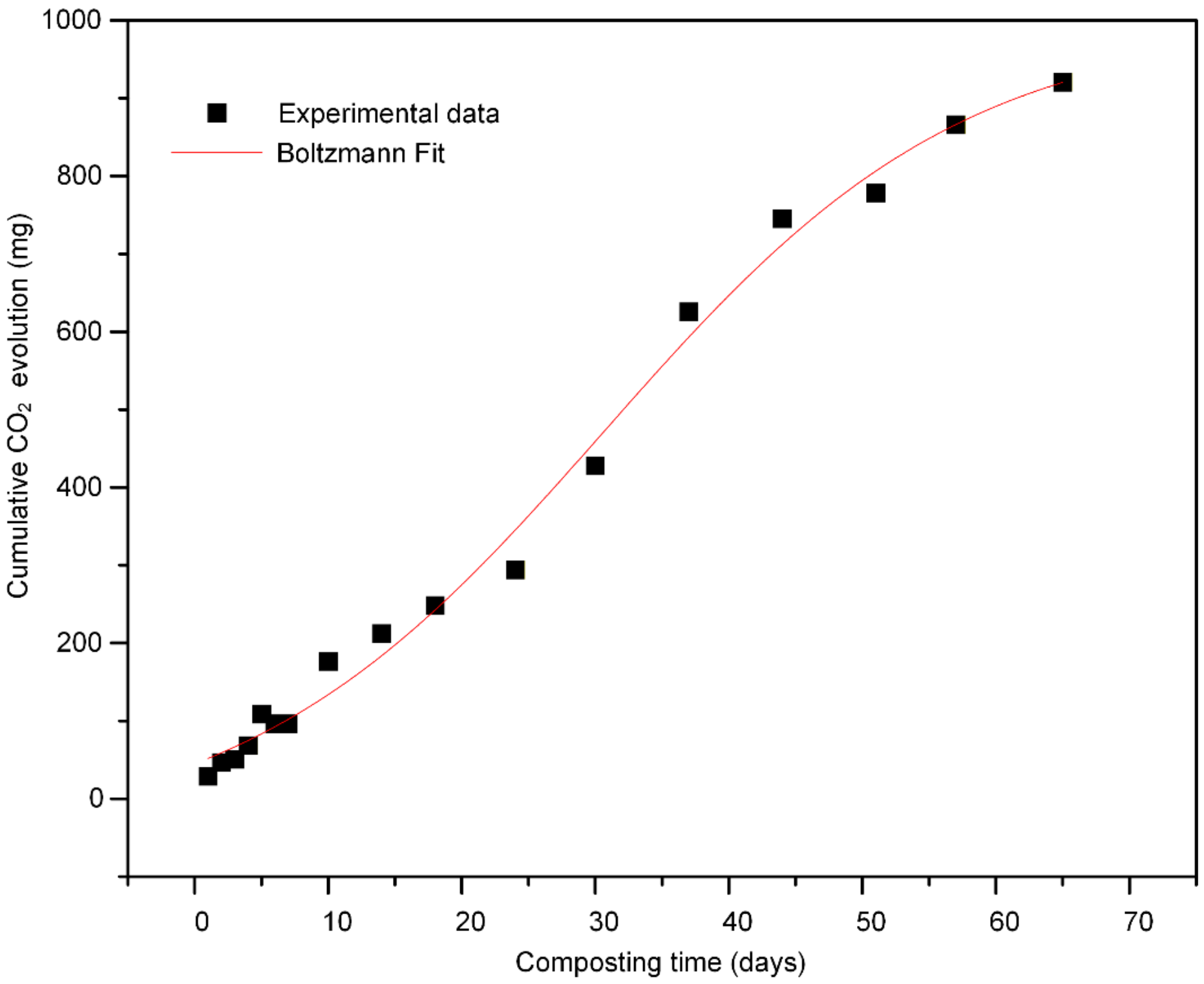

Fig. S14: Cumulative CO_2 evolution from ESLP during compost incubation. Cumulative net CO_2 evolution during 65 days of composting after subtraction of the compost blank. The symbols represent the experimental data, and the line represents an empirical Boltzmann sigmoidal function fit. Epoxy-functionalized syringolated lignin (ESL), ESL-pulp composite (ESLP).

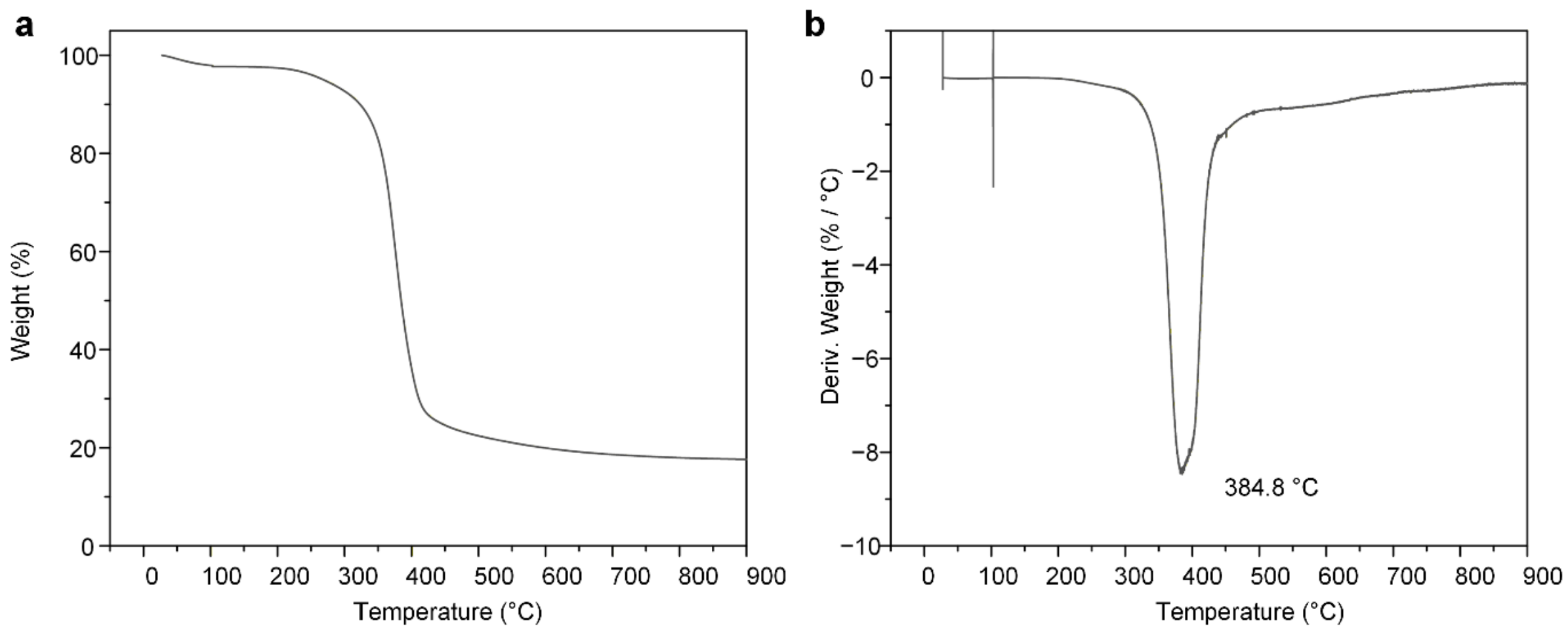

Fig. S15: a TG and b DTG curves of ESLP, represent the thermal stability of sample. The main DTG maximum occurs at 384.8 °C. Epoxy-functionalized syringolated lignin (ESL), ESL-pulp composite (ESLP).

Supplementary tables

Table S1. Compositional analysis of poplar and rapeseed straw.

	Cellulose (%)	Hemicellulose (%)	Lignin (%)	Ash (%)
Poplar	47.5	17.1	28.1	0.2
Rapeseed straw	34.4	18.2	18.5	2.0

Table S2. Determination of β–O–4 content and S/G ratio in lignins of poplar and rapeseed straw.

	β–O–4 (%)	S/G
Poplar	47.1	1.90
Rapeseed straw	56.8	1.77

Table S3. Optimization of syringol assisted reactive fractionations.

Sample	Poplar/syringol (wt/wt)	SL yield (%)	Pulp yield (%)	Pulp purity (%)
SL-0.2	1:0.2	34.0	49.4	95.6
SL-0.4	1:0.4	35.8	46.9	94.2
SL-0.6	1:0.6	37.7	46.7	96.5
Ash mimic	1:0.4	35.5	44.6	96.7

Table S4. GPC-derived molecular-weight characteristics of the sharp high-molecular-weight fraction and broad lower-molecular-weight fraction.

Sample	Retention time (min)	M_n (Da)	M_w (Da)	M_z (Da)	Đ (M_w/M_n)
SL-1	18.5 – 22.0	1431	1521	1628	1.06
SL-2	22.0 – 25.5	476	572	649	1.20
ESL-1	17.5 – 21.7	1967	2234	2579	1.14
ESL-2	21.7 – 24.5	678	725	772	1.07

Table S5. Apparent kinetic parameters for epoxy consumption during the ESL–cellulose reaction.

Curing temperature (°C)	Apparent rate constant (k_{app}) (min^{-1})	Uncentered (R^2)
140	0.00755	0.899
160	0.00865	0.974
180	0.00927	0.844

Arrhenius analysis of the obtained rate constants gave an apparent activation energy, $E_{a,app}$, of 8.03 kJ mol^{-1}, with an R^2 of 0.975.

Table S6. Comparison between the present reactive fractionation strategy and representative lignin-first/RCF strategies[7–12].

Aspect	Present work: syringol-assisted reactive fractionation	Representative lignin-first / RCF strategies
Main product target	Reactive syringolated lignin and cellulose pulp, recombined into covalently bonded lignin–cellulose composites	Lignin-derived aromatic monomers/oligomers, with carbohydrate-rich pulp as a coproduct
Product strategy	Material-oriented cellulose–lignin co-valorization without complete lignin depolymerization	Monomer-/chemical-oriented lignin depolymerization and stabilization
Feedstock adaptability	Metal-free system; suitable for ash-containing residual feedstocks such as rapeseed straw and ash-mimic poplar	Often sensitive to ash-rich feedstocks because inorganic salts may interfere with catalyst performance and recovery
Catalyst and reductive conditions	No transition-metal catalyst or external H_2 required	Commonly requires heterogeneous transition-metal catalysts and H_2 or hydrogen-donor systems
Process complexity	Fractionation directly installs a reactive handle on lignin; subsequent epoxy-functionalization enables direct composite fabrication	Requires catalyst recovery, lignin oil separation and often further downstream upgrading/polymerization before material use
Solvent and separation considerations	Excessive formic acid and syringol are recyclable.	Solvent loading, liquor recycling and solvent recovery are important cost and energy drivers in RCF scale-up
Product added value	Wet-stable bio-based composite with high wet-strength retention	Aromatic monomers/oligomers or polymer precursors; value depends on downstream separation, upgrading and application development

Aspect	Present work: syringol-assisted reactive fractionation	Representative lignin-first / RCF strategies
Industrialization considerations	Advantages: metal-free, ambient pressure, direct material formation. Challenges: epichlorohydrin use/recycling, solvent handling and hot-pressing energy	Advantages: established lignin-first chemistry and aromatic chemical production. Challenges: catalyst cost/recovery, H_2 supply, solvent recycling and monomer separation/upgrading
Overall positioning	Complementary material-oriented route for direct cellulose–lignin co-valorization, especially from residual streams	Powerful monomer-oriented lignin-first route for lignin valorization

Table S7. Comparison of ESLP with representative bio-based and conventional packaging materials.

Material	Tensile strength (MPa)	Tensile modulus (GPa)	Wet or humidity stability	Thermal behavior	Degradation or end-of-life behavior	Ref.
ESLP	46.7	2.5	Wet tensile strength: 33.9 MPa with 72.6% retention; wet modulus: 2.0 GPa with 80.0% retention. After 97 days at 25–85% RH, tensile strength remained at 25.9–28.3 MPa.	DTG T_{max} at 384.8 °C.	Progressive surface deterioration, deformation and partial fragmentation during 72 days of compost burial; blank-corrected cumulative CO_2 evolution reached 920.7 mg after 65 days.	This work
PLA, representative neat film	58.2	1.05	NR under a directly comparable water-immersion tensile test	Degradation onset: 211.9 °C; DTG T_{max}: 324.8 °C	A representative PLLA formulation reached 100.2 ± 3.7% biodegradation and complete disintegration under controlled industrial-composting conditions; compostable bags fully decomposed after seven weeks.	[13,14]
Lignin-containing cellulose nanopaper	255	6.4	Wet tensile strength: 83 MPa, corresponding to approximately 32.5% retention	DTG T_{max}: 339 °C	NR in the cited study	[15]
Kraft paper, representative literature values	55.8	NR	Wet tensile strength: 2.3 MPa, corresponding to approximately 4.1% retention	Major thermal mass loss occurred at approximately 280–350 °C, with a T_{max} near 350 °C.	Paper products are biodegradable under composting conditions, although the degradation rate depends strongly on pulping history, lignin content and additives.	[16–18]

Material	Tensile strength (MPa)	Tensile modulus (GPa)	Wet or humidity stability	Thermal behavior	Degradation or end-of-life behavior	Ref.
PET film	48.3–72.4	2.76–4.14	NR under a directly comparable water-immersion tensile test	DTG T_{max}: 441 °C	Not conventionally compostable; biological depolymerization generally requires specialized enzymes or microbial systems.	[19–21]

Appendixes

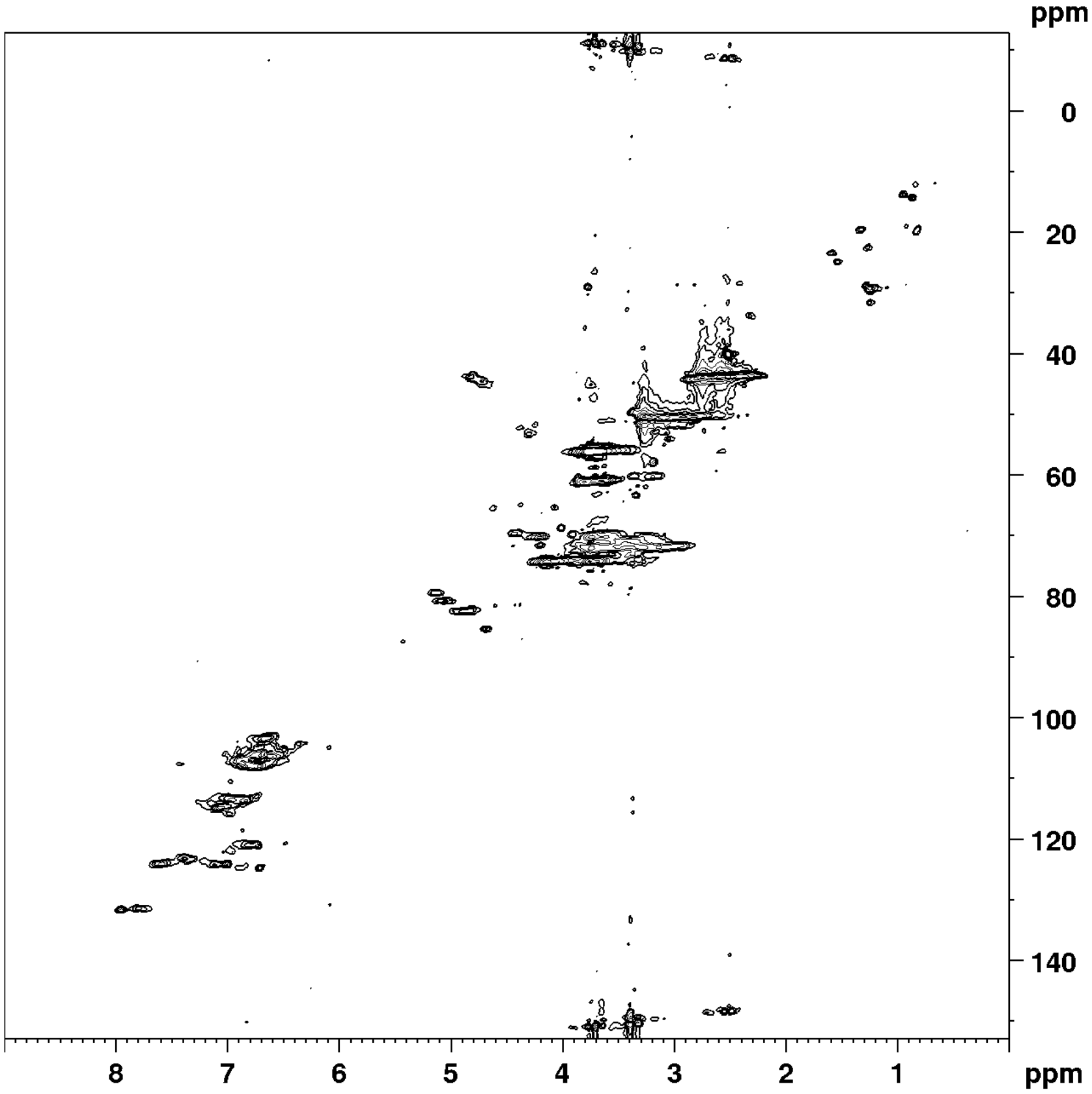

^1H–^{13}C HSQC NMR spectrum of ESL (poplar). HSQC NMR spectrum of ESL in DMSO-d_6, showing syringyl aromatic correlations and aliphatic correlations assigned to epoxy methylene/methine groups. Epoxy-functionalized syringolated lignin (ESL).

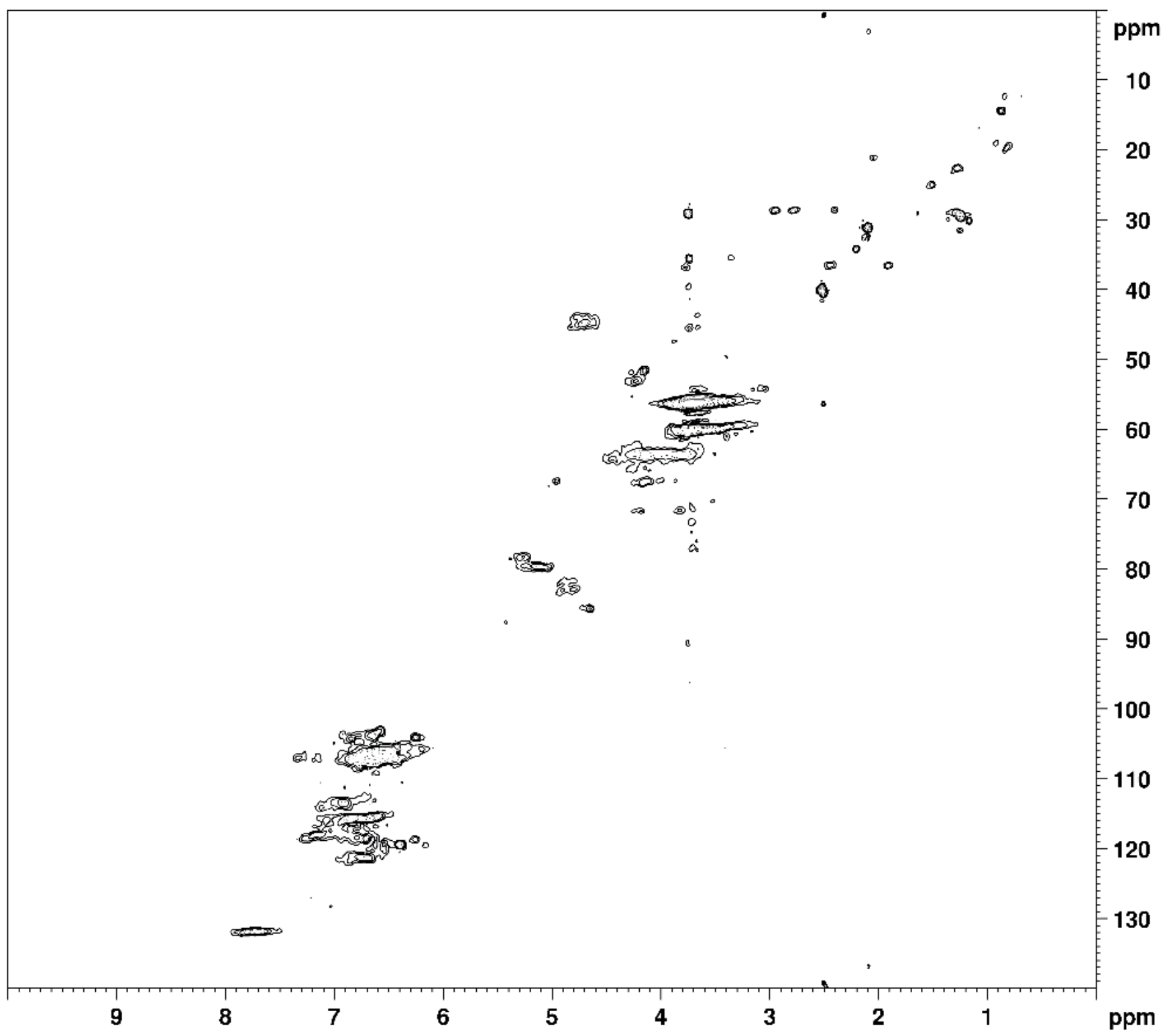

^{1}H–^{13}C HSQC NMR spectrum of SL from ash mimic experiment. HSQC NMR spectrum of SL in DMSO-d_6, showing syringyl aromatic correlations. Syringolated lignin (SL).

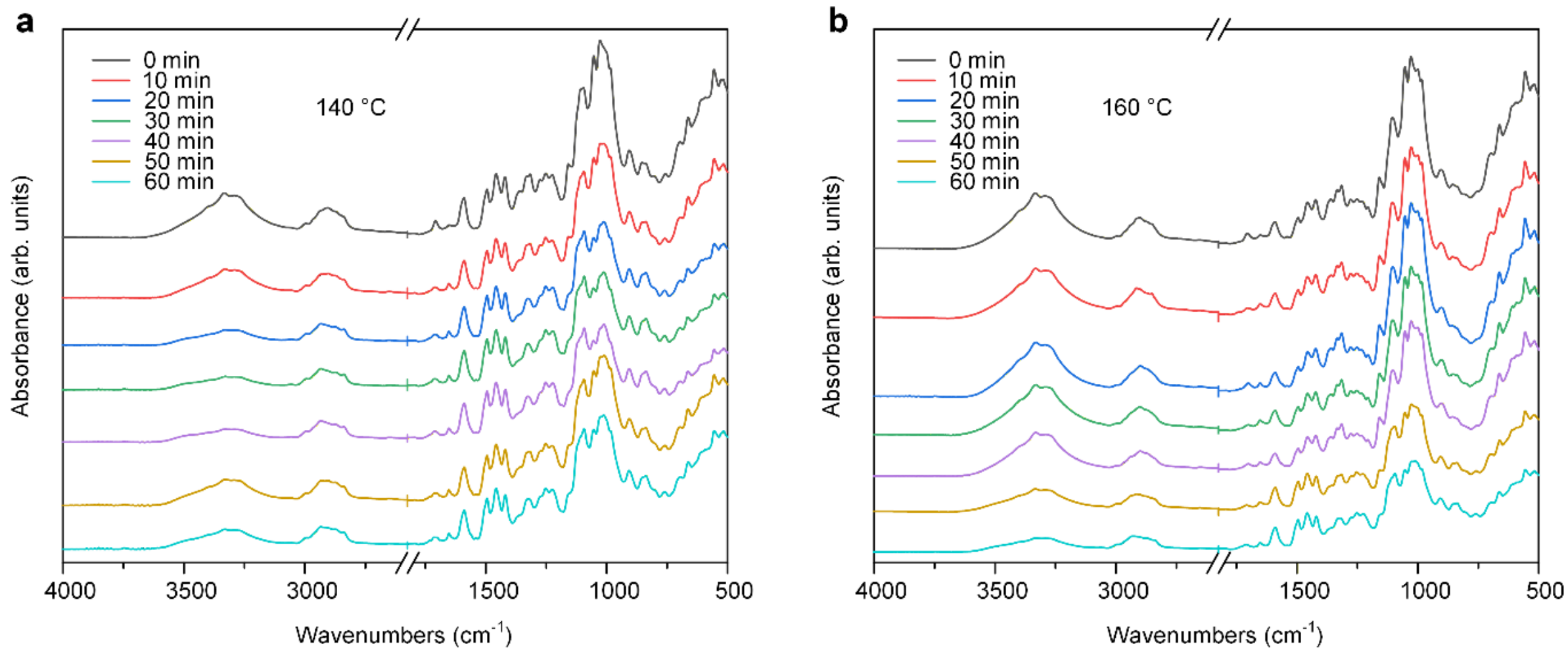

Kinetic experiments of ESLFP prepared under a 140 °C and b 160 °C. Epoxy-functionalized syringolated lignin (ESL), ESL-filter paper composite (ESLFP).

Supplementary References

1. Jaques, N. G. *et al.* Kinetic investigation of eggshell powders as biobased epoxy catalyzer. *Compos. Part B Eng.* **183**, 107651 (2020).
2. Tao, Z. *et al.* Mineralization and Transfer of Polymer-Derived Carbon from Biodegradable Mulch into the Soil Microbial Biomass and Organic Matter Pool. *Environ. Sci. Technol.* **59**, 25830–25841 (2025).
3. Polin, J. P., Carr, H. D., Whitmer, L. E., Smith, R. G. & Brown, R. C. Conventional and autothermal pyrolysis of corn stover: Overcoming the processing challenges of high-ash agricultural residues. *J. Anal. Appl. Pyrolysis* **143**, 104679 (2019).
4. Steenari, B.-M., Lundberg, A., Pettersson, H., Wilewska-Bien, M. & Andersson, D. Investigation of Ash Sintering during Combustion of Agricultural Residues and the Effect of Additives. *Energy Fuels* **23**, 5655–5662 (2009).
5. Liu, Y. *et al.* Ash chemistry in chemical looping process for biomass valorization: A review. *Chem. Eng. J.* **478**, 147429 (2023).
6. Li, J. *et al.* Investigation into the fusibility of biomass ashes and their mineral phase transformations at elevated temperatures by using the HT-XRD technique. *Biomass Bioenergy* **173**, 106812 (2023).
7. Abu-Omar, M. M. *et al.* Guidelines for performing lignin-first biorefining. *Energy Environ. Sci.* **14**, 262–292 (2021).
8. Bartling, A. W. *et al.* Techno-economic analysis and life cycle assessment of a biorefinery utilizing reductive catalytic fractionation. *Energy Environ. Sci.* **14**, 4147–4168 (2021).
9. Jang, J. H. *et al.* Multi-pass flow-through reductive catalytic fractionation. *Joule* **6**, 1859–1875 (2022).

10. Bugli, F. *et al.* Improved Reductive Catalytic Fractionation of Lignocellulosic Biomass through the Application of a Recyclable Magnetic Catalyst. *ACS Sustain. Chem. Eng.* **12**, 16638–16651 (2024).

11. Sun, Z. *et al.* Downstream Processing Strategies for Lignin-First Biorefinery. *ChemSusChem* **13**, 5199–5212 (2020).

12. Arts, W. *et al.* Stepping away from purified solvents in reductive catalytic fractionation: a step forward towards a disruptive wood biorefinery process. *Energy Environ. Sci.* **16**, 2518–2539 (2023).

13. Silverajah, V. S. G., Ibrahim, N. A., Yunus, W. M. Z. W., Hassan, H. A. & Woei, C. B. A Comparative Study on the Mechanical, Thermal and Morphological Characterization of Poly(lactic acid)/Epoxidized Palm Oil Blend. *Int. J. Mol. Sci.* **13**, 5878–5898 (2012).

14. Maragkaki, A. *et al.* Evaluation of Biodegradability of Polylactic Acid and Compostable Bags from Food Waste under Industrial Composting. *Sustainability* **15**, 15963 (2023).

15. Wang, Q. *et al.* Flexible cellulose nanopaper with high wet tensile strength, high toughness and tunable ultraviolet blocking ability fabricated from tobacco stalk via a sustainable method. *J. Mater. Chem. A* **6**, 13021–13030 (2018).

16. Zhang, J. *et al.* High-barrier, strong, and antibacterial paper fabricated by coating acetylated cellulose and cinnamaldehyde for food packaging. *Cellulose* **28**, 4371–4384 (2021).

17. Venelampi, O., Weber, A., Rönkkö, T. & Itävaara, M. The Biodegradation and Disintegration of Paper Products in the Composting Environment. *Compost Sci. Util.* **11**, 200–209 (2003).

18. Li, A. *et al.* Comparative analysis of the physical and mechanical properties of kraft paper and watercolor paper. *Maderas Cienc. Tecnol.* **23**, (2021).

19. Guan, Q.-F., Ling, Z.-C., Han, Z.-M., Yang, H.-B. & Yu, S.-H. Ultra-Strong, Ultra-Tough, Transparent, and Sustainable Nanocomposite Films for Plastic Substitute. *Matter* **3**, 1308–1317 (2020).

20. Turnbull, L., Liggat, J. J. & MacDonald, W. A. Thermal degradation chemistry of poly(ethylene naphthalate) – A study by thermal volatilisation analysis. *Polym. Degrad. Stab.* **98**, 2244–2258 (2013).

21. Soong, Y.-H. V., Sobkowicz, M. J. & Xie, D. Recent Advances in Biological Recycling of Polyethylene Terephthalate (PET) Plastic Wastes. *Bioengineering* **9**, 98 (2022).
