# Supplementary material for: Composites of covalently linked lignin and cellulose from one feedstock
Source: Nat Commun. 2026 Aug 27;17:9145. doi: 10.1038/s41467-026-77206-8 (PMC13522584; doi:10.1038/s41467-026-77206-8)
Supplement: Supplementary file 2 [file 41467_2026_77206_MOESM2_ESM.pdf]

nature portfolio

Peer Review File

Composites of covalently linked lignin and cellulose from one feedstock

Corresponding Author: Professor Joseph Samec

This file contains all reviewer reports in order by version, followed by all author rebuttals in order by version.

Attachments originally included by the reviewers as part of their assessment can be found at the end of this file.

Version 1:

Reviewer comments:

Reviewer #1

(Remarks to the Author)
This work proposes a metal-free, ambient-pressure reactive fractionation strategy that enables the simultaneous valorization of cellulose and lignin in lignocellulosic feedstocks, breaking through the limitation of prioritizing a single component (either cellulose or lignin) in traditional biomass conversion to truly achieve holistic biomass co-valorization; through in-situ syringolation modification of lignin and subsequent epoxy functionalization, epoxy-functionalized syringolated lignin (ESL) is constructed, and by forming covalently bonded composites with cellulose via epoxide ring-opening reaction, the material's wet mechanical stability is significantly improved, overcoming the inherent defect of deteriorated wet performance in traditional hydrogen-bonded cellulose materials; additionally, the technical route exhibits good tolerance to ash content in biomass feedstocks, allowing direct use of low-quality raw materials such as forestry residues (e.g., poplar tops and branches) and agricultural wastes (e.g., rapeseed straw) with high ash content, broadening the scope of feedstocks for biomass conversion and solving the key problem of catalyst poisoning by ash in traditional metal-catalyzed conversion routes.
Furthermore, the work provides a new technical paradigm for holistic biomass co-valorization, clarifies the reaction mechanism of lignin-cellulose covalent bonding, and enriches the preparation theory of biomass-based composites; the prepared composites exhibit mechanical properties comparable to traditional packaging materials (e.g., Kraft liner and PET) under both dry and wet conditions, offering a feasible solution for the bio-based substitution of fossil-derived packaging materials and aligning with global sustainable development strategies; additionally, the technical route features good scalability with mild reaction conditions (ambient pressure, no metal catalysts), lowering the threshold for industrial application and holding great significance for promoting the efficient utilization of biomass resources and the development of green material industries.
Overall, this study proposes an innovative holistic biomass co-valorization technology and successfully prepares lignin-cellulose covalent composites with excellent wet stability. The research topic has important scientific significance and application value. The experimental design is reasonable, the characterization methods are comprehensive, the data are sufficient and reliable, and the conclusions are clear and powerful. Although there are deficiencies in process parameter optimization, long-term stability evaluation, degradation performance research, and in-depth exploration of reaction mechanism, these can be effectively solved by appropriately supplementing experiments and improving analysis.
In summary, The core innovations of the study meet the publication requirements of Nature Communications, but targeted supplements and improvements are needed in terms of resource recycling, function expansion, and process practicality.
Specific decision: Major Revision.
Below are detailed comments and suggestions for improvement:.
1.Research Methods and Experimental Design
(1)The optimization of syringol dosage as a nucleophile in the reactive fractionation process is insufficiently reflected. It is recommended to supplement data on the effects of different syringol/biomass ratios on fractionation efficiency (cellulose purity, lignin extraction rate) and lignin modification effect, so as to clarify the basis for selecting optimal process parameters.
(2) The long-term stability evaluation of the composites is inadequate. The existing data only reflect short-term wet mechanical properties. It is suggested to supplement long-term aging experiments (e.g., 1-3 months) of the materials under different humidity environments to investigate the stability of the covalently bonded structure and the attenuation law of mechanical properties.
(3) A comprehensive performance comparison with commercial bio-based packaging materials is lacking. It is

recommended to add a systematic comparison with existing mainstream biomass-based packaging materials (e.g., polylactic acid, cellulose-based composites) in terms of mechanical properties, thermal stability, gas permeability, and degradation performance, to more intuitively highlight the application advantages of the materials in this study.
2. Research Methods and Experimental Design
(1) The optimization of syringol dosage as a nucleophile in the reactive fractionation process is insufficiently reflected. It is recommended to supplement data on the effects of different syringol/biomass ratios on fractionation efficiency (cellulose purity, lignin extraction rate) and lignin modification effect, so as to clarify the basis for selecting optimal process parameters.
(2) The long-term stability evaluation of the composites is inadequate. The existing data only reflect short-term wet mechanical properties. It is suggested to supplement long-term aging experiments (e.g., 1-3 months) of the materials under different humidity environments to investigate the stability of the covalently bonded structure and the attenuation law of mechanical properties.
(3) A comprehensive performance comparison with commercial bio-based packaging materials is lacking. It is recommended to add a systematic comparison with existing mainstream biomass-based packaging materials (e.g., polylactic acid, cellulose-based composites) in terms of mechanical properties, thermal stability, gas permeability, and degradation performance, to more intuitively highlight the application advantages of the materials in this study.
(4) The in-depth exploration of the reaction mechanism needs to be strengthened. Although XPS and FTIR confirm epoxide ring-opening and covalent bond formation, quantitative analysis of reaction kinetics (e.g., reaction rate constant, activation energy) is lacking. It is suggested to supplement reaction kinetic experiments at different temperatures to further improve the theoretical interpretation of the reaction mechanism.
3. Data Quality and Result Analysis
(1) The presentation of some characterization data is not detailed enough. For example, in GPC analysis, specific values of number-average molecular weight (Mn), weight-average molecular weight (Mw), and dispersity (Đ) are not provided, and only the molecular weight change is explained through curve trends. It is recommended to supplement specific data to enhance persuasiveness; the assignment of some characteristic peaks in FTIR spectra (e.g., the C-H vibration peak of the epoxy ring around 3056 cm-1) can be further supported by combining with literature data.
(2) The analysis of the influence mechanism of ash on the reaction process is not in-depth enough. Although the technology is mentioned to be tolerant to high ash content, the existing form of ash (e.g., alkali metal and alkaline earth metal compounds) in the reaction and its specific influence on reactive fractionation, epoxy functionalization, and other links (e.g., whether it participates in the reaction, whether it affects product purity) are not clarified. It is recommended to supplement relevant characterizations (e.g., ICP-OES analysis of ash element composition, XRD analysis of ash phase) and control experiments to clarify the internal reason for ash tolerance.
(3) The degradation performance of the composites is not involved. As a potential packaging material, biodegradability is an important evaluation index. It is recommended to supplement degradation experiments of the materials under natural environment or composting conditions to investigate their environmental friendliness and improve the performance evaluation system of the materials.
4.Conclusions and Discussion
(1) The comparison between this technology and "lignin-first" strategies (e.g., reductive catalytic fractionation, RCF) in the discussion is not comprehensive enough. It is recommended to conduct a systematic comparison from aspects of feedstock adaptability, process complexity, product added value, and industrialization cost to further highlight the competitive advantages of this technology.
(2) The discussion on the expansion of application scenarios for the composites is insufficient. The existing discussion mainly focuses on packaging materials. It is recommended to explore their potential applications in other fields (e.g., biomedical materials, agricultural mulch films, water treatment membranes) combined with the performance characteristics of the materials (e.g., wet stability, mechanical strength), so as to broaden the application perspective of the research.
(3) The prospect of industrialization of the technology in the conclusion section is too general. It is recommended to supplement the analysis of potential challenges in the industrialization process (e.g., raw material pretreatment, product separation and purification, cost control) and propose preliminary solutions to enhance the practical application guiding significance of the research.

Reviewer #2

(Remarks to the Author)
Dear Authors
It's interesting and new approach of making composite materials by combining lignin with cellulose via epichlorohydrin (ECH), which deserves its publication. However there is a few points to clarified prior to its publication with minor revision:
1. It is necessary to provide details of preventing recondensation (or self-condensation) of lignin in the isolation process in relation with using formic acid.
2. The authors suggest that the composites could be used packaging materials, which need some additional data for this application.
3. It's not clear whether the cellulose crystallinity has been changed before and after the reaction with lignin in the composites.
4. The hot-pressing at 180C and 3.5 MPa for 1 hour is a harsh condition compared to conventional lignocellulosic materials. The authors need to justify if his is an optimum condition.

Reviewer #3

(Remarks to the Author)
I co-reviewed this manuscript with one of the reviewers who provided the listed reports. This is part of the Nature

Communications initiative to facilitate training in peer review and to provide appropriate recognition for Early Career Researchers who co-review manuscripts.

Reviewer #4

(Remarks to the Author)
This manuscript presents a compelling and timely strategy to co-valorize lignin and cellulose from a single lignocellulosic feedstock via metal-free reactive fractionation, followed by epoxy-functionalization and covalent coupling to produce composites with strong wet-strength retention. The overall approach is innovative and the results are promising. I recommend publication after the authors address the following minor points to improve clarity and strengthen the mechanistic and structural interpretation:
1, The authors provide HSQC spectra of SL and ESL; however, for a more complete structural comparison, the HSQC features of native poplar lignin should be discussed.
2, The composite is prepared using an ESL/pulp mass ratio of 1:1. Did the authors explore other ratios?
3, The authors reported a meta/para ratio for SL obtained from reactive fractionation. Please provide a brief explanation of how this ratio is determined and what it implies.
4, Hot-pressing and densification can themselves improve the mechanical strength of cellulose networks. How did the authors distinguish the contribution from densified morphology versus covalent ESL–cellulose linkages.
5, The strategy compellingly co-utilizes cellulose and lignin from one feedstock. Given the manuscript's "whole-biomass" framing, what's the fate of the hemicellulose fraction in this process?

Version 2:

Reviewer comments:

Reviewer #1

(Remarks to the Author)
The paper retains its three central breakthroughs: metal-free co-fractionation run under ambient pressure, covalently crosslinked lignin-cellulose materials with robust mechanical properties in humid environments, and the ability to process high-ash residual biomass. Every extra experiment the team ran directly responded to the reviewers' concerns, without weakening the study's central advances. This also makes its unique selling points clearer: the covalent crosslink design that fixes poor wet strength, broad suitability for farm and forest waste, and an affordable production route free of metal catalysts. Every new claim the authors put forward comes with hard numerical data, supporting charts and supplementary tables, alongside thorough control experiments. A full set of characterisation tools—NMR, XPS, FTIR, XRD, GPC and reaction kinetic measurements—together deliver solid proof for the underlying chemical mechanisms. Where properties like gas permeability were not measured, the authors openly state this limitation and avoid overhyping the material's real-world performance.
Earlier gaps flagged around circular resource use, industrial feasibility and narrow functional scope have all been fully addressed. The revised manuscript now features detailed analysis on recovering value from hemicellulose waste streams, the material's resistance to ash contaminants, its biodegradable nature, plus a balanced breakdown of the upsides and barriers to large-scale manufacturing. This fixes the earlier shortfall in detailed sustainability reasoning.

Reviewer #2

(Remarks to the Author)
Dear Authors
The revision has been improved with additional data and more clarifications in the responses to the reviewers' comments, which deserves its publication in the current form.
No more comments.
sincerely

Reviewer #3

(Remarks to the Author)
I co-reviewed this manuscript with one of the reviewers who provided the listed reports. This is part of the Nature Communications initiative to facilitate training in peer review and to provide appropriate recognition for Early Career Researchers who co-review manuscripts.

Reviewer #4

(Remarks to the Author)
The paper has been carefully revised. It should be accepted.

Open Access This Peer Review File is licensed under a Creative Commons Attribution 4.0 International License, which permits use, sharing, adaptation, distribution and reproduction in any medium or format, as long as you give appropriate credit to the original author(s) and the source, provide a link to the Creative Commons license, and indicate if changes were made.
In cases where reviewers are anonymous, credit should be given to 'Anonymous Referee' and the source.
The images or other third party material in this Peer Review File are included in the article's Creative Commons license, unless indicated otherwise in a credit line to the material. If material is not included in the article's Creative Commons license and your intended use is not permitted by statutory regulation or exceeds the permitted use, you will need to obtain permission directly from the copyright holder.
To view a copy of this license, visit https://creativecommons.org/licenses/by/4.0/

Response to Reviewer's comments

The entire manuscript has been revised according to the editor and reviewers' comments. The comments of the editor and reviewers are presented below in *italics with gray shading*, and our responses to those comments are shown in blue font with underline. The modifications and improvements made to the manuscript are also shown in blue font.

Reviewer #1:

This work proposes a metal-free, ambient-pressure reactive fractionation strategy that enables the simultaneous valorization of cellulose and lignin in lignocellulosic feedstocks, breaking through the limitation of prioritizing a single component (either cellulose or lignin) in traditional biomass conversion to truly achieve holistic biomass co-valorization; through in-situ syringolation modification of lignin and subsequent epoxy functionalization, epoxy-functionalized syringolated lignin (ESL) is constructed, and by forming covalently bonded composites with cellulose via epoxide ring-opening reaction, the material's wet mechanical stability is significantly improved, overcoming the inherent defect of deteriorated wet performance in traditional hydrogen-bonded cellulose materials; additionally, the technical route exhibits good tolerance to ash content in biomass feedstocks, allowing direct use of low-quality raw materials such as forestry residues (e.g., poplar tops and branches) and agricultural wastes (e.g., rapeseed straw) with high ash content, broadening the scope of feedstocks for biomass conversion and solving the key problem of catalyst

poisoning by ash in traditional metal-catalyzed conversion routes.

Furthermore, the work provides a new technical paradigm for holistic biomass co-valorization, clarifies the reaction mechanism of lignin-cellulose covalent bonding, and enriches the preparation theory of biomass-based composites; the prepared composites exhibit mechanical properties comparable to traditional packaging materials (e.g., Kraft liner and PET) under both dry and wet conditions, offering a feasible solution for the bio-based substitution of fossil-derived packaging materials and aligning with global sustainable development strategies; additionally, the technical route features good scalability with mild reaction conditions (ambient pressure, no metal catalysts), lowering the threshold for industrial application and holding great significance for promoting the efficient utilization of biomass resources and the development of green material industries.

Overall, this study proposes an innovative holistic biomass co-valorization technology and successfully prepares lignin-cellulose covalent composites with excellent wet stability. The research topic has important scientific significance and application value. The experimental design is reasonable, the characterization methods are comprehensive, the data are sufficient and reliable, and the conclusions are clear and powerful. Although there are deficiencies in process parameter optimization, long-term stability evaluation, degradation performance research, and in-depth exploration of reaction mechanism, these can be effectively solved by appropriately supplementing experiments and improving analysis.

In summary, the core innovations of the study meet the publication requirements of Nature

Communications, but targeted supplements and improvements are needed in terms of resource recycling, function expansion, and process practicality.

Specific decision: ***Major Revision****.*

Below are detailed comments and suggestions for improvement:.

Response: We sincerely thank the reviewer for the careful evaluation of our manuscript and for the constructive comments, which have helped us improve the quality and clarity of the work. We have revised the manuscript accordingly, and our point-by-point responses are provided below.

1. Research Methods and Experimental Design

(1) The optimization of syringol dosage as a nucleophile in the reactive fractionation process is insufficiently reflected. It is recommended to supplement data on the effects of different syringol/biomass ratios on fractionation efficiency (cellulose purity, lignin extraction rate) and lignin modification effect, so as to clarify the basis for selecting optimal process parameters.

Response: We thank the reviewer for this valuable suggestion. We agree that the optimization of syringol dosage is important for clarifying the process design and for justifying the selected reaction conditions. To address this point, we have supplemented additional experiments using different biomass/syringol mass ratios during reactive fractionation and evaluated their effects on SL yield, pulp yield and pulp purity. The new data have been added to the Supporting Information

as Table S3. Specifically, biomass/syringol mass ratios of 1:0.2, 1:0.4 and 1:0.6 were investigated under otherwise identical reaction conditions. Increasing the syringol loading from 1:0.2 to 1:0.4 increased the SL yield from 34.0% to 35.8%, while maintaining a high pulp purity of 94.2%. Further increasing the syringol loading to 1:0.6 gave a slightly higher SL yield of 37.7% and a pulp purity of 96.5%. However, compared with the 1:0.4 condition, this represents only a 1.9 percentage point increase in SL yield while requiring 50% more syringol. Therefore, the additional benefit of using excess syringol was not proportional to the increased reagent input. Because the central objective of this work is the co-valorization of both cellulose and lignin fractions, the optimization was not based solely on maximizing pulp purity or lignin yield independently. Instead, we selected the reaction condition by considering the balance among SL yield, pulp yield, pulp purity, reagent consumption and downstream purification practicality. The 1:0.4 biomass/syringol ratio afforded a high SL yield of 35.8%, which is already close to the calculated theoretical maximum yield of 38.0%, together with a cellulose-rich pulp of 94.2% purity. This condition therefore provides an efficient and practical compromise for producing both a reactive lignin stream and a high-purity cellulose pulp. We have revised the manuscript to clarify this optimization rationale and have added the corresponding data to Table S3 in the Supporting Information, as shown below:

Page 4

Reactive fractionation was performed on poplar wood (*Populus trichocarpa*) using syringol as the nucleophile and formic acid as the solvent at reflux (**Error! Reference source not found.**a)[42]. The process furnished two isolable streams: a light-colored soluble fraction, *vide infra*, and a cellulose-rich pulp readily separated by filtration that accounted for 46.9% of the initial biomass. The pulp

contained 94.2% cellulose and 1.9% hemicellulose, with 3.3% lignin. By comparison, native poplar comprised 47.5% cellulose, 17.1% hemicellulose and 28.1% lignin (Table S1). The low residual hemicellulose content in the pulp indicates that hemicellulose was largely hydrolyzed into xylose and xylose-derived soluble species and transferred to the hydrophilic filtrate during the formic-acid-based fractionation[42]. In addition, xylose has been demonstrated as a suitable feedstock for formic acid production, providing a potential route for future process integration and solvent regeneration[43]. To evaluate the influence of syringol loading, biomass/syringol mass ratios of 1:0.2, 1:0.4 and 1:0.6 were further compared under otherwise identical conditions (Table S3). Increasing the syringol loading from 1:0.2 to 1:0.4 increased the SL yield from 34.0% to 35.8%, while maintaining a high pulp purity of 94.2%. A further increase to 1:0.6 led to a slightly higher SL yield of 37.7% and a pulp purity of 96.5%; however, this modest improvement required 50% more syringol. Therefore, the biomass/syringol ratio of 1:0.4 was selected as a balanced condition considering SL yield, pulp quality, reagent economy and downstream purification practicality. Together, these results indicate that the reactive fractionation achieves efficient delignification while delivering a functionalized lignin stream alongside exceptionally pure cellulose.

SI

Table S1. Optimization of syringol assisted reactive fractionations.

Sample	Poplar/syringol (wt/wt)	SL yield (%)	Pulp yield (%)	Pulp purity (%)
SL-0.2	1:0.2	34.0	49.4	95.6
SL-0.4	1:0.4	35.8	46.9	94.2
SL-0.6	1:0.6	37.7	46.7	96.5
Ash mimic	1:0.4	35.5	44.6	96.7

(2) The long-term stability evaluation of the composites is inadequate. The existing data only reflect short-term wet mechanical properties. It is suggested to supplement long-term aging experiments (e.g., 1-3 months) of the materials under different humidity environments to investigate the stability of the covalently bonded structure and the attenuation law of mechanical properties.

Response: We sincerely thank the reviewer for this valuable suggestion. We agree that long-term

stability under different humidity conditions is important for evaluating the practical applicability of ESLP, particularly for packaging applications involving prolonged exposure to variable moisture environments. To address this concern, ESLP specimens were aged at room temperature under controlled relative humidities of 25%, 55%, 75% and 85% for 97 days using saturated salt solutions. Their tensile properties were subsequently evaluated and are now presented in Fig. 7a–b of the revised manuscript.

After 97 days of aging, the tensile strength remained within a relatively narrow range of 25.9–28.3 MPa across all investigated humidity conditions, corresponding to approximately 55–61% of the original tensile strength. No clear humidity-dependent decrease in tensile strength was observed between 25% and 85% RH, indicating that ESLP retained substantial load-bearing capacity after prolonged humidity exposure.

The tensile modulus was more sensitive to high humidity. It remained approximately 2.0 GPa after aging at 25% and 55% RH, but decreased to 1.28 and 1.01 GPa at 75% and 85% RH, respectively. This reduction in stiffness is attributed to moisture-induced plasticization and swelling of the cellulose-rich phase, which increase molecular mobility and inter-fiber deformation. Nevertheless, the retention of substantial tensile strength under all investigated conditions demonstrates that the covalently coupled ESL–cellulose network maintained long-term mechanical integrity.

The new 97-day humidity-aging results have been added to Fig. 7a–b, and the corresponding

discussion has been included in the revised manuscript, shown as below:

Page 15

While the contact-angle and wet-mechanical measurements demonstrate the immediate resistance of ESLP to water exposure, its practical use also requires retention of mechanical integrity during prolonged exposure to humid environments. The long-term humidity stability of ESLP was further evaluated after aging for 97 days at 25–85% RH (Fig. 2a–b). The aged samples retained tensile strengths of 25.9–28.3 MPa, corresponding to approximately 55–61% of the original value, with no clear humidity-dependent decrease across the investigated range. In contrast, the tensile modulus remained approximately 2.0 GPa after aging at 25% and 55% RH but decreased to 1.28 and 1.01 GPa at 75% and 85% RH, respectively. The lower modulus observed after aging at 75% and 85% RH suggests that prolonged high-humidity exposure induced residual or only partially reversible changes in the cellulose-rich network. Nevertheless, the substantial tensile strength retained after 97 days indicates that the covalently coupled ESL–cellulose network maintains long-term load-bearing integrity under prolonged humidity exposure.

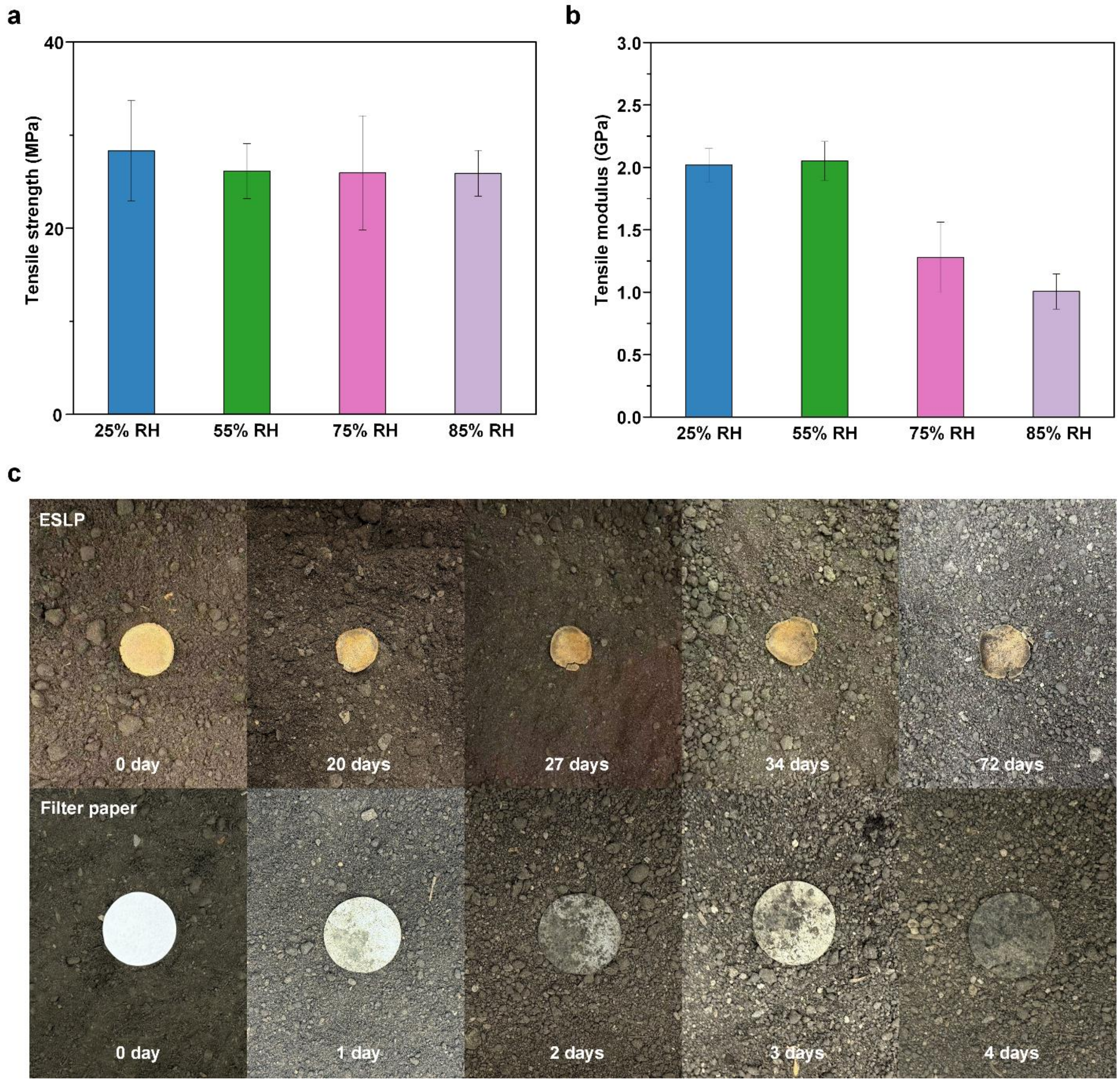

Fig. 1: Long-term humidity stability and composting degradation behavior of ESLP. a Tensile strength and **b** tensile modulus of ESLP after aging for 97 days at different relative humidities at room temperature. Error bars represent standard deviations. **c** Representative photographs showing the macroscopic disintegration of ESLP and filter paper during composting.

(3) A comprehensive performance comparison with commercial bio-based packaging materials is lacking. It is recommended to add a systematic comparison with existing mainstream biomass-based packaging materials (e.g., polylactic acid, cellulose-based composites) in terms of mechanical properties, thermal stability, gas permeability, and degradation performance, to more intuitively highlight the application advantages of the materials in this study.

Response: We thank the reviewer for this constructive suggestion. We agree that a systematic comparison with representative bio-based and conventional packaging materials is important for clarifying the practical positioning and application advantages of ESLP.

To address this point, we have added a comprehensive comparison with representative PLA, lignin-containing cellulose nanopaper, kraft paper and PET film in Table S7 of the Supporting Information. The comparison includes tensile strength, tensile modulus, wet or humidity stability, thermal behavior and degradation or end-of-life characteristics. Because the reported values were obtained using different formulations, conditioning procedures, specimen dimensions and testing standards, they are presented as representative literature benchmarks rather than as a strict performance ranking.

ESLP exhibited a tensile strength of 46.7 MPa and a tensile modulus of 2.5 GPa, placing its dry mechanical properties within the representative ranges reported for PLA, kraft paper and PET. Although the lignin-containing cellulose nanopaper showed substantially higher dry tensile strength, the principal advantage of ESLP lies in its moisture-resistant mechanical performance.

ESLP retained a wet tensile strength of 33.9 MPa and a wet tensile modulus of 2.0 GPa, corresponding to 72.6% and 80.0% retention, respectively. In comparison, the representative lignin-containing cellulose nanopaper and kraft paper retained approximately 32.5% and 4.1% of their dry tensile strengths under their reported wet-test conditions. Furthermore, after 97 days of aging at 25–85% RH, ESLP maintained tensile strengths of 25.9–28.3 MPa, demonstrating substantial long-term load-bearing capacity under prolonged humidity exposure.

The final ESLP composite showed a DTG maximum at 384.8 °C, which was higher than the representative values listed for PLA, lignin-containing cellulose nanopaper and kraft paper, although lower than that of PET. This result indicates that ESLP possesses sufficient thermal stability for many non-high-temperature packaging applications.

The degradation behavior was evaluated through complementary compost-burial and compost-respiration experiments. ESLP exhibited progressive surface deterioration, deformation and partial fragmentation during 72 days of compost burial. In addition, the blank-corrected cumulative CO_2 evolution reached 920.7 mg after 65 days, providing evidence of progressive microbial degradation under controlled composting conditions. Thus, ESLP combines service-relevant humidity stability with susceptibility to biological degradation under an appropriate end-of-life environment.

We respectfully acknowledge that gas permeability was not measured in the present study. The ESLP material developed here was primarily designed as a proof-of-concept platform to demonstrate covalent cellulose–lignin co-valorization and to improve the mechanical stability of

cellulose-based materials under wet and humid conditions. Its current fibrous architecture was not specifically engineered or sufficiently densified to form a continuous gas-barrier layer. Consequently, gas-permeability measurements at this stage would be strongly governed by the porosity, fiber packing, thickness and incomplete densification of the sheet, rather than solely reflecting the intrinsic barrier potential of the covalently bonded ESL–cellulose network. Direct comparison with dense commercial films such as PLA and PET could therefore be misleading because of the substantial differences in material architecture and intended function.

We sincerely apologize that reliable gas-permeability data could not be provided within the scope of the present revision, and we hope that the reviewer will understand this limitation. To avoid unsupported conclusions, gas permeability was not included in the quantitative comparison. We have instead explicitly acknowledged in the revised manuscript that gas-barrier performance remains an important parameter for future application-specific evaluation. Further development will require optimization of fiber packing, porosity, sheet thickness, surface continuity and potentially additional densification or coating strategies before oxygen, carbon dioxide and water-vapor permeability can be meaningfully assessed.

Overall, the comparison shows that the competitive advantage of ESLP lies in the combined balance of packaging-relevant mechanical strength, high wet-strength retention, long-term humidity stability, thermal stability and progressive degradation under composting conditions. The new comparison has been added as Table S7 in the Supporting Information, and the corresponding

discussion has been included in the revised manuscript, shown as below:

Page 17–18

To contextualize the packaging potential of ESLP, its performance was compared with representative PLA, lignin-containing cellulose nanopaper, kraft paper and PET film (Table S7). ESLP exhibited a tensile strength and modulus of 46.7 MPa and 2.5 GPa, respectively, which are within the representative ranges of widely used packaging materials. Its distinguishing feature is its moisture-resistant mechanical performance: ESLP retained 33.9 MPa wet tensile strength and a wet modulus of 2.0 GPa, corresponding to 72.6% and 80.0% retention, respectively. These strength-retention values are higher than those reported for the representative lignin-containing cellulose nanopaper and kraft paper included in the comparison. ESLP also showed a DTG maximum at 384.8 °C (Fig. S15) and retained tensile strengths of 25.9–28.3 MPa after 97 days of humidity aging. Together with its progressive macroscopic deterioration and blank-corrected CO_2 evolution under composting conditions, these results demonstrate a balance between service-stage moisture stability and end-of-life biological degradability.

SI

Table S2. Comparison of ESLP with representative bio-based and conventional packaging materials.

Material	Tensile strength (MPa)	Tensile modulus (GPa)	Wet or humidity stability	Thermal behavior	Degradation or end-of-life behavior	Ref.
ESLP	46.7	2.5	Wet tensile strength: 33.9 MPa with 72.6% retention; wet modulus: 2.0 GPa with 80.0% retention. After 97 days at 25–85% RH, tensile strength remained at 25.9–28.3 MPa.	DTG T_{max} at 384.8°C.	Progressive surface deterioration, deformation and partial fragmentation during 72 days of compost burial; blank-corrected cumulative CO_2 evolution reached 920.7 mg after 65 days.	This work
PLA, representative	58.2	1.05	NR under a directly comparable water-	Degradation onset: 211.9 °C; DTG T_{max}:	A representative PLLA formulation reached 100.2 ± 3.7% biodegradation	15,16

Material	Tensile strength (MPa)	Tensile modulus (GPa)	Wet or humidity stability	Thermal behavior	Degradation or end-of-life behavior	Ref.
neat film			immersion tensile test	324.8 °C	and complete disintegration under controlled industrial-composting conditions; compostable bags fully decomposed after seven weeks.	
Lignin-containing cellulose nanopaper	255	6.4	Wet tensile strength: 83 MPa, corresponding to approximately 32.5% retention	DTG T_{max}: 339 °C	NR in the cited study	[17]
Kraft paper, representative literature values	55.8	NR	Wet tensile strength: 2.3 MPa, corresponding to approximately 4.1% retention	Major thermal mass loss occurred at approximately 280–350 °C, with a T_{max} near 350 °C.	Paper products are biodegradable under composting conditions, although the degradation rate depends strongly on pulping history, lignin content and additives.	[18–20]
PET film	48.3–72.4	2.76–4.14	NR under a directly comparable water-immersion tensile test	DTG maximum: 441 °C	Not conventionally compostable; biological depolymerization generally requires specialized enzymes or microbial systems.	[21–23]

2. Research Methods and Experimental Design

(1) The optimization of syringol dosage as a nucleophile in the reactive fractionation process is insufficiently reflected. It is recommended to supplement data on the effects of different syringol/biomass ratios on fractionation efficiency (cellulose purity, lignin extraction rate) and lignin modification effect, so as to clarify the basis for selecting optimal process parameters.

Response: We thank the reviewer for this valuable suggestion. We agree that the optimization of

syringol dosage is important for clarifying the process design and for justifying the selected reaction conditions. To address this point, we have supplemented additional experiments using different biomass/syringol mass ratios during reactive fractionation and evaluated their effects on SL yield, pulp yield and pulp purity. The new data have been added to the Supporting Information as Table S3. Specifically, biomass/syringol mass ratios of 1:0.2, 1:0.4 and 1:0.6 were investigated under otherwise identical reaction conditions. Increasing the syringol loading from 1:0.2 to 1:0.4 increased the SL yield from 34.0% to 35.8%, while maintaining a high pulp purity of 94.2%. Further increasing the syringol loading to 1:0.6 gave a slightly higher SL yield of 37.7% and a pulp purity of 96.5%. However, compared with the 1:0.4 condition, this represents only a 1.9 percentage point increase in SL yield while requiring 50% more syringol. Therefore, the additional benefit of using excess syringol was not proportional to the increased reagent input. Because the central objective of this work is the co-valorization of both cellulose and lignin fractions, the optimization was not based solely on maximizing pulp purity or lignin yield independently. Instead, we selected the reaction condition by considering the balance among SL yield, pulp yield, pulp purity, reagent consumption and downstream purification practicality. The 1:0.4 biomass/syringol ratio afforded a high SL yield of 35.8%, which is already close to the calculated theoretical maximum yield of 38.0%, together with a cellulose-rich pulp of 94.2% purity. This condition therefore provides an efficient and practical compromise for producing both a reactive lignin stream and a high-purity cellulose pulp. We have revised the manuscript to clarify this optimization rationale and have added the corresponding data to Table S3 in the Supporting Information, as shown below:

Page 4

Reactive fractionation was performed on poplar wood (*Populus trichocarpa*) using syringol as the nucleophile and formic acid as the solvent at reflux (**Error! Reference source not found.**a)[42]. The process furnished two isolable streams: a light-colored soluble fraction, *vide infra*, and a cellulose-rich pulp readily separated by filtration that accounted for 46.9% of the initial biomass. The pulp contained 94.2% cellulose and 1.9% hemicellulose, with 3.3% lignin. By comparison, native poplar comprised 47.5% cellulose, 17.1% hemicellulose and 28.1% lignin (Table S1). The low residual hemicellulose content in the pulp indicates that hemicellulose was largely hydrolyzed into xylose and xylose-derived soluble species and transferred to the hydrophilic filtrate during the formic-acid-based fractionation[42]. In addition, xylose has been demonstrated as a suitable feedstock for formic acid production, providing a potential route for future process integration and solvent regeneration[43]. To evaluate the influence of syringol loading, biomass/syringol mass ratios of 1:0.2, 1:0.4 and 1:0.6 were further compared under otherwise identical conditions (Table S3). Increasing the syringol loading from 1:0.2 to 1:0.4 increased the SL yield from 34.0% to 35.8%, while maintaining a high pulp purity of 94.2%. A further increase to 1:0.6 led to a slightly higher SL yield of 37.7% and a pulp purity of 96.5%; however, this modest improvement required 50% more syringol. Therefore, the biomass/syringol ratio of 1:0.4 was selected as a balanced condition considering SL yield, pulp quality, reagent economy and downstream purification practicality. Together, these results indicate that the reactive fractionation achieves efficient delignification while delivering a functionalized lignin stream alongside exceptionally pure cellulose.

SI

Table S3. Optimization of syringol assisted reactive fractionations.

Sample	Poplar/syringol (wt/wt)	SL yield (%)	Pulp yield (%)	Pulp purity (%)
SL-0.2	1:0.2	34.0	49.4	95.6
SL-0.4	1:0.4	35.8	46.9	94.2
SL-0.6	1:0.6	37.7	46.7	96.5
Ash mimic	1:0.4	35.5	44.6	96.7

(2) The long-term stability evaluation of the composites is inadequate. The existing data only reflect short-term wet mechanical properties. It is suggested to supplement long-term aging experiments

(e.g., 1-3 months) of the materials under different humidity environments to investigate the stability of the covalently bonded structure and the attenuation law of mechanical properties.

Response: We sincerely thank the reviewer for this valuable suggestion. We agree that long-term stability under different humidity conditions is important for evaluating the practical applicability of ESLP, particularly for packaging applications involving prolonged exposure to variable moisture environments. To address this concern, ESLP specimens were aged at room temperature under controlled relative humidities of 25%, 55%, 75% and 85% for 97 days using saturated salt solutions. Their tensile properties were subsequently evaluated and are now presented in Fig. 7a–b of the revised manuscript.

After 97 days of aging, the tensile strength remained within a relatively narrow range of 25.9–28.3 MPa across all investigated humidity conditions, corresponding to approximately 55–61% of the original tensile strength. No clear humidity-dependent decrease in tensile strength was observed between 25% and 85% RH, indicating that ESLP retained substantial load-bearing capacity after prolonged humidity exposure.

The tensile modulus was more sensitive to high humidity. It remained approximately 2.0 GPa after aging at 25% and 55% RH, but decreased to 1.28 and 1.01 GPa at 75% and 85% RH, respectively. This reduction in stiffness is attributed to moisture-induced plasticization and swelling of the cellulose-rich phase, which increase molecular mobility and inter-fiber deformation. Nevertheless, the retention of substantial tensile strength under all investigated conditions

demonstrates that the covalently coupled ESL–cellulose network maintained long-term mechanical integrity.

The new 97-day humidity-aging results have been added to Fig. 7a–b, and the corresponding discussion has been included in the revised manuscript, shown as below:

Page 15

While the contact-angle and wet-mechanical measurements demonstrate the immediate resistance of ESLP to water exposure, its practical use also requires retention of mechanical integrity during prolonged exposure to humid environments. The long-term humidity stability of ESLP was further evaluated after aging for 97 days at 25–85% RH (Fig. 2a–b). The aged samples retained tensile strengths of 25.9–28.3 MPa, corresponding to approximately 55–61% of the original value, with no clear humidity-dependent decrease across the investigated range. In contrast, the tensile modulus remained approximately 2.0 GPa after aging at 25% and 55% RH but decreased to 1.28 and 1.01 GPa at 75% and 85% RH, respectively. The lower modulus observed after aging at 75% and 85% RH suggests that prolonged high-humidity exposure induced residual or only partially reversible changes in the cellulose-rich network. Nevertheless, the substantial tensile strength retained after 97 days indicates that the covalently coupled ESL–cellulose network maintains long-term load-bearing integrity under prolonged humidity exposure.

Page 16

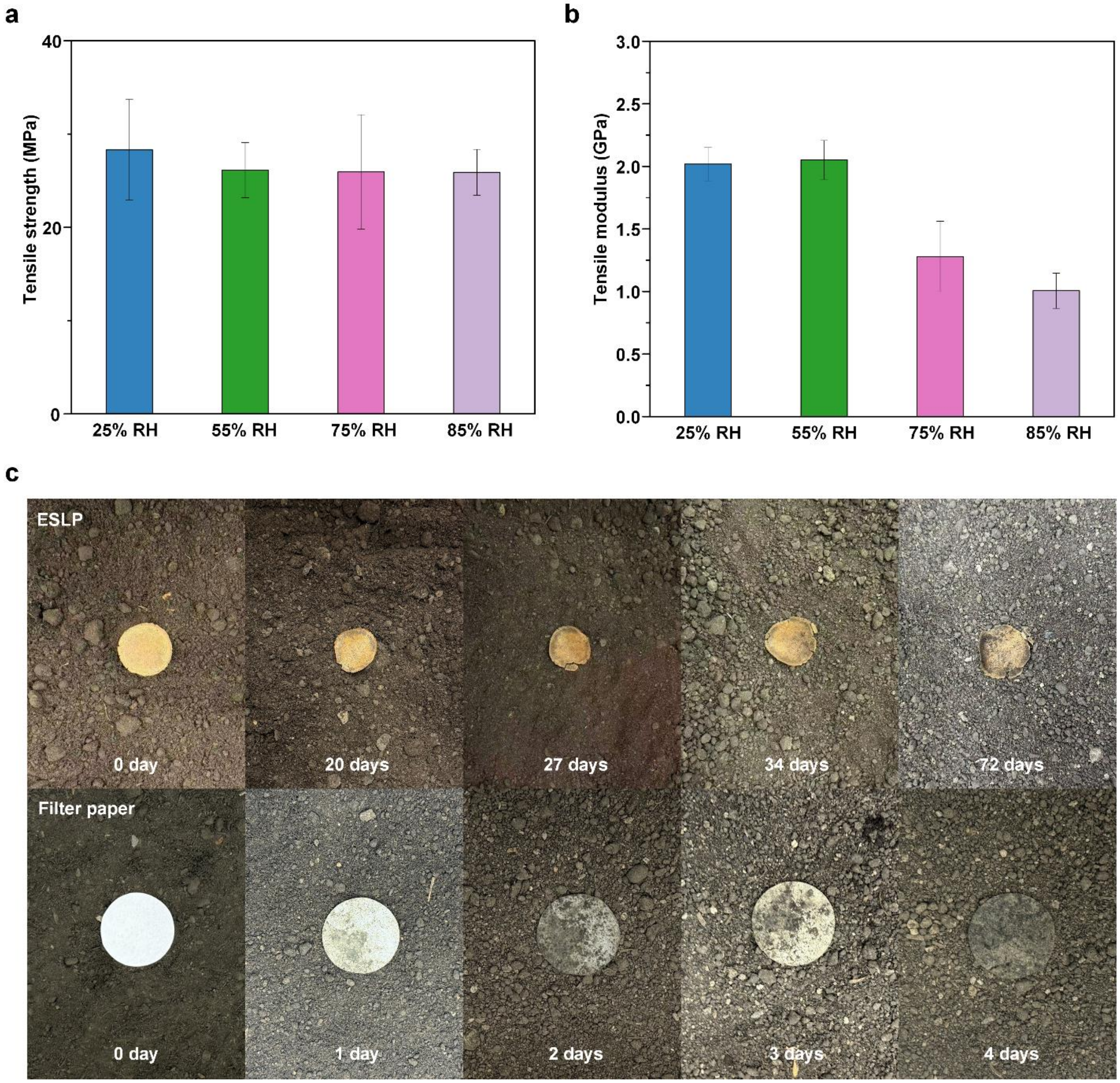

Fig. 2: Long-term humidity stability and composting degradation behavior of ESLP. a Tensile strength and **b** tensile modulus of ESLP after aging for 97 days at different relative humidities at room temperature. Error bars represent standard deviations. **c** Representative photographs showing the macroscopic disintegration of ESLP and filter paper during composting.

(3) A comprehensive performance comparison with commercial bio-based packaging materials is lacking. It is recommended to add a systematic comparison with existing mainstream biomass-based packaging materials (e.g., polylactic acid, cellulose-based composites) in terms of mechanical properties, thermal stability, gas permeability, and degradation performance, to more intuitively highlight the application advantages of the materials in this study.

Response: We thank the reviewer for this constructive suggestion. We agree that a systematic comparison with representative bio-based and conventional packaging materials is important for clarifying the practical positioning and application advantages of ESLP.

To address this point, we have added a comprehensive comparison with representative PLA, lignin-containing cellulose nanopaper, kraft paper and PET film in Table S7 of the Supporting Information. The comparison includes tensile strength, tensile modulus, wet or humidity stability, thermal behavior and degradation or end-of-life characteristics. Because the reported values were obtained using different formulations, conditioning procedures, specimen dimensions and testing standards, they are presented as representative literature benchmarks rather than as a strict performance ranking.

ESLP exhibited a tensile strength of 46.7 MPa and a tensile modulus of 2.5 GPa, placing its dry mechanical properties within the representative ranges reported for PLA, kraft paper and PET. Although the lignin-containing cellulose nanopaper showed substantially higher dry tensile strength, the principal advantage of ESLP lies in its moisture-resistant mechanical performance.

ESLP retained a wet tensile strength of 33.9 MPa and a wet tensile modulus of 2.0 GPa, corresponding to 72.6% and 80.0% retention, respectively. In comparison, the representative lignin-containing cellulose nanopaper and kraft paper retained approximately 32.5% and 4.1% of their dry tensile strengths under their reported wet-test conditions. Furthermore, after 97 days of aging at 25–85% RH, ESLP maintained tensile strengths of 25.9–28.3 MPa, demonstrating substantial long-term load-bearing capacity under prolonged humidity exposure.

The final ESLP composite showed a DTG maximum at 384.8 °C, which was higher than the representative values listed for PLA, lignin-containing cellulose nanopaper and kraft paper, although lower than that of PET. This result indicates that ESLP possesses sufficient thermal stability for many non-high-temperature packaging applications.

The degradation behavior was evaluated through complementary compost-burial and compost-respiration experiments. ESLP exhibited progressive surface deterioration, deformation and partial fragmentation during 72 days of compost burial. In addition, the blank-corrected cumulative CO_2 evolution reached 920.7 mg after 65 days, providing evidence of progressive microbial degradation under controlled composting conditions. Thus, ESLP combines service-relevant humidity stability with susceptibility to biological degradation under an appropriate end-of-life environment.

We respectfully acknowledge that gas permeability was not measured in the present study. The ESLP material developed here was primarily designed as a proof-of-concept platform to demonstrate covalent cellulose–lignin co-valorization and to improve the mechanical stability of

cellulose-based materials under wet and humid conditions. Its current fibrous architecture was not specifically engineered or sufficiently densified to form a continuous gas-barrier layer. Consequently, gas-permeability measurements at this stage would be strongly governed by the porosity, fiber packing, thickness and incomplete densification of the sheet, rather than solely reflecting the intrinsic barrier potential of the covalently bonded ESL–cellulose network. Direct comparison with dense commercial films such as PLA and PET could therefore be misleading because of the substantial differences in material architecture and intended function.

We sincerely apologize that reliable gas-permeability data could not be provided within the scope of the present revision, and we hope that the reviewer will understand this limitation. To avoid unsupported conclusions, gas permeability was not included in the quantitative comparison. We have instead explicitly acknowledged in the revised manuscript that gas-barrier performance remains an important parameter for future application-specific evaluation. Further development will require optimization of fiber packing, porosity, sheet thickness, surface continuity and potentially additional densification or coating strategies before oxygen, carbon dioxide and water-vapor permeability can be meaningfully assessed.

Overall, the comparison shows that the competitive advantage of ESLP lies in the combined balance of packaging-relevant mechanical strength, high wet-strength retention, long-term humidity stability, thermal stability and progressive degradation under composting conditions. The new comparison has been added as Table S7 in the Supporting Information, and the corresponding

discussion has been included in the revised manuscript, shown as below:

Page 17–18

To contextualize the packaging potential of ESLP, its performance was compared with representative PLA, lignin-containing cellulose nanopaper, kraft paper and PET film (Table S7). ESLP exhibited a tensile strength and modulus of 46.7 MPa and 2.5 GPa, respectively, which are within the representative ranges of widely used packaging materials. Its distinguishing feature is its moisture-resistant mechanical performance: ESLP retained 33.9 MPa wet tensile strength and a wet modulus of 2.0 GPa, corresponding to 72.6% and 80.0% retention, respectively. These strength-retention values are higher than those reported for the representative lignin-containing cellulose nanopaper and kraft paper included in the comparison. ESLP also showed a DTG maximum at 384.8 °C (Fig. S15) and retained tensile strengths of 25.9–28.3 MPa after 97 days of humidity aging. Together with its progressive macroscopic deterioration and blank-corrected CO_2 evolution under composting conditions, these results demonstrate a balance between service-stage moisture stability and end-of-life biological degradability.

SI

Table S4. Comparison of ESLP with representative bio-based and conventional packaging materials.

Material	Tensile strength (MPa)	Tensile modulus (GPa)	Wet or humidity stability	Thermal behavior	Degradation or end-of-life behavior	Ref.
ESLP	46.7	2.5	Wet tensile strength: 33.9 MPa with 72.6% retention; wet modulus: 2.0 GPa with 80.0% retention. After 97 days at 25–85% RH, tensile strength remained at 25.9–28.3 MPa.	DTG T_{max} at 384.8°C.	Progressive surface deterioration, deformation and partial fragmentation during 72 days of compost burial; blank-corrected cumulative CO_2 evolution reached 920.7 mg after 65 days.	This work
PLA, representative	58.2	1.05	NR under a directly comparable water-	Degradation onset: 211.9 °C; DTG T_{max}:	A representative PLLA formulation reached 100.2 ± 3.7% biodegradation	15,16

Material	Tensile strength (MPa)	Tensile modulus (GPa)	Wet or humidity stability	Thermal behavior	Degradation or end-of-life behavior	Ref.
neat film			immersion tensile test	324.8 °C	and complete disintegration under controlled industrial-composting conditions; compostable bags fully decomposed after seven weeks.	
Lignin-containing cellulose nanopaper	255	6.4	Wet tensile strength: 83 MPa, corresponding to approximately 32.5% retention	DTG T_{max}: 339 °C	NR in the cited study	[17]
Kraft paper, representative literature values	55.8	NR	Wet tensile strength: 2.3 MPa, corresponding to approximately 4.1% retention	Major thermal mass loss occurred at approximately 280–350 °C, with a T_{max} near 350 °C.	Paper products are biodegradable under composting conditions, although the degradation rate depends strongly on pulping history, lignin content and additives.	[18–20]
PET film	48.3–72.4	2.76–4.14	NR under a directly comparable water-immersion tensile test	DTG maximum: 441 °C	Not conventionally compostable; biological depolymerization generally requires specialized enzymes or microbial systems.	[21–23]

(4) The in-depth exploration of the reaction mechanism needs to be strengthened. Although XPS and FTIR confirm epoxide ring-opening and covalent bond formation, quantitative analysis of reaction kinetics (e.g., reaction rate constant, activation energy) is lacking. It is suggested to supplement reaction kinetic experiments at different temperatures to further improve the theoretical interpretation of the reaction mechanism.

Response: We thank the reviewer for this constructive suggestion. We agree that quantitative kinetic analysis is important for strengthening the mechanistic interpretation of the ESL–cellulose

reaction. To address this point, we performed additional time-resolved FTIR experiments at 140, 160 and 180 °C by monitoring the consumption of the epoxy-related band during curing. The integrated epoxy-band area was analyzed using an apparent pseudo-first-order model, giving apparent rate constants of 0.00755, 0.00865 and 0.00927 min^{-1} at 140, 160 and 180 °C, respectively. Fitting these FTIR-derived apparent rate constants according to an Arrhenius-type relationship yielded an apparent activation energy, E_a,app, of 8.03 kJ mol^{-1}, with an R^2 of 0.975. These results have been added to the revised manuscript and summarized in Table S5.

We further clarified the interpretation of this relatively low E_a,app in the revised text. Because the ESL–cellulose reaction occurs in a heterogeneous solid or softened-solid/solid interfacial system, the measured rate constants do not represent the intrinsic kinetics of a homogeneous elementary epoxide ring-opening reaction. Instead, the overall process is influenced by ESL softening and segmental mobility, interfacial wetting, local contact between epoxy groups and cellulose hydroxyls and the accessibility of hydroxyl groups within the rigid, hydrogen-bonded cellulose microfibrillar structure. Therefore, after the readily accessible interfacial epoxy groups have reacted, further conversion may become increasingly limited by contact, accessibility and restricted molecular mobility rather than solely by the chemical ring-opening barrier.

Accordingly, we now describe the kinetic results as apparent kinetic parameters and interpret E_a,app as a semi-quantitative descriptor of the overall ESL–cellulose interfacial curing process. We also added relevant references on solid-state reactions, cellulose structure and accessibility,

cellulose etherification and complex epoxy curing systems to support this interpretation. The corresponding discussion has been included in the revised manuscript, shown as below:

Page 10-11

Quantitative analysis of the integrated epoxy band using an apparent pseudo-first-order model gave apparent rate constants of 0.00755, 0.00865 and 0.00927 min^{-1} at 140, 160 and 180 °C, respectively (Table S5). Fitting the FTIR-derived apparent rate constants according to an Arrhenius-type relationship yielded an apparent activation energy, E_a,app, of 8.03 kJ mol^{-1}, with an R^2 of 0.975. The relatively low E_a,app primarily reflects the modest temperature dependence of the averaged FTIR-derived apparent rate constants over the investigated temperature range. This value should be interpreted in the context of the heterogeneous ESL–cellulose interfacial reaction rather than as the intrinsic activation barrier of an elementary epoxide ring-opening reaction. In this solid or softened-solid/solid system, epoxide groups on ESL and hydroxyl groups on cellulose are confined to different phases. Bond formation therefore requires ESL softening and segmental mobility, interfacial wetting, local contact between reactive groups as well as accessibility of cellulose hydroxyls. Solid-state reactions are often affected by precursor architecture, interfacial contact and transport within solid phases, while cellulose possesses a rigid, hydrogen-bonded microfibrillar structure in which hydroxyl groups are not equally mobile or accessible[58–60]. Increasing temperature can therefore promote early-stage epoxide conversion, ESL mobility, wetting and fiber–fiber interfacial contact, facilitating the formation of a more continuous load-bearing network during the fixed curing period. However, once the readily accessible interfacial epoxy groups have reacted, further conversion may become increasingly limited by contact, accessibility and restricted molecular mobility rather than solely by the chemical ring-opening barrier. Previous studies of solid-state reactions, cellulose structure and accessibility, cellulose etherification and complex epoxy curing systems have similarly emphasized the roles of interfacial contact, restricted mobility and hydroxyl accessibility[61–64]. Therefore, E_a,app is regarded as a semi-quantitative apparent descriptor of the overall ESL–cellulose interfacial curing process, rather than a true activation energy for a single elementary reaction.

SI

Table S5. Apparent kinetic parameters for epoxy consumption during the ESL–cellulose reaction.

Curing temperature (°C)	Apparent rate constant (k_{app}) (min^{-1})	Uncentered (R^2)
140	0.00755	0.899
160	0.00865	0.974
180	0.00927	0.844

Arrhenius analysis of the obtained rate constants gave an apparent activation energy, $E_{a,app}$, of 8.03 kJ mol^{-1}, with an R^2 of 0.975.

3. Data Quality and Result Analysis.

(1) The presentation of some characterization data is not detailed enough. For example, in GPC analysis, specific values of number-average molecular weight (Mn), weight-average molecular weight (Mw), and dispersity (Đ) are not provided, and only the molecular weight change is explained through curve trends. It is recommended to supplement specific data to enhance persuasiveness; the assignment of some characteristic peaks in FTIR spectra (e.g., the C-H vibration peak of the epoxy ring around 3056 cm-1) can be further supported by combining with literature data.

Response: We thank the reviewer for this careful and constructive comment. We agree that reporting the quantitative molecular-weight parameters and providing literature-supported FTIR assignments improve the clarity and persuasiveness of the structural characterization.

To address the GPC comment, we have added the number-average molecular weight (M_n), weight-average molecular weight (M_v), z-average molecular weight (M_z) and dispersity (Đ) of the

two apparent molecular-weight fractions of SL and ESL in Table S4 of the Supporting Information. For the higher-molecular-weight fraction, M_n and M_v increased from 1431 and 1521 Da for SL-1 to 1967 and 2234 Da for ESL-1, respectively. For the lower-molecular-weight fraction, M_n and M_v increased from 476 and 572 Da for SL-2 to 678 and 725 Da for ESL-2, respectively. The corresponding dispersities remained relatively narrow, changing from 1.06 to 1.14 for the higher-molecular-weight fraction and from 1.20 to 1.07 for the lower-molecular-weight fraction. These quantitative results confirm that epoxy functionalization shifted both apparent molecular-weight fractions toward higher molecular weights without causing pronounced broadening of the molecular-weight distributions.

We have also revised the FTIR discussion and added literature-supported assignments of the epoxy-related bands. The weak shoulder at approximately 3056 cm^{-1} is assigned to the C–H stretching vibration of the oxirane ring, while the band at approximately 908 cm^{-1} is assigned to the characteristic epoxide-ring vibration. The simultaneous appearance of these bands, together with the attenuation of the broad O–H stretching band, provides additional evidence for successful conversion of the phenolic hydroxyl groups of SL into epoxy functionality.

The quantitative GPC data have been added as Table S4, and the corresponding GPC and FTIR discussions, together with the relevant references, have been included in the revised manuscript, shown as below:

Page 6–7

Gel permeation chromatography (GPC) analysis revealed a shift of both apparent molecular-weight fractions toward higher molecular weights after epoxy functionalization (**Error! Reference source not found.**e, Table S4). For the higher-molecular-weight fraction, M_n and M_v increased from 1431 and 1521 Da for SL-1 to 1967 and 2234 Da for ESL-1, respectively. For the lower-molecular-weight fraction, M_n and M_v increased from 476 and 572 Da for SL-2 to 678 and 725 Da for ESL-2, respectively. The dispersity remained relatively narrow for all fractions (Đ = 1.06–1.20), indicating that epoxy functionalization increased the apparent molecular size without causing pronounced broadening of the molecular-weight distribution.

Page 7

Fourier transform infrared (FTIR) spectroscopy spectra further corroborated these assignments (**Error! Reference source not found.**f). Relative to SL, ESL shows attenuation of the broad O–H stretch near 3400 cm^{-1} and emergence of a characteristic epoxide band at 908 cm^{-1}, consistent with epoxide formation and with the concomitant decrease in phenolic hydroxyl content observed by ^{31}P NMR[50,51]. The tiny shoulder around 3056 cm^{-1} represent the C–H vibration of epoxide ring, which support the successfully of epoxy-functionalization as well[52,53].

Page 10

Kinetic FTIR analysis was employed to analyze the reaction process between cellulose and ESL, and the spectra reveal time-dependent changes in the chemical composition of ESLFP and ESLFP* heated for different durations (**Error! Reference source not found.**c–d). As heating proceeded, the epoxy band of ESLFP at 908 cm^{-1} in the ESLFP sample gradually decreased in intensity and red-shifted towards 898 cm^{-1}, indicating consumption of epoxy groups and the re-emergence of the β–1,4–glycosidic bond vibration of cellulose[50,51,57].

Page 12–13

FTIR was used to assess bulk chemical changes since XPS probes only the near surface chemical compositions. Neat ESL displays the epoxide ring vibration at 908 cm^{-1}, whereas FP, ESLFP* and ESLFP show a band at 896 cm^{-1} assigned to the cellulose β–1,4–glycosidic linkages[50,51,57]. In the DMAP-cured composite (ESLFP), the disappearance of the 908 cm^{-1} epoxide band—together with the persistent 896 cm^{-1} cellulose band—indicates epoxide ring-opening and formation of new ether linkages between ESL and cellulose (Fig. S7)[50,51,57].

SI

Table S6. GPC-derived molecular-weight characteristics of the sharp high-molecular-weight fraction and broad lower-molecular-weight fraction.

Sample	Retention time (min)	Mn (Da)	Mw (Da)	Mz (Da)	Đ (Mw/Mn)
SL-1	18.5 – 22.0	1431	1521	1628	1.06
SL-2	22.0 – 25.5	476	572	649	1.20
ESL-1	17.5 – 21.7	1967	2234	2579	1.14
ESL-2	21.7 – 24.5	678	725	772	1.07

(2) The analysis of the influence mechanism of ash on the reaction process is not in-depth enough. Although the technology is mentioned to be tolerant to high ash content, the existing form of ash (e.g., alkali metal and alkaline earth metal compounds) in the reaction and its specific influence on reactive fractionation, epoxy functionalization, and other links (e.g., whether it participates in the reaction, whether it affects product purity) are not clarified. It is recommended to supplement relevant characterizations (e.g., ICP-OES analysis of ash element composition, XRD analysis of ash phase) and control experiments to clarify the internal reason for ash tolerance.

Response: We thank the reviewer for this insightful comment. We agree that further clarification of the ash-related inorganic components is important for supporting the discussion on feedstock tolerance. To address this point, we supplemented XRD analysis of the ash residue obtained from rapeseed straw and added an ash-mimic control experiment using representative inorganic salts.

The ash XRD pattern showed several crystalline diffraction peaks, indicating that the ash residue contained inorganic salt/mineral phases rather than being fully amorphous. Based on the

observed peak positions and reported mineral phases in biomass ashes, the major reflections were tentatively assigned to SiO_2-related phases, K-containing salts and Ca-containing mineral phases (Fig. S12a). Because biomass ash is a complex multiphase inorganic residue and several diffraction peaks overlap, the assignments are made conservatively and are intended to identify representative inorganic components rather than definitive single crystalline phases for every peak.

To further evaluate whether these ash-related inorganic salts interfere with the reactive fractionation, we performed an ash-mimic fractionation experiment by adding KCl and $CaCl_2$ (3 wt% of biomass) to poplar before fractionation. The ash-mimic system gave a comparable SL yield of 35.5% and a pulp purity of 96.7%, close to the optimized salt-free condition, which gave an SL yield of 35.8% and a pulp purity of 94.2% (Table S3). These results indicate that the representative K- and Ca-containing salts did not substantially inhibit the syringol-assisted reactive fractionation under the tested conditions.

The ash tolerance of the present process is therefore attributed primarily to its metal-free nature. In conventional metal-catalyzed fractionation routes, alkali and alkaline-earth inorganic species can poison, foul, or alter metal catalyst surfaces. In contrast, the present reactive fractionation does not rely on transition-metal catalytic sites. As a result, the observed inorganic salt/mineral phases are less likely to fundamentally deactivate the main fractionation pathway, although their influence on phase distribution, washing efficiency and residual inorganic impurities may require more detailed study in future work. We have added the ash XRD data as Fig. S12a, the ash-mimic control

to Table S3, and the corresponding discussion to the revised manuscript and Supporting Information, shown as below:

Page 13

XRD analysis of the rapeseed-straw ash indicated representative K- and Ca- containing inorganic phases (Fig. S12a, ST. 2). Based on that, an ash-mimic experiment also gave an SL yield of 35.5% and pulp purity of 96.7%, comparable to the salt-free condition (ST. 3).

SI

ST. 2: XRD analysis of ash from rapeseed straw

To better understand the inorganic components responsible for the ash content, XRD analysis was performed on the ash residue from rapeseed straw (Fig. S12a). The pattern showed crystalline diffraction features that were tentatively assigned to SiO_2-related phases, K-containing salts, Ca-containing mineral phases and overlapping K/Ca-related inorganic phases, consistent with reported mineral phases in herbaceous biomass ashes[5–8]. Because biomass ash is a complex multiphase residue, these assignments are used to identify representative inorganic components rather than definitive single crystalline phases for every peak.

ST. 3: Ash mimic fractionation experiment

An ash-mimic fractionation experiment was performed by adding representative K and Ca salts (3 wt% of biomass) to poplar. The ash-mimic system gave a comparable SL yield of 35.5% and pulp purity of 96.7% (Table S3), close to the optimized salt-free condition, indicating that these inorganic salts did not substantially inhibit syringol-assisted fractionation under the tested conditions.

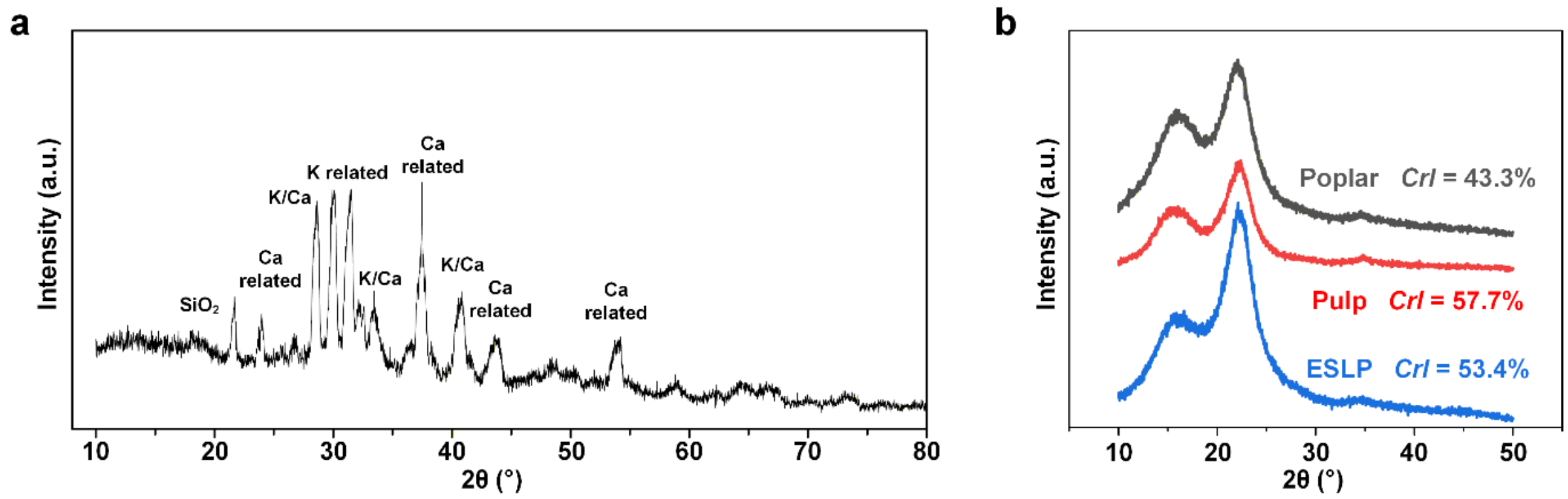

Fig. S12: XRD analysis of cellulose crystallinity and rapeseed straw ash. a XRD pattern of rapeseed straw ash. The major diffraction features are tentatively assigned to SiO_2-related phases, K-related salts, Ca-related phases and overlapping K/Ca-containing inorganic phases. **b** XRD patterns of native poplar, fractionated pulp and ESLP.

Table S7. Optimization of syringol assisted reactive fractionations.

Sample	Poplar/syringol (wt/wt)	SL yield (%)	Pulp yield (%)	Pulp purity (%)
SL-0.2	1:0.2	34.0	49.4	95.6
SL-0.4	1:0.4	35.8	46.9	94.2
SL-0.6	1:0.6	37.7	46.7	96.5
Ash mimic	1:0.4	35.5	44.6	96.7

Appendixes

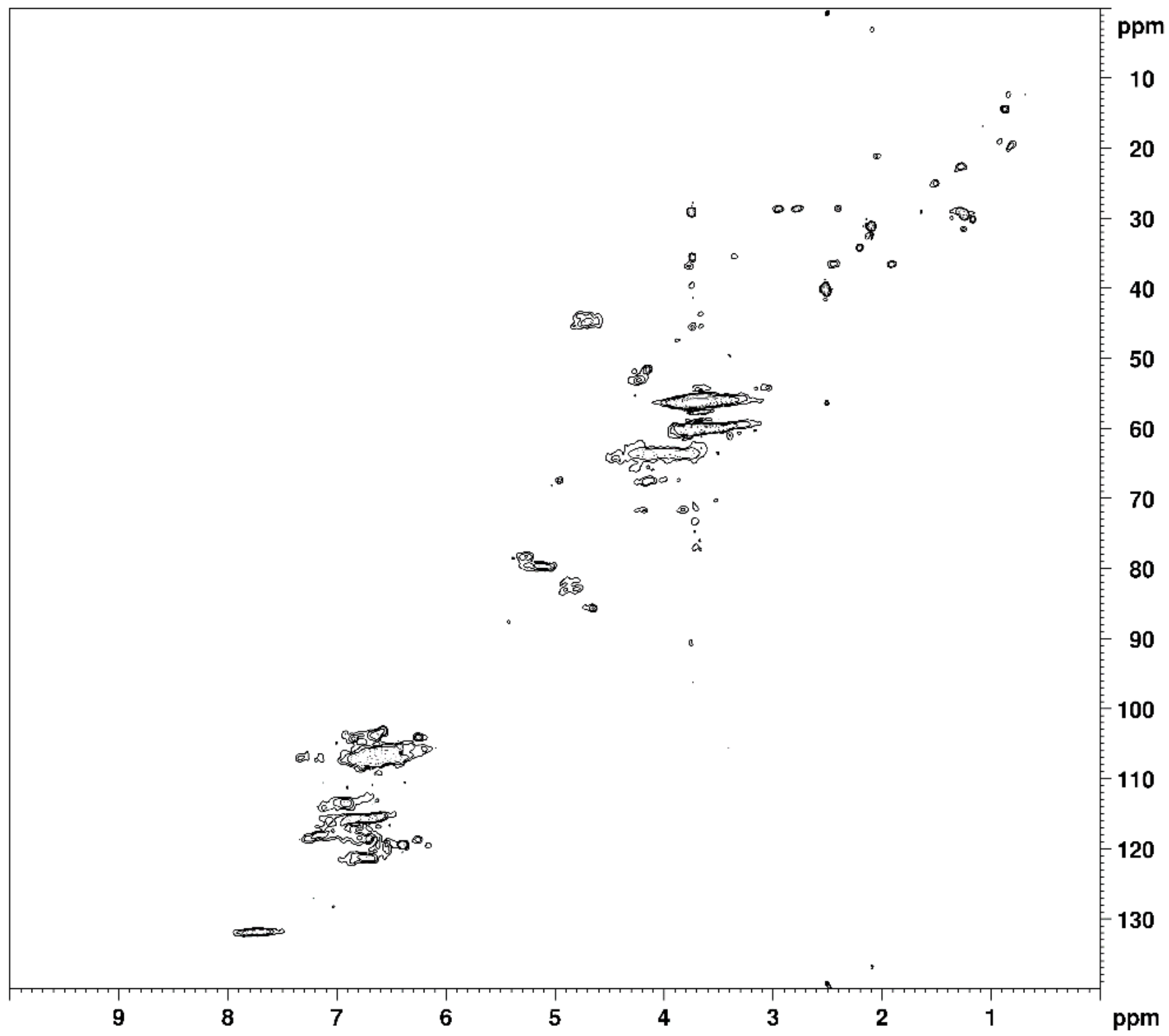

^{1}H–^{13}C HSQC NMR spectrum of SL from ash mimic experiment

(3) The degradation performance of the composites is not involved. As a potential packaging material, biodegradability is an important evaluation index. It is recommended to supplement degradation experiments of the materials under natural environment or composting conditions to investigate their environmental friendliness and improve the performance evaluation system of the materials.

Response: We thank the reviewer for this valuable suggestion. We agree that degradation performance is an important parameter for evaluating the environmental applicability of ESLP as a potential packaging material. To address this point, we supplemented two complementary composting experiments: a compost-burial test to monitor macroscopic disintegration and a

compost-respiration test to quantify CO_2 evolution associated with microbial degradation.

In the compost-burial experiment, filter paper was used as a cellulose-based positive control. The filter paper underwent rapid macroscopic deterioration within five days, confirming the biological activity of the compost. In comparison, ESLP showed a slower but progressive degradation process, including surface discoloration, deformation, erosion and partial fragmentation over 72 days (Fig. 7c). The slower physical disintegration of ESLP is consistent with the presence of lignin and the covalently coupled ESL–cellulose network, which provide greater structural stability than unmodified cellulose.

Biological degradation was further evaluated in a separate compost-respiration experiment by monitoring cumulative net CO_2 evolution after subtraction of the compost blank. The cumulative CO_2 evolution increased progressively during incubation and reached 920.7 mg after 65 days (Fig. S14). The sigmoidal evolution profile showed an initially slower stage, followed by accelerated CO_2 production and a subsequent tendency toward a plateau, indicating progressive microbial conversion of material-derived organic carbon into CO_2. The relatively high cumulative CO_2 value may partly reflect the short-term rate extrapolation and possible substrate-induced priming of native compost organic matter; therefore, it is interpreted as blank-corrected respiration associated with ESLP addition rather than an absolute value derived exclusively from ESLP carbon.

The two experiments provide complementary information: the burial test demonstrates macroscopic disintegration, whereas the CO_2-evolution experiment provides evidence of microbial

degradation. Together, the results show that ESLP retains greater structural integrity than filter paper during the initial composting period but remains susceptible to progressive microbial degradation under biologically active composting conditions. The compost-burial photographs have been added as Fig. 7c in the revised manuscript, while the cumulative CO_2-evolution results have been added as Fig. S14 in the Supporting Information. The corresponding experimental procedures and discussion have also been included in the revised manuscript and Supporting Information, shown as below:

Page 16

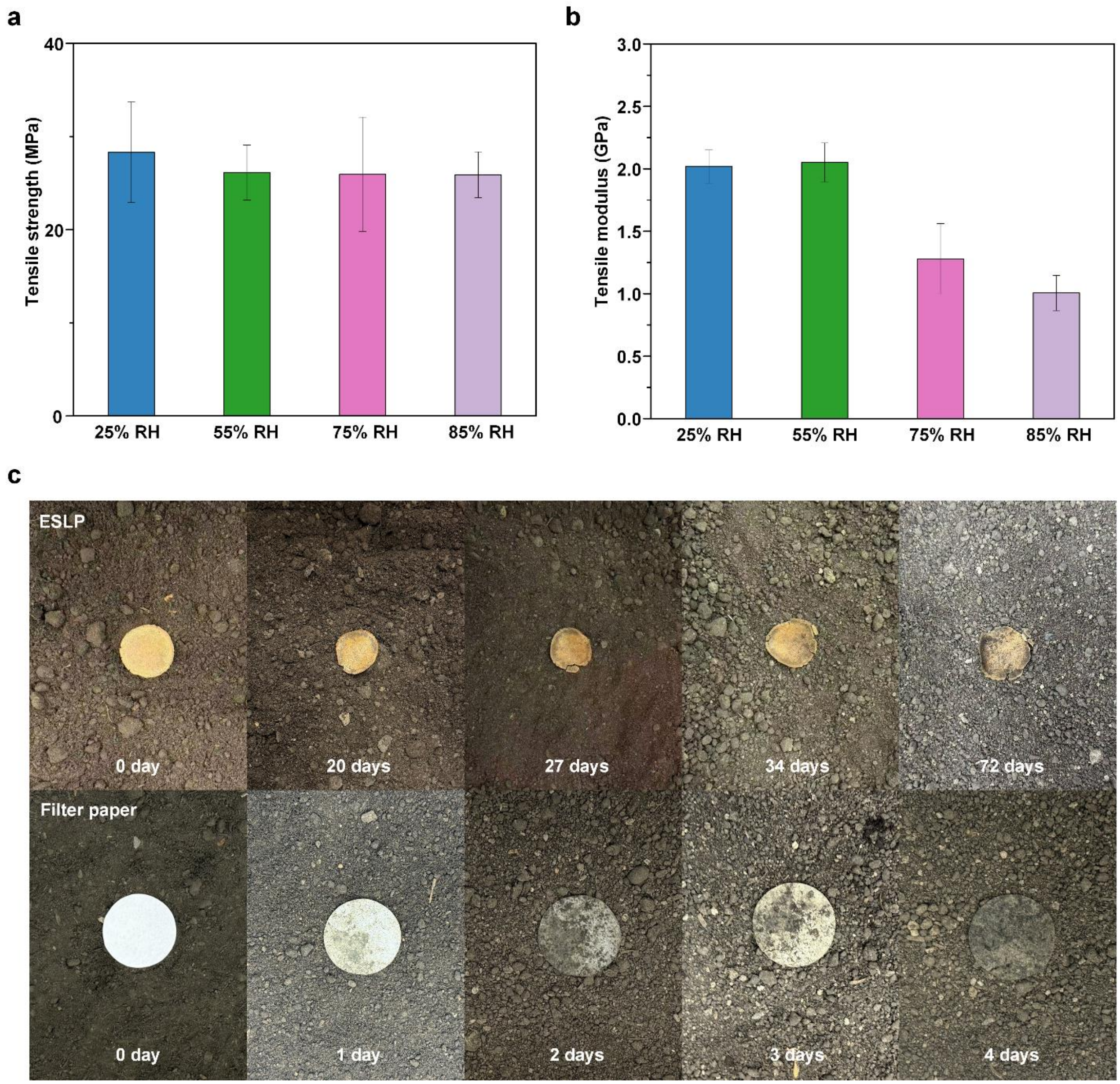

Fig. 3: Long-term humidity stability and composting degradation behavior of ESLP. a Tensile strength and **b** tensile modulus of ESLP after aging for 97 days at different relative humidities at room temperature. Error bars represent standard deviations. **c** Representative photographs showing the macroscopic disintegration of ESLP and filter paper during composting.

Page 17

Having established that ESLP reached a stable moisture-sorption state while retaining substantial mechanical integrity during prolonged humidity exposure, we next examined whether this service-stage durability could coexist with biological degradation under an appropriate end-of-life environment. In the compost-burial experiment, the filter-paper control underwent rapid

macroscopic deterioration within four days and was no longer visually detectable after five days, whereas ESLP showed gradual surface discoloration, deformation, erosion and partial fragmentation over 72 days (Fig. 2c). The slower disintegration of ESLP is consistent with its lignin-containing and covalently coupled network. In a separate compost-respiration experiment, the blank-corrected cumulative CO_2 evolution increased progressively and reached 920.7 mg after 65 days (Fig. S14), indicating enhanced microbial respiration associated with ESLP addition. Because the measurement was based on extrapolation of a 2 h respiration rate to a long-term equivalent, and the ESLP addition may stimulate the decomposition of native compost organic matter, the cumulative value is not interpreted as an absolute value derived exclusively from ESLP carbon[65–67]. Thus, the burial and respiration experiments provide complementary evidence of macroscopic disintegration and biological degradation, respectively. These results indicate that ESLP can maintain functional integrity under service-relevant humidity conditions while undergoing progressive degradation under an appropriate composting environment.

SI

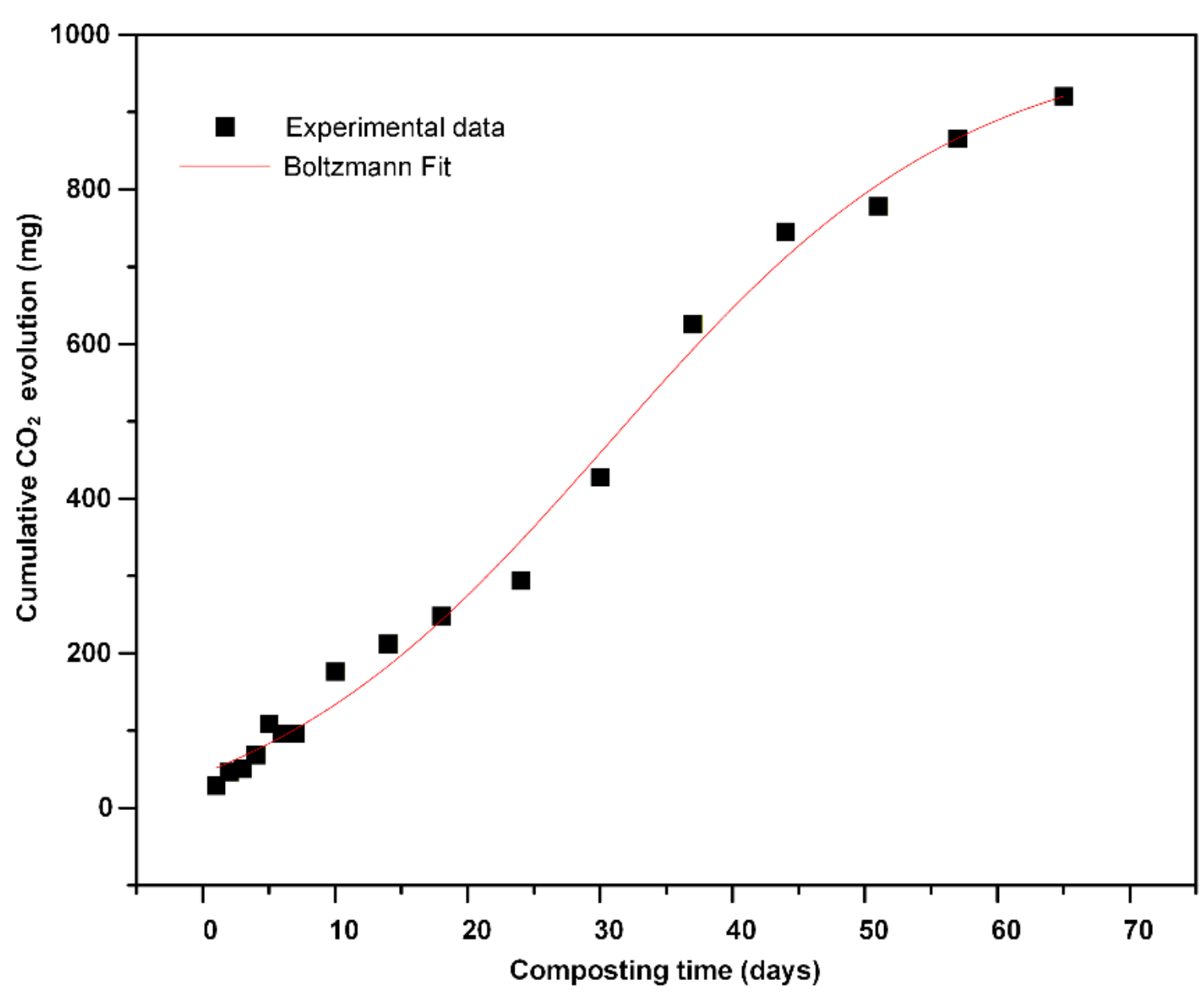

Model	Boltzmann
Equation	y = A2 + (A1-A2)/(1 + exp((x-x0)/dx))
Plot	Cumulative CO2 evolution
A1	-53.60869 ± 57.53754
A2	996.96416 ± 58.95123
x0	30.64351 ± 1.65377
dx	13.51516 ± 2.31862
Reduced Chi-Sqr	836.54621
R-Square (COD)	0.99331
Adj. R-Square	0.99177

Fig. S14: Cumulative CO_2 evolution from ESLP during compost incubation. Cumulative net CO_2 evolution during 65 days of composting after subtraction of the compost blank. The symbols represent the experimental data, and the line represents an empirical sigmoidal fit.

4. Conclusions and Discussion

(1) The comparison between this technology and "lignin-first" strategies (e.g., reductive catalytic fractionation, RCF) in the discussion is not comprehensive enough. It is recommended to conduct a systematic comparison from aspects of feedstock adaptability, process complexity, product added value, and industrialization cost to further highlight the competitive advantages of this technology.

Response: We thank the reviewer for this constructive suggestion. We agree that a more systematic comparison with lignin-first strategies, particularly reductive catalytic fractionation (RCF), would help clarify the positioning and practical advantages of the present method. To address this point, we have added a comparison table in the Supporting Information and revised the manuscript discussion around the rapeseed straw experiment.

The revised comparison clarifies that the present strategy and RCF have different product targets and should be viewed as complementary rather than directly competing technologies. RCF is a powerful lignin-first strategy for producing lignin-derived aromatic monomers and oligomers. However, it commonly requires transition-metal catalysts, reductive conditions, catalyst recovery and downstream separation or upgrading of lignin-derived products. These requirements can complicate the processing of ash-rich residual feedstocks, because alkali and alkaline-earth metal species may poison or foul metal catalysts.

In contrast, the present strategy is metal-free and operates under ambient pressure. Rather than depolymerizing lignin into monomers, syringol-assisted reactive fractionation directly introduces a chemical handle into the lignin stream, which can then be converted into epoxy-functionalized

syringolated lignin and recombined with cellulose into a covalently bonded composite. Therefore, the key advantage of this approach lies in direct cellulose–lignin co-valorization into a wet-stable material, particularly from residual biomass streams with elevated ash contents.

We have also revised the text to provide a more balanced discussion of industrialization considerations. While this method avoids transition-metal catalysts and external hydrogen, further process development is still required to improve epichlorohydrin use or recycling, product separation and hot-pressing energy efficiency. The new comparison has been added as Table S6 in the Supporting Information, and the corresponding discussion has been included in the revised manuscript, shown as below:

Page 13

RCF is highly effective for producing lignin-derived aromatic monomers or oligomers, but typically relies on transition-metal catalysts, reductive conditions, catalyst recovery and downstream product separation or upgrading. These requirements can be challenging for ash-rich residual feedstocks because alkali and alkaline-earth metal species may poison or foul metal catalysts. In contrast, the present metal-free process does not rely on surface metal catalytic sites and directly yields a reactive lignin stream and cellulose pulp that can be recombined into a covalently bonded composite. To demonstrate that reactive fractionation is feasible for ash enriched feedstocks, we applied the methodology to rapeseed straw, which contains 2% of ashes. The process afforded SL in a yield of 24.8% based on dry biomass and a paler off-white solid ESL (Fig. S8–9). The resulting composite reached tensile strengths of 40.0 MPa in the dry state and 21.8 MPa under wet conditions, compared with 11.9 and 1.5 MPa, respectively, for the corresponding neat pulp (Fig. S11, ST. 1). XRD analysis of the rapeseed-straw ash indicated representative K- and Ca-containing inorganic phases (Fig. S12a, ST. 2). Based on that, an ash-mimic experiment also gave an SL yield of 35.5% and pulp purity of 96.7%, comparable to the salt-free condition (ST. 3).

The successful use of rapeseed straw also highlights a key distinction between the present strategy and conventional lignin-first approaches such as RCF (Table S6). Thus, this strategy is complementary to RCF: rather than targeting aromatic monomer production, it provides a material-oriented route for direct cellulose–lignin co-valorization from residual biomass streams.

Table S6. Comparison between the present reactive fractionation strategy and representative lignin-first/RCF strategies[9–14].

Aspect	Present work: syringol-assisted reactive fractionation	Representative lignin-first / RCF strategies
Main product target	Reactive syringolated lignin and cellulose pulp, recombined into covalently bonded lignin–cellulose composites	Lignin-derived aromatic monomers/oligomers, with carbohydrate-rich pulp as a coproduct
Product strategy	Material-oriented cellulose–lignin co-valorization without complete lignin depolymerization	Monomer-/chemical-oriented lignin depolymerization and stabilization
Feedstock adaptability	Metal-free system; suitable for ash-containing residual feedstocks such as rapeseed straw and ash-mimic poplar	Often sensitive to ash-rich feedstocks because inorganic salts may interfere with catalyst performance and recovery
Catalyst and reductive conditions	No transition-metal catalyst or external H_2 required	Commonly requires heterogeneous transition-metal catalysts and H_2 or hydrogen-donor systems
Process complexity	Fractionation directly installs a reactive handle on lignin; subsequent epoxy-functionalization enables direct composite fabrication	Requires catalyst recovery, lignin oil separation and often further downstream upgrading/polymerization before material use
Solvent and separation considerations	Excessive formic acid and syringol are recyclable.	Solvent loading, liquor recycling and solvent recovery are important cost and energy drivers in RCF scale-up
Product added value	Wet-stable bio-based composite with high wet-strength retention	Aromatic monomers/oligomers or polymer precursors; value depends on downstream separation, upgrading and application development
Industrialization	Advantages: metal-free, ambient	Advantages: established lignin-first

Aspect	Present work: syringol-assisted reactive fractionation	Representative lignin-first / RCF strategies
considerations	pressure, direct material formation. Challenges: epichlorohydrin use/recycling, solvent handling and hot-pressing energy	chemistry and aromatic chemical production. Challenges: catalyst cost/recovery, H_2 supply, solvent recycling and monomer separation/upgrading
Overall positioning	Complementary material-oriented route for direct cellulose–lignin co-valorization, especially from residual streams	Powerful monomer-oriented lignin-first route for lignin valorization

(2) The discussion on the expansion of application scenarios for the composites is insufficient. The existing discussion mainly focuses on packaging materials. It is recommended to explore their potential applications in other fields (e.g., biomedical materials, agricultural mulch films, water treatment membranes) combined with the performance characteristics of the materials (e.g., wet stability, mechanical strength), so as to broaden the application perspective of the research.

Response: We thank the reviewer for this helpful suggestion. We agree that the application perspective of ESLP should be broadened beyond packaging materials. In the revised manuscript, we have added a brief application-outlook paragraph before the Discussion section, following the mechanical, humidity-aging and composting-degradation results.

Based on its mechanical strength, wet-strength retention, long-term humidity stability and progressive degradation under composting conditions, ESLP may also be relevant to wet-resistant molded fiber products, disposable bio-based articles and agricultural materials that require

temporary mechanical integrity during use and degradation after disposal. We have also mentioned filtration supports and water-treatment membrane substrates as possible future directions because the covalently bonded lignin–cellulose network provides dimensional stability in wet environments.

At the same time, we have avoided overstating unsupported applications. More demanding fields such as water-treatment membranes and biomedical-related materials would require additional application-specific evaluations, including porosity control, permeability, separation performance, cytocompatibility, sterilization stability and safety assessment. Therefore, these applications are discussed as future opportunities rather than demonstrated uses of the present material.

The corresponding discussion has been included in the revised manuscript, shown as below:

Page 17

Taken together, these results demonstrate that ESLP combines service-stage mechanical and moisture stability with end-of-life degradability under composting conditions, providing a useful performance basis for applications beyond conventional packaging. Beyond packaging, the combination of mechanical strength, wet-strength retention, long-term humidity stability and composting degradability suggests that ESLP may serve as a broader wet-stable bio-based materials platform. For example, the retained load-bearing capacity under humid conditions could be useful for molded fiber products, wet-resistant disposable articles and agricultural materials that require temporary mechanical integrity during use but eventual degradation after disposal. The covalently bonded lignin–cellulose network may also provide a basis for future filtration supports or water-treatment membrane substrates where dimensional stability in wet environments is required. However, such applications would require additional control over porosity, permeability, separation performance, and, for biomedical-related uses, cytocompatibility and safety. These directions are therefore regarded as future opportunities rather than demonstrated applications in the present study.

(3) The prospect of industrialization of the technology in the conclusion section is too general. It is recommended to supplement the analysis of potential challenges in the industrialization process (e.g., raw material pretreatment, product separation and purification, cost control) and propose preliminary solutions to enhance the practical application guiding significance of the research.

Response: We thank the reviewer for this constructive suggestion. We agree that the industrialization perspective should be discussed more specifically. In the revised manuscript, we have added a concise analysis of the main practical challenges, including feedstock variability, solvent and reagent recovery, product separation, epichlorohydrin use and hot-pressing energy consumption.

Potential solutions include feedstock screening and size control, closed-loop recovery of formic acid and excess syringol, improved filtration and washing processes, recycling or replacement of epichlorohydrin and optimization of catalyst loading and curing conditions to reduce hot-pressing time and energy demand. We have also clarified that, although the metal-free and ambient-pressure process provides a promising basis for scale-up, further process optimization and techno-economic assessment are required before industrial implementation. The corresponding discussion has been included in the revised manuscript, shown as below:

Page 17

Several practical challenges remain before industrial implementation. Feedstock variability may require screening, drying, size control and compositional monitoring. Efficient recovery of epichlorohydrin, together with improved filtration and washing, will be important for reducing

reagent consumption and separation costs. In addition, the current hot-pressing condition should be optimized by shortening the curing time, tuning catalyst loading and improving heat transfer. Thus, although the metal-free, ambient-pressure process provides a promising basis for scale-up, further process optimization and techno-economic assessment are required.

Reviewer #2:

Dear Authors

It's interesting and new approach of making composite materials by combining lignin with cellulose via epichlorohydrin (ECH), which deserves its publication. However there is a few points to clarified prior to its publication with minor revision:

Response: We sincerely thanks the reviewer's recommendation.

1. It is necessary to provide details of preventing recondensation (or self-condensation) of lignin in the isolation process in relation with using formic acid.

Response: We thank the reviewer for this important comment. We agree that lignin recondensation or self-condensation should be carefully discussed in relation to the use of formic acid, and we apologize that this point was not sufficiently clarified in the original manuscript.

In the present system, suppression of uncontrolled lignin recondensation is achieved by adding syringol during the formic-acid-based fractionation. Under acidic formic acid conditions, cleavage of β–O–4 linkages can generate reactive benzylic Cα intermediates. In the absence of an external nucleophile, these intermediates can undergo acid-catalyzed hydrolysis and undesirable lignin–lignin condensation, leading to significant structural changes of the lignin polyaromatic network. This behavior has been demonstrated in the reported syringol/formic-acid fractionation system,

where lignin obtained using formic acid alone retained negligible β-ether units due to acidic hydrolysis and undesirable condensation reactions. In contrast, the presence of syringol directs the reaction toward selective α-arylation, thereby trapping the reactive Cα intermediate and preventing uncontrolled self-condensation. To further support this mechanism in our own system, we supplemented an FA-only fractionated lignin control and analyzed it by HSQC NMR. Compared with SL obtained from syringol-assisted reactive fractionation, the FA-only lignin did not show the diagnostic Cα-syringolated correlations. Instead, its HSQC spectrum showed negligible β–O–4 correlations, consistent with acid-induced hydrolysis and non-selective lignin condensation in the absence of syringol. By contrast, SL exhibited clear substituted Cα correlations, while the native Cα–OH signal disappeared, confirming that syringol selectively intercepted the Cα intermediate to form syringolated lignin.

We have revised the manuscript to clarify that syringol does not simply "avoid" all condensation; rather, it redirects the acid-induced reactive intermediate toward controlled α-arylation, thereby suppressing undesirable lignin self-condensation. The FA-only lignin HSQC spectrum has been added as Figure S5, and the corresponding discussion has been included in the revised manuscript, shown as below:

Page 6

Mechanistically, formic-acid-mediated cleavage of β–O–4 linkages generates reactive benzylic Cα intermediates that can undergo acid-induced hydrolysis and undesirable lignin condensation in the absence of an external trapping reagent[42]. The FA-only fractionated lignin retained negligible β-ether units because of acidic hydrolysis and non-selective condensation,

whereas syringol directs the reaction toward selective α-arylation (Fig. S5). In contrast, SL displayed clear substituted Cα correlations together with disappearance of the native Cα–OH signal, confirming that syringol selectively trapped the reactive Cα intermediate and suppressed uncontrolled lignin self-condensation (**Error! Reference source not found.**a).

SI

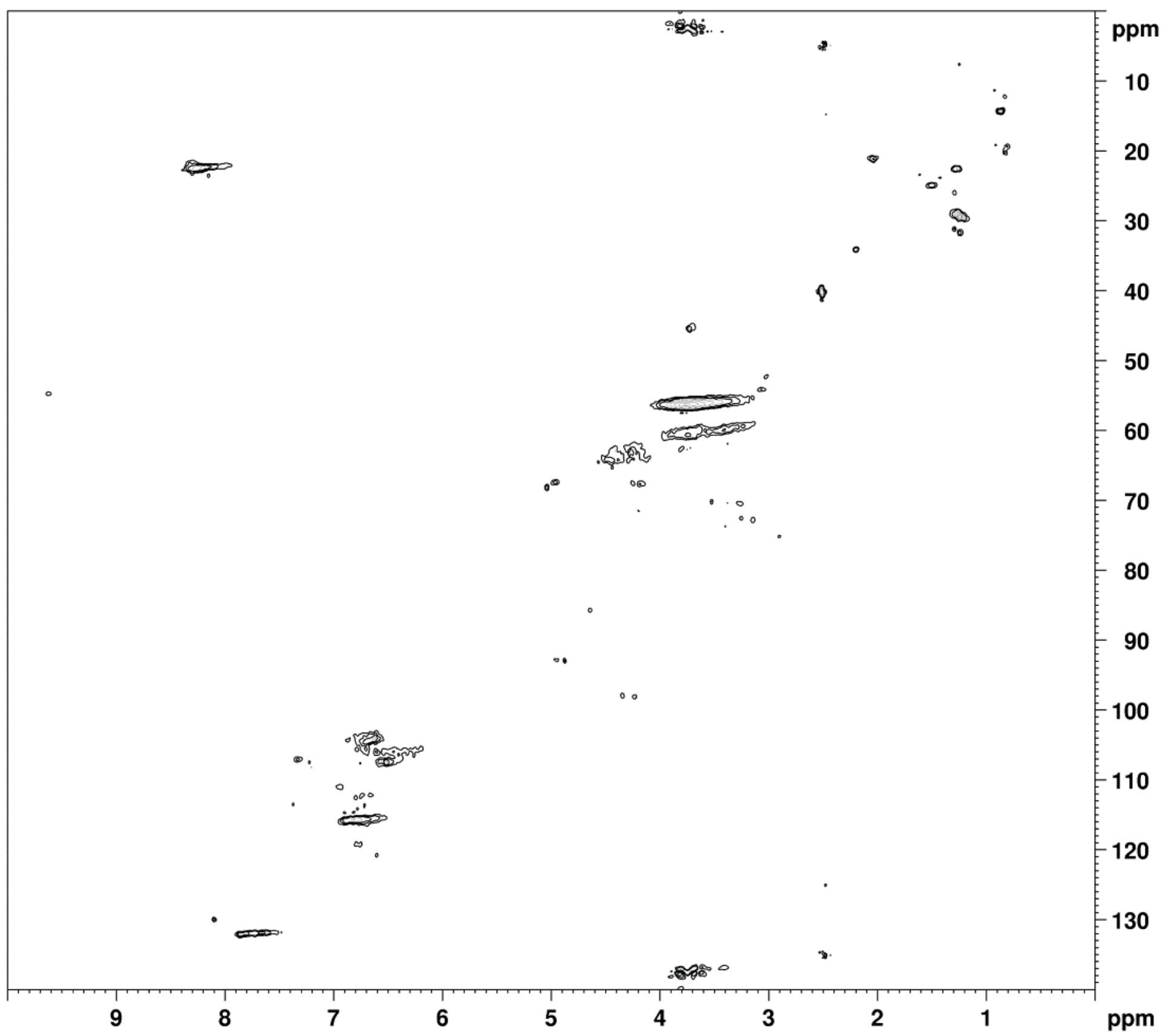

Fig. S1: ^{1}H–^{13}C HSQC NMR comparison of FA-only fractionated lignin. FA-only lignin was obtained from formic-acid fractionation in the absence of syringol. Compared with SL, FA-only lignin did not show the diagnostic Cα-syringolated correlations and exhibited negligible β–O–4 correlations, indicating that syringol is required to selectively trap the reactive Cα intermediate and suppress non-selective acid-induced lignin condensation.

2. The authors suggest that the composites could be used packaging materials, which need some additional data for this application.

Response: We thank the reviewer for this helpful comment. We agree that additional performance data are necessary to support the proposed packaging application.

To address this point, we have supplemented the evaluation of ESLP with wet mechanical testing, surface wettability, long-term humidity aging, thermal stability, composting degradation and a comparison with representative packaging materials. ESLP exhibited a dry tensile strength and modulus of 46.7 MPa and 2.5 GPa, respectively, while retaining a wet tensile strength of 33.9 MPa and a wet modulus of 2.0 GPa. The initial water contact angle increased from 75.8° for neat pulp to 88.4° for ESLP and remained at 85.0° after 60 s, indicating reduced surface wettability.

Long-term stability was evaluated by aging ESLP for 97 days at 25–85% RH. The aged samples retained tensile strengths of 25.9–28.3 MPa across the investigated humidity range. In addition, the final ESLP composite showed a DTG maximum at 384.8 °C, demonstrating good thermal stability.

The end-of-life behavior was evaluated through complementary compost-burial and CO_2-evolution experiments. ESLP showed progressive surface deterioration, deformation and partial fragmentation during 72 days of compost burial, while the blank-corrected cumulative CO_2 evolution reached 920.7 mg after 65 days. A systematic comparison with representative PLA, cellulose nanopaper, kraft paper and PET has also been added as Table S7.

These additional results demonstrate that ESLP combines packaging-relevant mechanical strength, high wet-strength retention, long-term humidity stability, thermal stability and progressive degradation under composting conditions.

The corresponding discussion has been included in the revised manuscript, shown as below:

Page 15–17

While the contact-angle and wet-mechanical measurements demonstrate the immediate resistance of ESLP to water exposure, its practical use also requires retention of mechanical integrity during prolonged exposure to humid environments. The long-term humidity stability of ESLP was further evaluated after aging for 97 days at 25–85% RH (Fig. 2a–b). The aged samples retained tensile strengths of 25.9–28.3 MPa, corresponding to approximately 55–61% of the original value, with no clear humidity-dependent decrease across the investigated range. In contrast, the tensile modulus remained approximately 2.0 GPa after aging at 25% and 55% RH but decreased to 1.28 and 1.01 GPa at 75% and 85% RH, respectively. The lower modulus observed after aging at 75% and 85% RH suggests that prolonged high-humidity exposure induced residual or only partially reversible changes in the cellulose-rich network. Nevertheless, the substantial tensile strength retained after 97 days indicates that the covalently coupled ESL–cellulose network maintains long-term load-bearing integrity under prolonged humidity exposure.

The moisture-sorption behavior of ESLP during long-term humidity exposure was also monitored (Fig. S13). Water uptake increased rapidly during the initial stage and subsequently approached a relatively stable level within the first few days. As expected, the water uptake increased with relative humidity, reaching approximately 1.5–2.0%, 4.1–4.5%, 5.0–5.5% and 6.8–7.3% under nominal RH conditions of 25%, 55%, 75% and 85%, respectively. After the initial increase, no pronounced continuous mass gain was observed during the remaining exposure period, indicating that the specimens reached a relatively stable moisture-sorption state. The humidity-dependent water uptake confirms that the cellulose-rich composite remains slightly hygroscopic, particularly at elevated RH, where absorbed moisture can promote plasticization and increase molecular and inter-fiber mobility. Nevertheless, the limited increase in water uptake over prolonged exposure suggests that the covalently coupled ESL–cellulose structure restricted progressive moisture-induced disruption of the material.

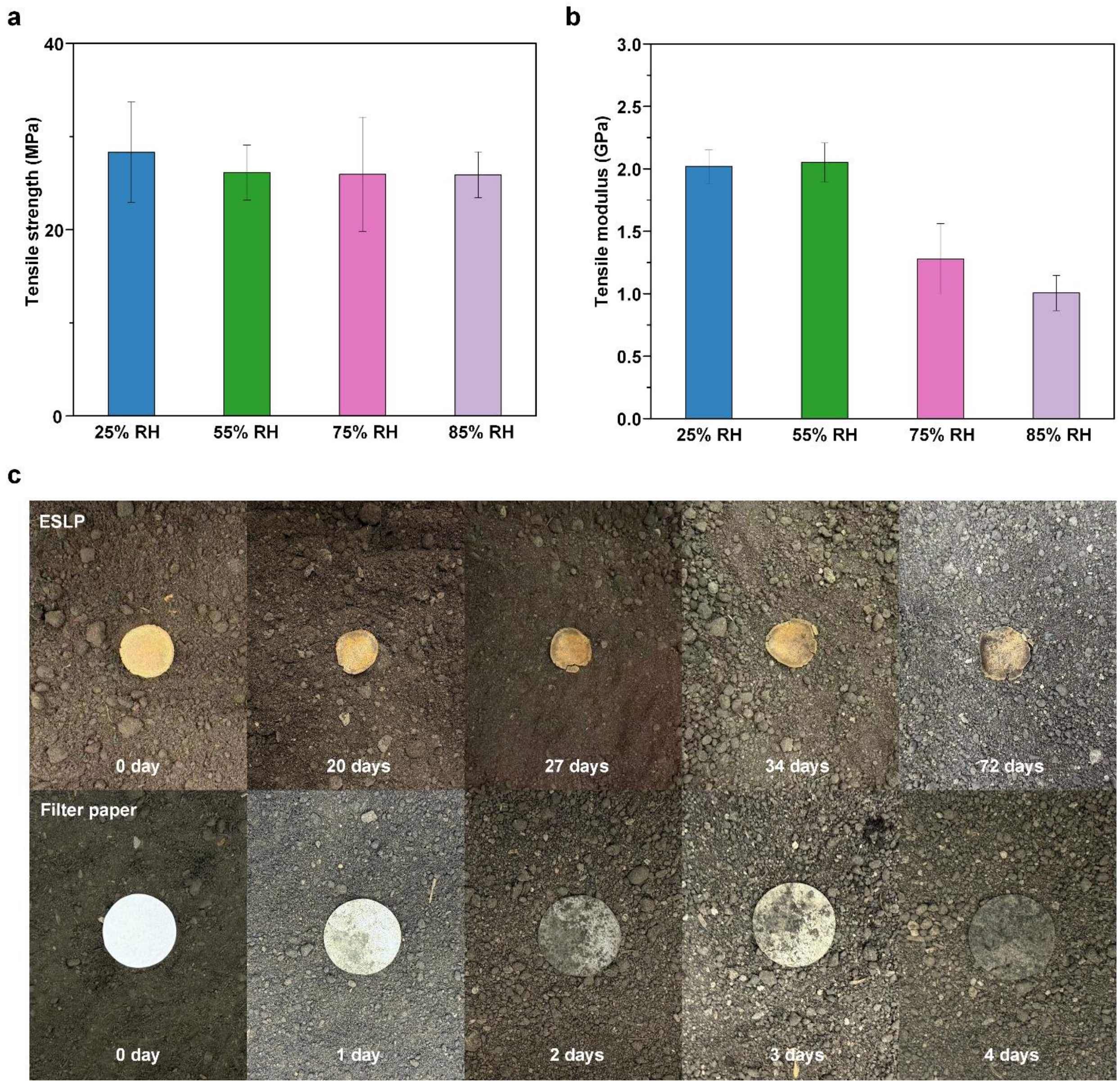

Fig. 4: Long-term humidity stability and composting degradation behavior of ESLP. a Tensile strength and **b** tensile modulus of ESLP after aging for 97 days at different relative humidities at room temperature. **c** Representative photographs showing the macroscopic disintegration of ESLP and filter paper during composting.

Having established that ESLP reached a stable moisture-sorption state while retaining substantial mechanical integrity during prolonged humidity exposure, we next examined whether this service-stage durability could coexist with biological degradation under an appropriate end-of-life environment. In the compost-burial experiment, the filter-paper control underwent rapid macroscopic deterioration within four days and was no longer visually detectable after five days, whereas ESLP showed gradual surface discoloration, deformation, erosion and partial fragmentation over 72 days (Fig. 2c). The slower disintegration of ESLP is consistent with its

lignin-containing and covalently coupled network. In a separate compost-respiration experiment, the blank-corrected cumulative CO_2 evolution increased progressively and reached 920.7 mg after 65 days (Fig. S14), indicating enhanced microbial respiration associated with ESLP addition. Because the measurement was based on extrapolation of a 2 h respiration rate to a long-term equivalent, and the ESLP addition may stimulate the decomposition of native compost organic matter, the cumulative value is not interpreted as an absolute value derived exclusively from ESLP carbon[65–67]. Thus, the burial and respiration experiments provide complementary evidence of macroscopic disintegration and biological degradation, respectively. These results indicate that ESLP can maintain functional integrity under service-relevant humidity conditions while undergoing progressive degradation under an appropriate composting environment.

Page 17–18

To contextualize the packaging potential of ESLP, its performance was compared with representative PLA, lignin-containing cellulose nanopaper, kraft paper and PET film (Table S7). ESLP exhibited a tensile strength and modulus of 46.7 MPa and 2.5 GPa, respectively, which are within the representative ranges of widely used packaging materials. Its distinguishing feature is its moisture-resistant mechanical performance: ESLP retained 33.9 MPa wet tensile strength and a wet modulus of 2.0 GPa, corresponding to 72.6% and 80.0% retention, respectively. These strength-retention values are higher than those reported for the representative lignin-containing cellulose nanopaper and kraft paper included in the comparison. ESLP also showed a DTG maximum at 384.8 °C (Fig. S15) and retained tensile strengths of 25.9–28.3 MPa after 97 days of humidity aging. Together with its progressive macroscopic deterioration and blank-corrected CO_2 evolution under composting conditions, these results demonstrate a balance between service-stage moisture stability and end-of-life biological degradability.

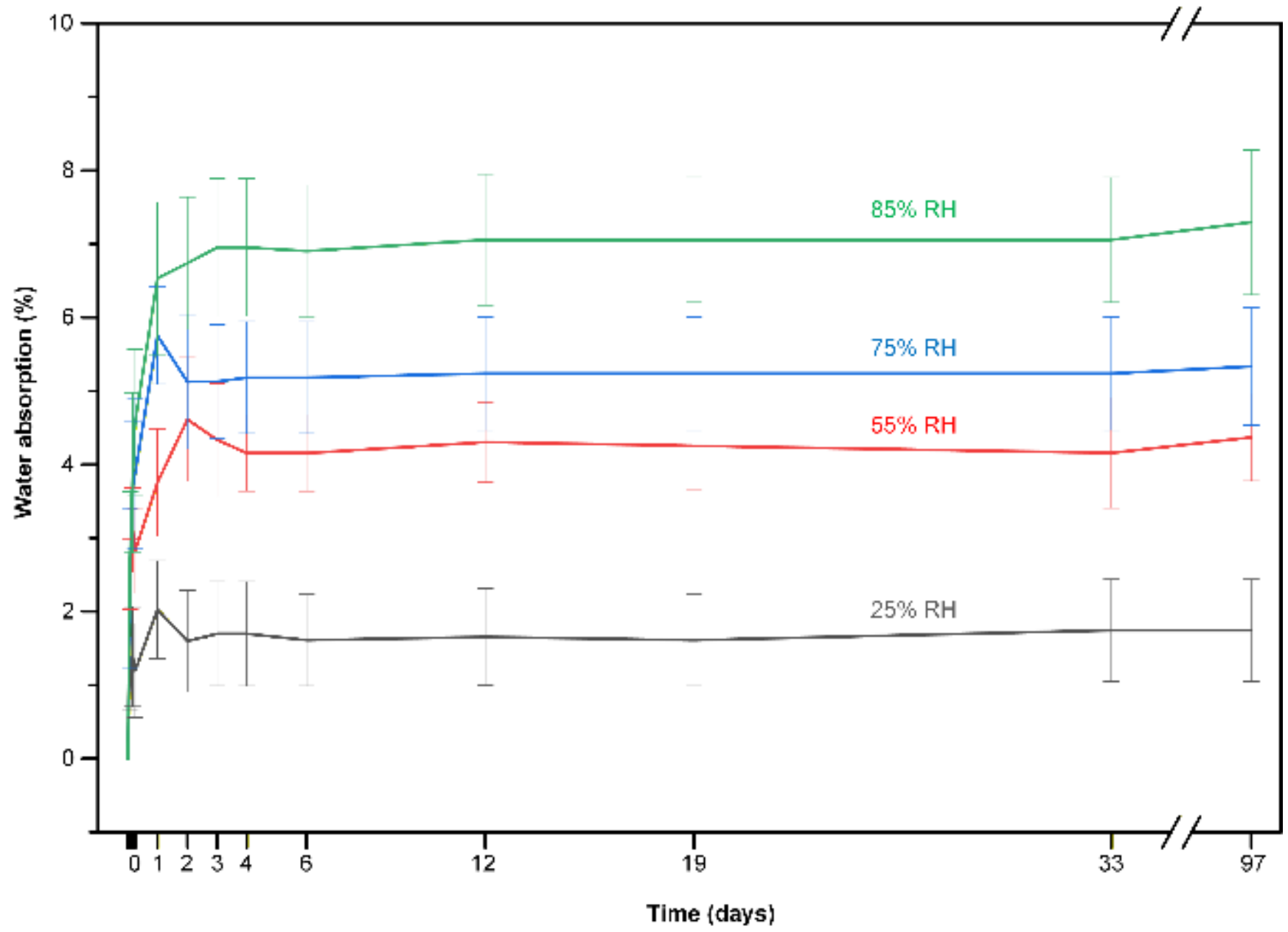

Fig. S13: Water uptake of ESLP during long-term humidity aging. Water uptake of ESLP exposed to saturated potassium acetate, magnesium nitrate, sodium chloride and potassium chloride solutions, corresponding to nominal relative humidities of approximately 25%, 55%, 75% and 85%, respectively, at room temperature.

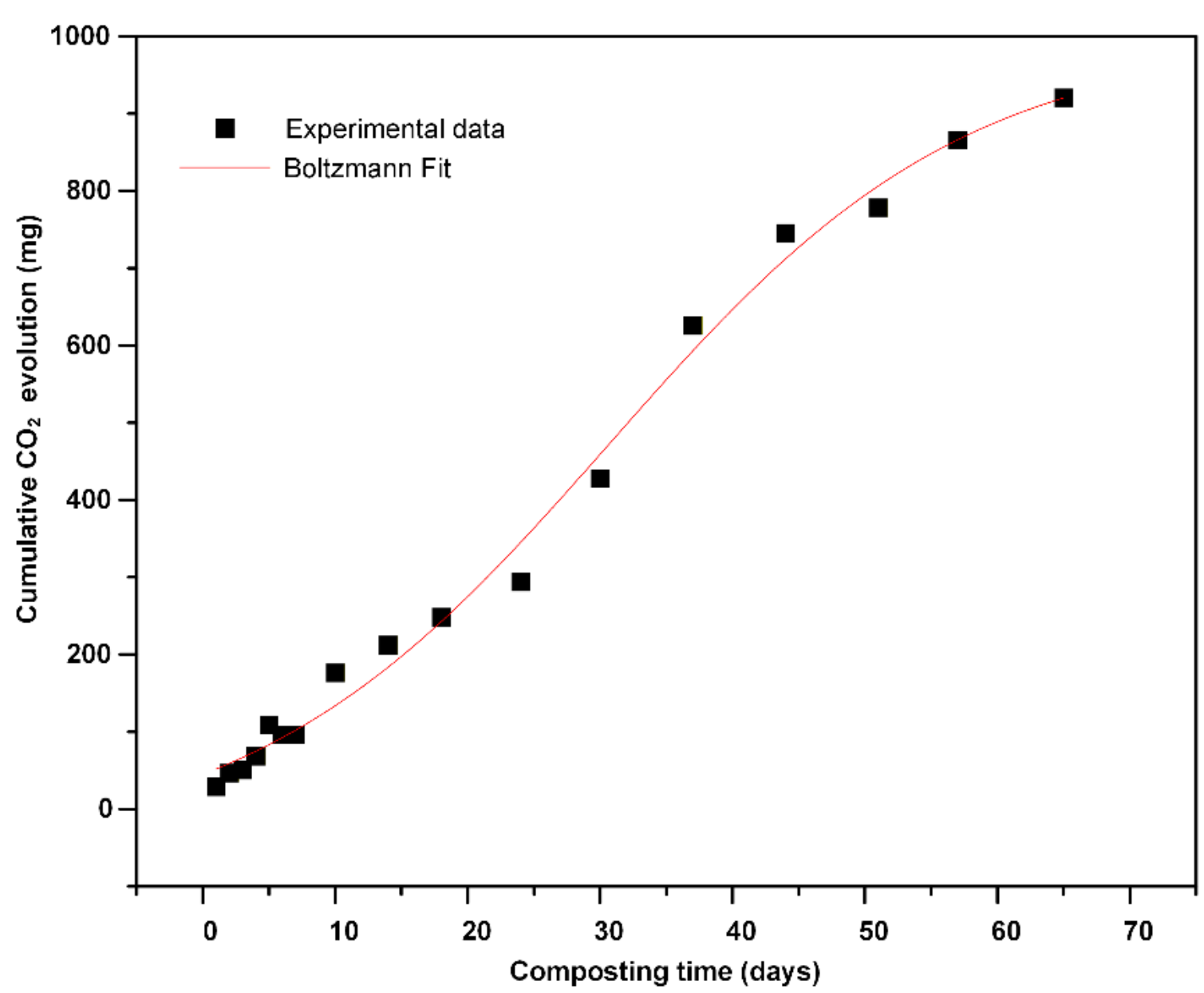

Model	Boltzmann
Equation	y = A2 + (A1-A2)/(1 + exp((x-x0)/dx))
Plot	Cumulative CO2 evolution
A1	-53.60869 ± 57.53754
A2	996.96416 ± 58.95123
x0	30.64351 ± 1.65377
dx	13.51516 ± 2.31862
Reduced Chi-Sqr	836.54621
R-Square (COD)	0.99331
Adj. R-Square	0.99177

Fig. S2: Cumulative CO_2 evolution from ESLP during compost incubation. Cumulative net CO_2 evolution during 65 days of composting after subtraction of the compost blank. The symbols represent the experimental data, and the line represents an empirical sigmoidal fit.

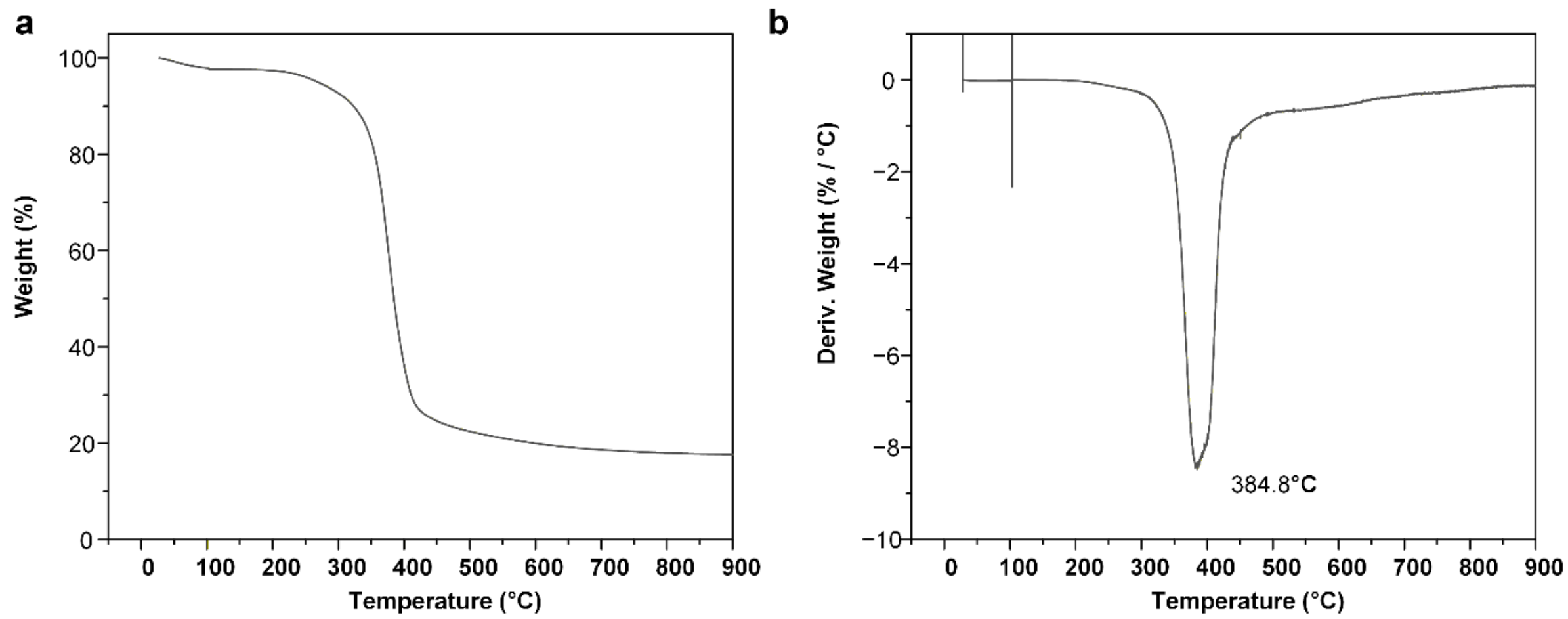

Fig. S3: TG and h DTG curves of ESLP, represent the thermal stability of sample. The main DTG maximum occurs at 384.8 °C.

Table S8. Comparison of ESLP with representative bio-based and conventional packaging materials.

Material	Tensile strength (MPa)	Tensile modulus (GPa)	Wet or humidity stability	Thermal behavior	Degradation or end-of-life behavior	Ref.
ESLP	46.7	2.5	Wet tensile strength: 33.9 MPa with 72.6% retention; wet modulus: 2.0 GPa with 80.0% retention. After 97 days at 25–85% RH, tensile strength remained at 25.9–28.3 MPa.	DTG T_{max} at 384.8°C.	Progressive surface deterioration, deformation and partial fragmentation during 72 days of compost burial; blank-corrected cumulative CO_2 evolution reached 920.7 mg after 65 days.	This work
PLA, representative neat film	58.2	1.05	NR under a directly comparable water-immersion tensile test	Degradation onset: 211.9 °C; DTG T_{max}: 324.8 °C	A representative PLLA formulation reached 100.2 ± 3.7% biodegradation and complete disintegration under controlled industrial-composting conditions; compostable bags fully decomposed after seven weeks.	[15,16]
Lignin-containing	255	6.4	Wet tensile strength: 83 MPa, corresponding to	DTG T_{max}: 339 °C	NR in the cited study	[17]

Material	Tensile strength (MPa)	Tensile modulus (GPa)	Wet or humidity stability	Thermal behavior	Degradation or end-of-life behavior	Ref.
cellulose nanopaper			approximately 32.5% retention			
Kraft paper, representative literature values	55.8	NR	Wet tensile strength: 2.3 MPa, corresponding to approximately 4.1% retention	Major thermal mass loss occurred at approximately 280–350 °C, with a T_{max} near 350 °C.	Paper products are biodegradable under composting conditions, although the degradation rate depends strongly on pulping history, lignin content and additives.	18–20
PET film	48.3–72.4	2.76–4.14	NR under a directly comparable water-immersion tensile test	DTG maximum: 441 °C	Not conventionally compostable; biological depolymerization generally requires specialized enzymes or microbial systems.	21–23

3. It's not clear whether the cellulose crystallinity has been changed before and after the reaction with lignin in the composites.

Response: We thank the reviewer for this helpful comment. We agree that the cellulose crystallinity before and after reaction with ESL should be clarified. To address this point, we supplemented XRD analysis of native poplar, fractionated pulp and ESLP, and calculated the relative crystallinity index (*CrI*) using the Segal method.

The XRD patterns show that the characteristic cellulose I diffraction features were retained after fractionation and ESLP formation, indicating that the cellulose crystalline framework was not destroyed during hot pressing and ESL incorporation. The relative crystallinity index increased

from 43.3% for native poplar to 57.7% for the fractionated pulp, which is consistent with the removal of amorphous lignin and hemicellulose during reactive fractionation. After ESL incorporation and hot pressing, the CrI slightly decreased to 53.4%, but remained higher than that of native poplar.

This slight decrease from pulp to ESLP should be interpreted as an apparent relative crystallinity change of the composite, mainly because amorphous ESL contributes to the XRD background intensity. Therefore, the result does not indicate destruction or transformation of cellulose crystallites. Instead, the XRD data show that the cellulose crystalline framework is largely retained in ESLP. The enhanced dry and wet mechanical properties are therefore mainly attributed to covalent ESL–cellulose coupling and improved inter-fiber adhesion rather than increased cellulose crystallinity.

The new XRD patterns and *CrI* values have been added as Fig. S12b in the Supporting Information, and the corresponding discussion has been included in the revised manuscript, shown as below:

Page 15

XRD analysis was further performed to examine whether the cellulose crystalline structure changed after fractionation and ESLP formation (Fig. S12b). The relative crystallinity index increased from 43.3% for native poplar to 57.7% for the fractionated pulp, consistent with the removal of amorphous lignin and hemicellulose during reactive fractionation. After ESL incorporation and hot pressing, ESLP showed a relative crystallinity index of 53.4%, slightly lower than that of pulp but still higher than that of native poplar. This slight decrease is attributed to the amorphous ESL phase contributing to the composite background rather than the destruction of the

cellulose crystalline domains. These results indicate that the cellulose crystalline framework was retained in ESLP, while the improved mechanical and wet mechanical performance mainly arises from covalent ESL–cellulose coupling and enhanced inter-fiber adhesion rather than increased cellulose crystallinity.

SI

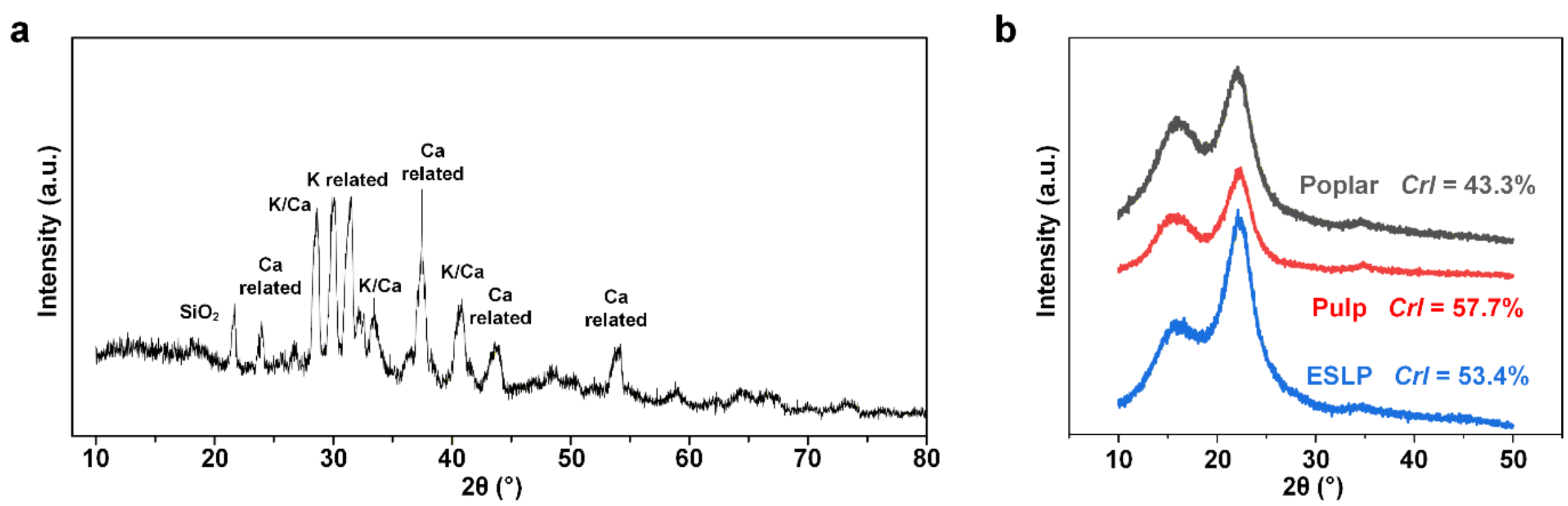

Fig. S12: XRD analysis of cellulose crystallinity and rapeseed straw ash. a XRD pattern of rapeseed straw ash. The major diffraction features are tentatively assigned to SiO_2-related phases, K-related salts, Ca-related phases and overlapping K/Ca-containing inorganic phases. **b** XRD patterns of native poplar, fractionated pulp and ESLP.

4. The hot-pressing at 180C and 3.5 MPa for 1 hour is a harsh condition compared to conventional lignocellulosic materials. The authors need to justify if his is an optimum condition.

Response: We thank the reviewer for this important comment, and we apologize that the rationale for the hot-pressing condition was not sufficiently explained in the original manuscript. We agree that hot-pressing at 180 °C and 3.5 MPa for 1 h may appear relatively harsh compared with some conventional lignocellulosic material-processing conditions, and we have revised the manuscript to clarify the purpose and limitation of this condition. In this work, the curing temperature was

selected based on the ESL–filter paper model experiment. The tensile strength increased with curing temperature and reached the highest value at 180 °C, whereas further increasing the temperature led to decreased mechanical performance, likely due to thermal damage or degradation of the cellulose network. Therefore, 180 °C was selected as the curing temperature to promote epoxide ring-opening between ESL and cellulose while avoiding excessive thermal degradation.

Regarding the pressure, we attempted to identify a lower-pressure hot-pressing condition for ESLP fabrication. However, due to the operational limitation of our hot-pressing equipment, 3.5 MPa was the lowest loading pressure that could be reliably detected, applied and maintained during the program. When lower pressures were set, the instrument could not accurately detect the applied load and the hot-pressing program failed. We apologize for this limitation.

Importantly, the covalent ESL–cellulose coupling itself does not require high external pressure. In the ESL–filter paper model experiment, no external hot-pressing pressure was applied, yet a substantial increase in tensile strength was still observed after thermal curing in the presence of DMAP. This result indicates that the mechanical enhancement originates primarily from the thermally promoted epoxide ring-opening reaction and covalent ESL–cellulose linkage, while pressure mainly assists sheet consolidation and inter-fiber contact in the pulp-based composite.

Reviewer #4:

This manuscript presents a compelling and timely strategy to co-valorize lignin and cellulose from a single lignocellulosic feedstock via metal-free reactive fractionation, followed by epoxy-functionalization and covalent coupling to produce composites with strong wet-strength retention. The overall approach is innovative and the results are promising. I recommend publication after the authors address the following minor points to improve clarity and strengthen the mechanistic and structural interpretation:

Response: We sincerely thanks the reviewer's recommendation.

1. The authors provide HSQC spectra of SL and ESL; however, for a more complete structural comparison, the HSQC features of native poplar lignin should be discussed.

Response: We thank the reviewer for this helpful suggestion. We agree that discussing the HSQC features of native poplar lignin provides a clearer structural baseline for evaluating the changes induced by reactive fractionation and subsequent epoxy-functionalization. To address this point, we have added the HSQC spectrum of native poplar lignin as Figure S1 in the Supporting Information and included a corresponding discussion in the revised manuscript. The native poplar lignin spectrum shows typical hardwood lignin features, including syringyl and guaiacyl aromatic correlations and β–O–4 interunit-linkage signals. In particular, the native β–O–4 Cα–Hα

correlation is visible in the oxygenated aliphatic region, representing the original benzylic Cα–OH structure of native lignin. After syringol-assisted reactive fractionation, this native Cα–OH signal disappeared, while new correlations assigned to substituted Cα positions appeared in SL, confirming that syringol intercepted the benzylic intermediate and formed syringolated lignin. After epoxy-functionalization, additional aliphatic cross-peaks assigned to epoxy methylene and methine groups appeared in ESL, while the syringolated lignin backbone was retained. This comparison more clearly demonstrates the structural evolution from native poplar lignin to SL and then ESL. The new discussion has been added to Page 6, and the native poplar lignin HSQC spectrum has been added as Figure S1. The revised manuscript is shown as below:

Page 6

The SL was isolated by precipitation in 35.8% yield based on initial poplar (theoretical maximum 38%). Running the reactive fractionation at reflux afforded a slightly brownish off-white solid (**Error! Reference source not found.**a), whereas lowering the temperature to 80 °C produced a whiter lignin but led to incomplete syringolation and thereby a lignin with reduced reactivity. The comparison between native poplar lignin and SL by heteronuclear single-quantum coherence (HSQC) NMR indicated successful arylation. For a clearer structural comparison, the HSQC features of native poplar lignin were first examined (Fig. S1). The native poplar lignin spectrum showed typical hardwood lignin correlations. In particular, the native β–O–4 Cα–Hα correlation at δC/δH = 72.6/4.8 ppm, corresponding to the benzylic Cα–OH structure of native lignin, was clearly observed. After syringol-assisted reactive fractionation, this native Cα–OH correlation disappeared, while new correlations at δC/δH = 44.8/4.7 and 53.9/4.2 ppm appeared, consistent with substituted Cα sites formed upon syringolation (**Error! Reference source not found.**a)[42]. This comparison confirms that syringol effectively trapped the benzylic intermediate during fractionation and converted native lignin into syringolated lignin.

SI

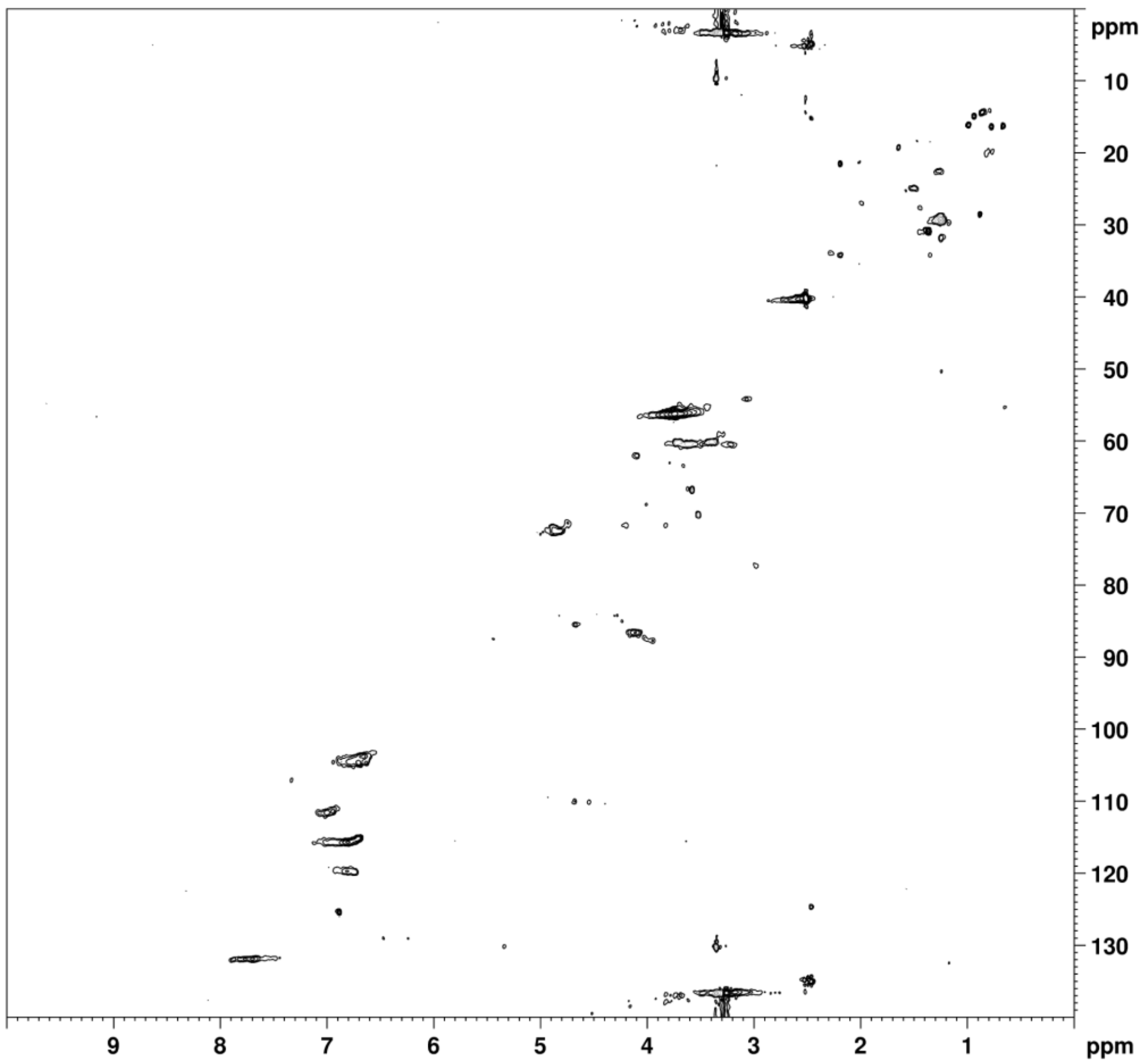

Fig. S4: ^{1}H–^{13}C HSQC NMR spectrum of native ball milled lignin of poplar. The spectrum shows typical hardwood lignin correlations, including syringyl and guaiacyl aromatic signals and β–O–4 interunit-linkage signals. The native β–O–4 Cα–Hα correlation provides a structural reference for comparison with syringolated lignin.

2. The composite is prepared using an ESL/pulp mass ratio of 1:1. Did the authors explore other ratios?

Response: We thank the reviewer for this important question. Preliminary composition screening was performed using the ESL–filter paper model system. Initial ESL/filter-paper mass ratios of 1:1, 1.5:1 and 2:1 were evaluated. After thermal curing and acetone washing to remove unreacted or

non-covalently retained ESL, the final ESL loadings of the three samples were all close to an ESL/cellulose mass ratio of approximately 1:1. Increasing the initial ESL amount above 1:1 therefore did not result in a corresponding increase in the retained ESL content, indicating that the accessible cellulose-fiber surface had approached its effective loading capacity. The excess ESL mainly increased reagent consumption and the amount removed during purification.

Based on these preliminary results, an ESL/pulp mass ratio of 1:1 was selected for preparation of the pulp-based composite as a practical formulation that provided sufficient ESL for fiber coverage and interfacial coupling while avoiding unnecessary excess resin. We have clarified this rationale in the revised manuscript. The 1:1 ratio is regarded as the selected formulation for the present proof-of-concept study rather than a comprehensive optimization of all possible ESL/pulp compositions.

The curing process was first optimized using filter paper as a cellulose model. ESL (0.20 g) and DMAP (2 wt% of ESL) were dissolved in acetone (5 mL), in which 0.20 g of filter paper was immersed. After air drying, the impregnated filter paper was heated at 80–220 °C for 1 h. The resulting ESLFP was washed with acetone under sonication for 5 min to remove non-covalently bound ESL. A control group was prepared without DMAP, named as ESLFP*. The ESL/pulp mass ratio of 1:1 was selected based on preliminary ESL–filter paper loading experiments, in which increasing the initial ESL/cellulose ratio from 1:1 to 1.5:1 or 2:1 did not appreciably increase the retained ESL content after acetone washing.

3. The authors reported a meta/para ratio for SL obtained from reactive fractionation. Please provide a brief explanation of how this ratio is determined and what it implies.

Response: We thank the reviewer for this helpful comment. We agree that the determination and

implication of the meta/para ratio should be clarified. In the revised manuscript, we have added a brief explanation of how this ratio was obtained and what it represents. The corresponding explanation has been added to Page 6 of the revised manuscript, shown as below:

Page 6

The *meta/para* (*m/p*) ratio of SL was estimated from the HSQC spectrum by integrating the diagnostic Cα–Hα correlations corresponding to meta- and para-syringolated β–O–4 structures. The calculated m/p ratio was approximately 2:1, indicating that syringol addition occurred at both regioisomeric positions, with the meta-substituted structure being more abundant under the present reactive fractionation conditions. This regioisomeric distribution further supports the nucleophilic trapping of the benzylic Cα intermediate by syringol during fractionation.

4. Hot-pressing and densification can themselves improve the mechanical strength of cellulose networks. How did the authors distinguish the contribution from densified morphology versus covalent ESL–cellulose linkages.

Response: We thank the reviewer for this insightful comment. We agree that hot-pressing-induced densification can contribute to the mechanical strength of cellulose networks. We have revised the manuscript to clarify this point by explicitly identifying the neat pulp sheet as a hot-pressed control.

In our experiments, the neat pulp sheet was prepared under the same hot-pressing conditions as ESLP but without ESL. Therefore, it represents the contribution from cellulose-network densification. Although the hot-pressed pulp formed a compact sheet, its tensile strength and modulus were only 8.5 MPa and 0.8 GPa, respectively, much lower than those of ESLP, which

reached 46.7 MPa and 2.5 GPa. The difference was even more pronounced under wet conditions: the hot-pressed pulp retained only 1.9 MPa wet tensile strength and 0.2 GPa wet modulus, whereas ESLP retained 33.9 MPa and 2.0 GPa. This comparison indicates that densification contributes to sheet formation and dry strength, but cannot account for the large wet-strength retention of ESLP. We have therefore revised the text to clarify that the enhanced performance of ESLP arises from the combined effect of hot-pressing-induced consolidation and covalent ESL–cellulose coupling, with the covalent interphase being particularly important for maintaining load-bearing performance under wet conditions, shown as below:

Page 13

The resulting ESLP exhibited a tensile strength of 46.7 MPa and modulus of 2.5 GPa, markedly higher than the neat pulp sheet prepared under the same hot-pressing conditions (8.5 MPa and 0.8 GPa, respectively) (**Error! Reference source not found.**a–c). These gains confirm that the covalent-bonding strategy operative in the model system also functions with fractionated pulp, demonstrating concurrent valorization of lignin and cellulose into one material. ESLP maintained 33.9 MPa wet tensile strength and a wet modulus of 2.0 GPa, whereas pure pulp retained only 1.9 MPa and 0.2 GPa (**Error! Reference source not found.**d–f). Because the neat pulp control was subjected to the same hot-pressing process, this comparison indicates that densification alone cannot account for the large improvement in mechanical performance, especially under wet conditions. The high wet-strength retention of ESLP therefore supports that covalent ESL–cellulose coupling, rather than hydrogen bonding alone, governs the durable inter-fiber adhesion in moist environments.

5. The strategy compellingly co-utilizes cellulose and lignin from one feedstock. Given the manuscript's "whole-biomass" framing, what's the fate of the hemicellulose fraction in this process?

Response: We thank the reviewer for raising this important point. We have revised the manuscript

to clarify the fate of hemicellulose during the reactive fractionation process. During formic-acid-based fractionation, hemicellulose is largely removed from the solid cellulose stream and transferred to the hydrophilic filtrate, most likely as xylose and xylose-derived soluble species, as reported before. Previous studies have shown that xylose can be converted to formic acid, suggesting a potential route for future process integration; this conversion and solvent recycling were not demonstrated in the present study. We have revised the manuscript to explain the role of hemicellulose in this strategy, shown as below:

Page 4

The pulp contained 94.2% cellulose and 1.9% hemicellulose, with 3.3% lignin. By comparison, native poplar comprised 47.5% cellulose, 17.1% hemicellulose and 28.1% lignin (Table S1). The low residual hemicellulose content in the pulp indicates that hemicellulose was largely hydrolyzed into xylose and xylose-derived soluble species and transferred to the hydrophilic filtrate during the formic-acid-based fractionation[42]. In addition, xylose has been demonstrated as a suitable feedstock for formic acid production, providing a potential route for future process integration and solvent regeneration[43].

Review Comments

This work proposes a metal-free, ambient-pressure reactive fractionation strategy that enables the simultaneous valorization of cellulose and lignin in lignocellulosic feedstocks, breaking through the limitation of prioritizing a single component (either cellulose or lignin) in traditional biomass conversion to truly achieve holistic biomass co-valorization; through in-situ syringolation modification of lignin and subsequent epoxy functionalization, epoxy-functionalized syringolated lignin (ESL) is constructed, and by forming covalently bonded composites with cellulose via epoxide ring-opening reaction, the material's wet mechanical stability is significantly improved, overcoming the inherent defect of deteriorated wet performance in traditional hydrogen-bonded cellulose materials; additionally, the technical route exhibits good tolerance to ash content in biomass feedstocks, allowing direct use of low-quality raw materials such as forestry residues (e.g., poplar tops and branches) and agricultural wastes (e.g., rapeseed straw) with high ash content, broadening the scope of feedstocks for biomass conversion and solving the key problem of catalyst poisoning by ash in traditional metal-catalyzed conversion routes.

Furthermore, the work provides a new technical paradigm for holistic biomass co-valorization, clarifies the reaction mechanism of lignin-cellulose covalent bonding, and enriches the preparation theory of biomass-based composites; the prepared composites exhibit mechanical properties comparable to traditional packaging materials (e.g., kraft liner and PET) under both dry and wet conditions, offering a feasible solution for the bio-based substitution of fossil-derived packaging materials and aligning with global sustainable development strategies; additionally, the technical route features good scalability with mild reaction conditions (ambient pressure, no metal catalysts), lowering the threshold for industrial application and holding great significance for promoting the efficient utilization of biomass resources and the development of green material industries.

Overall, this study proposes an innovative holistic biomass co-valorization technology and successfully prepares lignin-cellulose covalent composites with excellent wet stability. The research topic has important scientific significance and application value. The experimental design is reasonable, the characterization methods are comprehensive, the data are sufficient and reliable, and the conclusions are clear and powerful. Although there are deficiencies in process parameter optimization, long-term stability evaluation, degradation performance research, and in-depth exploration of reaction mechanism, these can be effectively solved by appropriately supplementing experiments and improving analysis.

In summary, The core innovations of the study meet the publication requirements of Nature Communications, but targeted supplements and improvements are needed in terms of resource recycling, function expansion, and process practicality.

Specific decision: **Major Revision.**

Below are detailed comments and suggestions for improvement:.

1. Research Methods and Experimental Design

(1) The optimization of syringol dosage as a nucleophile in the reactive fractionation process is insufficiently reflected. It is recommended to supplement data on the effects of different syringol/biomass ratios on fractionation efficiency (cellulose purity, lignin extraction rate) and lignin modification effect, so as to clarify the basis for selecting optimal process parameters.

(2) The long-term stability evaluation of the composites is inadequate. The existing data only

reflect short-term wet mechanical properties. It is suggested to supplement long-term aging experiments (e.g., 1-3 months) of the materials under different humidity environments to investigate the stability of the covalently bonded structure and the attenuation law of mechanical properties.

(3) A comprehensive performance comparison with commercial bio-based packaging materials is lacking. It is recommended to add a systematic comparison with existing mainstream biomass-based packaging materials (e.g., polylactic acid, cellulose-based composites) in terms of mechanical properties, thermal stability, gas permeability, and degradation performance, to more intuitively highlight the application advantages of the materials in this study.

2. Research Methods and Experimental Design

(1) The optimization of syringol dosage as a nucleophile in the reactive fractionation process is insufficiently reflected. It is recommended to supplement data on the effects of different syringol/biomass ratios on fractionation efficiency (cellulose purity, lignin extraction rate) and lignin modification effect, so as to clarify the basis for selecting optimal process parameters.

(2) The long-term stability evaluation of the composites is inadequate. The existing data only reflect short-term wet mechanical properties. It is suggested to supplement long-term aging experiments (e.g., 1-3 months) of the materials under different humidity environments to investigate the stability of the covalently bonded structure and the attenuation law of mechanical properties.

(3) A comprehensive performance comparison with commercial bio-based packaging materials is lacking. It is recommended to add a systematic comparison with existing mainstream biomass-based packaging materials (e.g., polylactic acid, cellulose-based composites) in terms of mechanical properties, thermal stability, gas permeability, and degradation performance, to more intuitively highlight the application advantages of the materials in this study.

(4) The in-depth exploration of the reaction mechanism needs to be strengthened. Although XPS and FTIR confirm epoxide ring-opening and covalent bond formation, quantitative analysis of reaction kinetics (e.g., reaction rate constant, activation energy) is lacking. It is suggested to supplement reaction kinetic experiments at different temperatures to further improve the theoretical interpretation of the reaction mechanism.

3. Data Quality and Result Analysis

(1) The presentation of some characterization data is not detailed enough. For example, in GPC analysis, specific values of number-average molecular weight (Mn), weight-average molecular weight (Mw), and dispersity (Đ) are not provided, and only the molecular weight change is explained through curve trends. It is recommended to supplement specific data to enhance persuasiveness; the assignment of some characteristic peaks in FTIR spectra (e.g., the C-H vibration peak of the epoxy ring around 3056 cm^{-1}) can be further supported by combining with literature data.

(2) The analysis of the influence mechanism of ash on the reaction process is not in-depth enough. Although the technology is mentioned to be tolerant to high ash content, the existing form of ash (e.g., alkali metal and alkaline earth metal compounds) in the reaction and its specific influence on reactive fractionation, epoxy functionalization, and other links (e.g., whether it participates in the reaction, whether it affects product purity) are not clarified. It is recommended to supplement relevant characterizations (e.g., ICP-OES analysis of ash element composition, XRD analysis of ash phase) and control experiments to clarify the internal reason for ash tolerance.

(3) The degradation performance of the composites is not involved. As a potential packaging material, biodegradability is an important evaluation index. It is recommended to supplement degradation experiments of the materials under natural environment or composting conditions to investigate their environmental friendliness and improve the performance evaluation system of the materials.

4.Conclusions and Discussion

(1) The comparison between this technology and "lignin-first" strategies (e.g., reductive catalytic fractionation, RCF) in the discussion is not comprehensive enough. It is recommended to conduct a systematic comparison from aspects of feedstock adaptability, process complexity, product added value, and industrialization cost to further highlight the competitive advantages of this technology.

(2) The discussion on the expansion of application scenarios for the composites is insufficient. The existing discussion mainly focuses on packaging materials. It is recommended to explore their potential applications in other fields (e.g., biomedical materials, agricultural mulch films, water treatment membranes) combined with the performance characteristics of the materials (e.g., wet stability, mechanical strength), so as to broaden the application perspective of the research.

(3) The prospect of industrialization of the technology in the conclusion section is too general. It is recommended to supplement the analysis of potential challenges in the industrialization process (e.g., raw material pretreatment, product separation and purification, cost control) and propose preliminary solutions to enhance the practical application guiding significance of the research.
